# Alkynyltriazenes in Photochemical Metal‐Free Doyle–Kirmse Rearrangements

**DOI:** 10.1002/anie.202521012

**Published:** 2026-01-10

**Authors:** Ningning Liu, Linus Bjarne Dittmer, Melina Maag, Elena Michel, Frank Rominger, Matthias Rudolph, Andreas Dreuw, A. Stephen K. Hashmi

**Affiliations:** ^1^ Organisch‐Chemisches Institut Heidelberg University Im Neuenheimer Feld 270 Heidelberg 69120 Germany; ^2^ Interdisziplinäres Zentrum für Wissenschaftliches Rechnen (IWR) Heidelberg University Im Neuenheimer Feld 205 Heidelberg 69120 Germany; ^3^ Chemistry Department Faculty of Science King Abdulaziz University Jeddah 21589 Saudi Arabia

**Keywords:** Cyanocarbenes, Nitrile compounds, Photochemistry, Rearrangement, Triazenes

## Abstract

Doyle–Kirmse rearrangement reactions are widely recognized as versatile and powerful synthetic tools, promoting the simultaneous generation of C─C and C─S bonds. While notable progress has been attained, Doyle–Kirmse rearrangement reactions involving cyanocarbenes have rarely been reported owing to the limited strategies available for the preparation of cyanocarbene precursors and its inherently restricted reactivity. Herein, we report photochemical Doyle–Kirmse rearrangement reactions of alkynyl triazenes via intermediate cyanocarbenes under metal‐ and additive‐free conditions, leading to valuable α‐mercapto‐nitriles in generally moderate‐to‐good yields. In contrast to existing methods that utilize alternative cyanocarbene precurors, this approach delivers the possibility to access highly substituted stereogenic carbon centers and it offers a safer alternative by avoiding hazardous reagents. The reactions proceed under mild conditions, and demonstrate broad substrate compatibility. In addition, upscaling of the reaction was successfully demonstrated. Besides the use of allylsulfides, propargyl as well as allenylsulfides were also feasible substrates demonstrating the great synthetic potential of the transformation.

## Graphical abstract

A metal‐ and additive‐free photocatalytic Doyle–Kirmse reaction of alkynyl triazenes is described, proceeding via cyanocarbene intermediates. This strategy enables the efficient construction of highly substituted asymmetric carbon centers with broad substrate scope, delivering synthetically valuable nitriles.

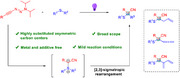

## Introduction

**Scheme 1:**
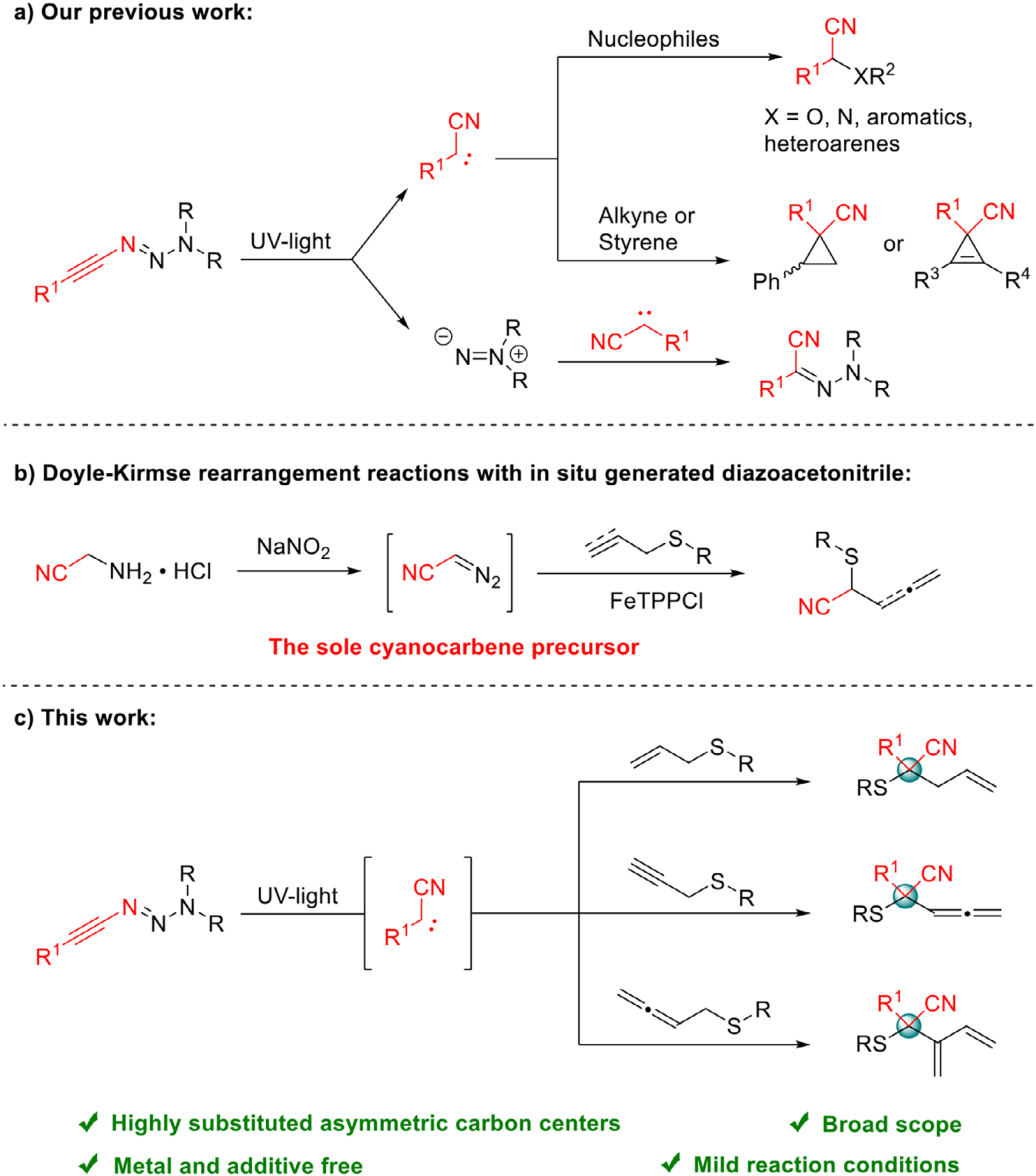
Transfer reactions of cyanocarbenes.

Alkynyl triazenes are a unique class of activated alkynes in which the electron‐donating triazene group is directly connected to the carbon–carbon triple bond. This results in triple bond polarization, which enables them to exhibit ynamide‐like reactivity,^[^ ^1^ ^]^ originally disclosed by Severin's group in 2015.^[^ ^2^ ^]^ Therefore, in the past few years, most reports about alkynyl triazenes were based on transition metal‐catalyzed 1,2‐additions,^[^ ^3^, ^4^, ^5^ ^]^ cycloadditions,^[^ ^6^, ^7^, ^8^ ^]^ and annulation reactions.^[^ ^9^, ^10^ ^]^ In addition, different transformations of alkynyl triazenes under acidic conditions were reported.^[^ ^1^, ^11^, ^12^ ^]^ In contrast, less attention was given to photochemical activity. In 2023, our group first revealed the photochemical reaction of alkynyl triazenes, which involved the cleavage of the triazene into a cyanocarbene and an isodiazene^[^ ^13^ ^]^ under UV irradiation. Downstream transformations of the intermediate carbene were enabled by appropriate nucleophiles (such as alcohols, amines, and electron‐rich aromatics) to give nitrile compounds, as well as the capture of the carbene by alkenes or alkynes to generate cyanocyclopropane or cyclopropene compounds. In addition, by recombination of the cyanocarbene and the isodiazene, a hydrazine derivative can be formed, which was regarded as a by‐product in this type of UV‐light reaction (Scheme 1a).^[^ ^14^ ^]^ Subsequently, our group further expanded the range of reactions of alkynyl triazenes with alkenes and alkynes, and accompanying mechanistic investigations revealed that the diastereoselectivity for cyclopropanations was influenced by the relative stability of singlet and triplet states of the applied cyanocarbene and the dispersion attraction between the carbene and olefin.^[^ ^15^ ^]^

To further explore the reactivity of cyanocarbenes formed from alkynyl triazenes, we turned our attention to the [2,3]‐sigmatropic rearrangement reactions of carbenes with sulfides, also known as the Doyle–Kirmse reaction,^[^ ^16^, ^17^ ^]^ which is a potent tool for the simultaneous construction of new C─C and C─S bonds. Typically, these reactions are conducted via metal carbenes, formed by the transition metal‐catalyzed decomposition of diazo compounds. Subsequent attack of the metal carbene by sulfides gives rise to sulfonium ylides, which then undergo [2,3]‐sigmatropic rearrangement.^[^ ^18^, ^19^, ^20^, ^21^, ^22^, ^23^, ^24^, ^25^, ^26^, ^27^, ^28^, ^29^, ^30^, ^31^ ^]^ In recent decades, asymmetric Doyle–Kirmse reactions have also received considerable attention.^[^ ^32^, ^33^, ^34^, ^35^, ^36^, ^37^ ^]^ With the rise of photochemical approaches,^[^ ^38^, ^39^, ^40^ ^]^ various carbene precursors, such as trifluoroacetylsilanes and diazo compounds, have been extensively investigated for light‐induced carbene generation under mild conditions.^[^ ^41^, ^42^, ^43^ ^]^ Compared with traditional metal‐mediated or thermal pathways, photochemical methods operate under milder conditions, often proceed without metal catalysts, and are generally more environmentally friendly. Despite these advantages, photochemical Doyle–Kirmse reactions are poorly explored with only a few reported examples. Recently, Xiao,^[^ ^44^ ^]^ König,^[^ ^45^, ^46^ ^]^ Gryko,^[^ ^47^ ^]^ and Wang^[^ ^48^ ^]^ groups have reported blue‐light induced reactions of diazo compounds with sulfides, and provided evidence that these reactions proceed via the free ylide mechanism.

However, either catalyzed by a transition metal or photocatalyzed, Doyle–Kirmse reactions which are based on intermediate cyanocarbenes are extremely rare. This can be rationalized by the limited existing strategies toward cyanocarbenes which comprise: 1) the formation of ethynyl azides through the reaction of hypervalent iodonium alkynyl triflates (HIATs) or (chloroethynyl)arenes with azides, followed by cleavage of the resulting ethynyl azides;^[^ ^49^, ^50^ ^]^ 2) through the decomposition of diazo compounds;^[^ ^51^, ^52^ ^]^ or 3) the synthesis of diazoacetonitrile from amino acetonitrile hydrochloride and sodium nitrite,^[^ ^53^, ^54^ ^]^ followed by transition metal‐mediated or myoglobin‐mediated cyanocarbene formation.^[^ ^55^, ^56^ ^]^ As a consequence, reactions of cyanocarbenes are limited and they are primarily utilized for cyclopropanation or cyclopropenylation reactions and interactions with nucleophiles or electron‐rich aromatic compounds.^[^ ^51^, ^52^, ^55^, ^56^, ^57^ ^]^ So far, only the König group has reported an example of an iron‐catalyzed Doyle–Kirmse rearrangement reaction in the presence of in situ generated diazoacetonitrile. While this strategy provides good yields, it relies on the synthesis of diazoacetonitrile, which requires careful handling due to its highly explosive nature.^[^ ^53^ ^]^ Moreover, the use of a single carbene precursor limits the flexibility for R‐group modification, thereby restricting the substrate scope to unsubstituted cyanocarbene systems (Scheme 1b).^[^ ^58^ ^]^

Different from our previous studies on the reactivity of cyanocarbenes derived from alkynyl triazenes and the limitations of conventional methods for cyanocarbenes, we herein report the Doyle–Kirmse reaction of alkynyl triazenes with sulfides under UV irradiation to obtain valuable nitrile compounds. This approach provides an effective and reliable strategy for the construction of highly substituted asymmetric carbon centers (Scheme 1c).

## Results and Discussion

Initially, alkynyl triazene **1a** and commercially available allyl (phenyl) sulfide **2a** were used as the model reaction under UV irradiation at room temperature (Table 1). To our satisfaction, the reaction proceeded smoothly in acetonitrile (MeCN), yielding the desired compound **3aa** (entry 1). Subsequently, we screened a range of solvents and observed that the reaction showed the best performance in dichloromethane (DCM), affording compound **3aa** with a 59% isolated yield (entry 4). A control experiment showed that the reaction did not proceed in the dark, which clearly demonstrates that UV‐light is indispensable for this transformation (entry 9). Furthermore, we conducted experiments at different concentrations to improve the yield of **3aa**. When we increased the concentration of alkynyl triazene **1a** from 0.05 to 0.10 M, the yield of **3aa** decreased and the proportion of hydrazone as a byproduct increased (mentioned in Scheme 1a) (entry 10). Reducing the concentration from 0.05 to 0.03 M resulted in no change in yield compared to the model reaction (entry 11). Considering the competition between intramolecular recombination (forming the hydrazine) and intermolecular reaction in this system, we further increased the amount of sulfide **2a** from the original 2.0 equiv. to 5.0 equiv., 10.0 equiv., and 20.0 equiv. In this series the yield of **3aa** gradually increased to 73% (entry 12–14). Finally, the conditions in entry 13 were selected as the optimal conditions for the transformation. For economic efficiency, sulfides were recycled after each use, as they are stable enough.

**Table 1:** Optimization of the reaction conditions.^a)^

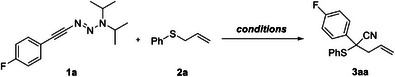				
Entry	Solvent	Conc. (M)	Ratio (1a:2a)	Yield (%)b)
1	MeCN	0.05	01:02	50
2	MeOH	0.05	01:02	trace
3	THF	0.05	01:02	43
4	DCM	0.05	01:02	59
5	DCE	0.05	01:02	46
6	Toluene	0.05	01:02	31
7	DMSO	0.05	01:02	ND
8	DMF	0.05	01:02	trace
9^c)^	DCM	0.05	01:02	NR
10^d)^	DCM	0.1	01:02	51
11^e)^	DCM	0.03	01:02	58
12	DCM	0.05	01:05	64
13	DCM	0.05	01:10	71
14	DCM	0.05	01:20	73

^a)^ All reactions were conducted on a 0.1 mmol scale with respect to **1a**, degassed solvent (2.0 mL), irradiated with UV light at room temperature for 16 h under nitrogen atmosphere.

^b)^ Isolated yield.

^c)^ The reaction was performed in the dark.

^d)^ DCM (1.0 mL).

^e)^ DCM (3.0 mL).

**Scheme 2:**
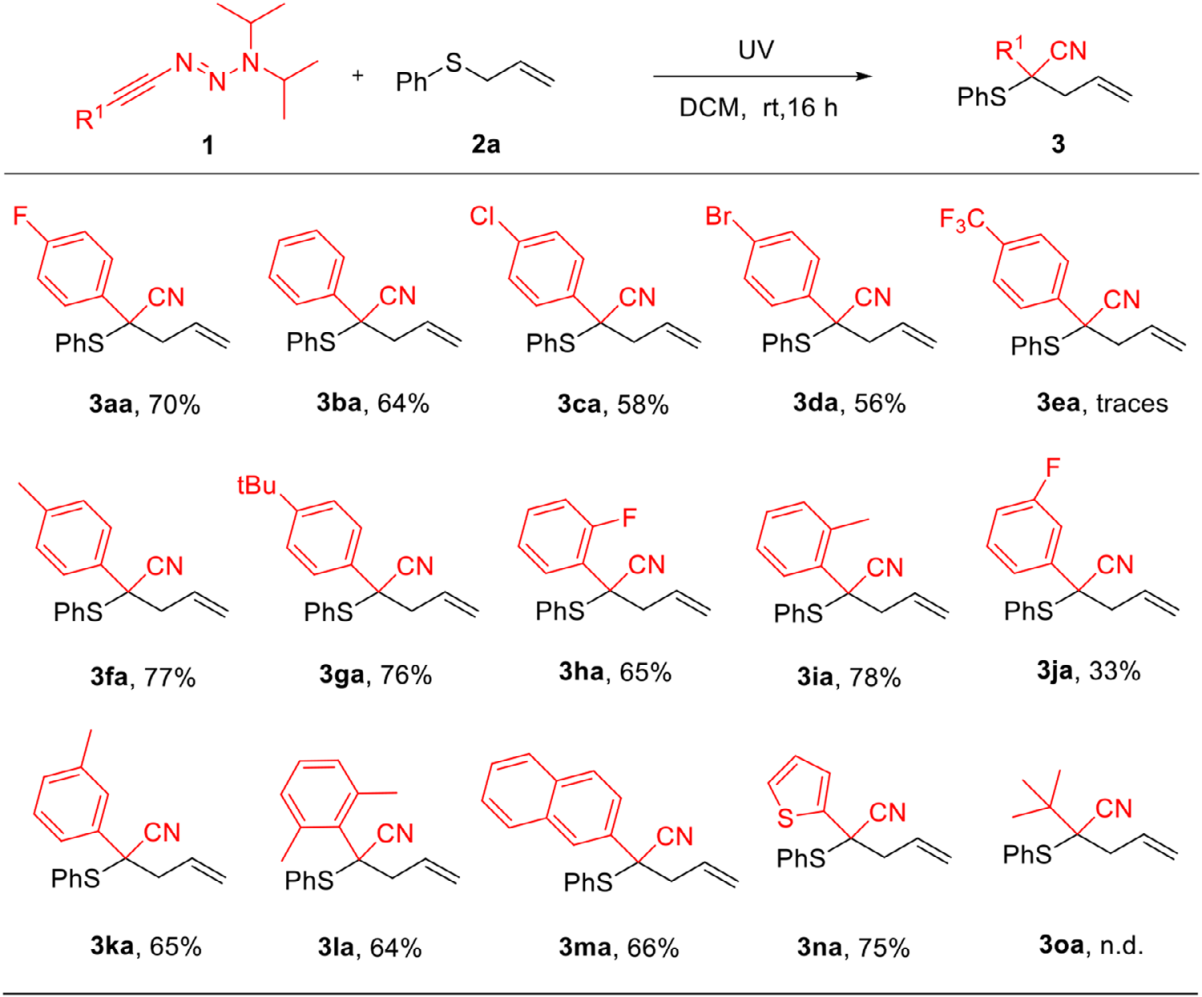
Scope of alkynyl triazenes. Reaction conditions: **1** (0.2 mmol), **2a** (10.0 equiv.), degassed DCM (4.0 mL), rt, N_2_, irradiated with UV light for 16 h; isolated yield.

After establishing the optimal reaction conditions, we started to explore the substrate scope of this reaction. First, a range of alkynyl triazenes was examined (Scheme 2). The reactions of **2a** with alkynyl triazenes bearing electron‐donating and electron‐withdrawing groups at different positions of the aryl ring proceeded smoothly, affording the corresponding products in moderate‐to‐good yield (**3aa–3ka**, 33%–78%). Compared to alkynyl triazenes bearing electron‐withdrawing groups on the aryl ring, those with electron‐donating groups afforded higher yields. Unfortunately, for an alkynyl triazene bearing a strong electron‐withdrawing group (4‐CF_3_) on the aryl ring, only trace amounts of the desired product **3ea** were obtained. To our surprise, a sterically shielded ortho, ortho‐dimethyl‐substituted phenyl ring at the alkynyl triazene also gave a respectable yield (**3la**, 64%). In addition, the reaction was efficient for alkynyl triazenes bearing a naphthyl group and a thienyl group, affording products **3ma** and **3na** in 66% and 75% yield, respectively. When the phenyl group was replaced by a tert‐butyl group, we did not detect the target compound **3oa**, due to the decomposition of the starting material under UV irradiation.

**Scheme 3:**
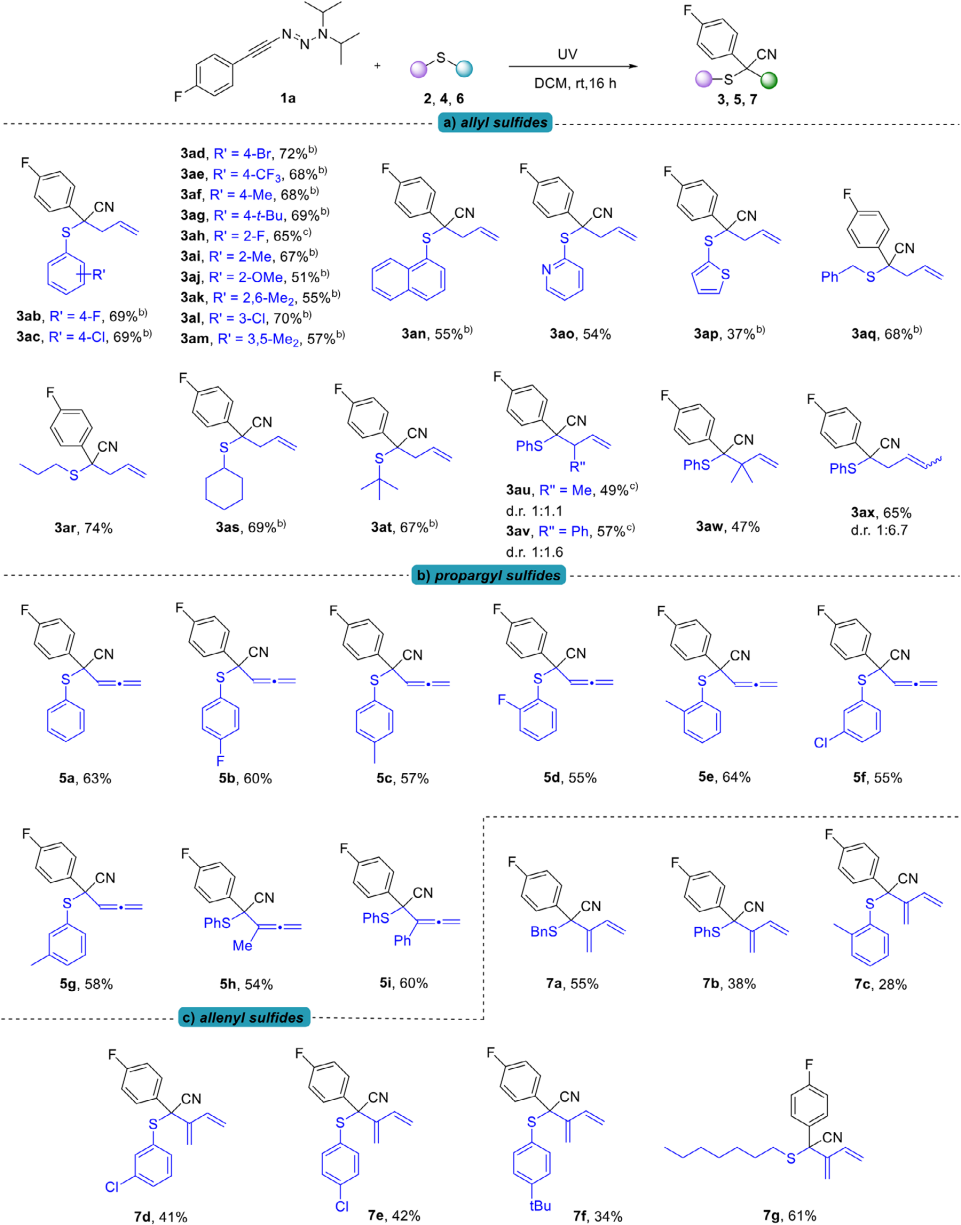
Scope with respect to the sulfides. ^a)^ Reaction conditions: **1a** (0.2 mmol), **sulfides** (10.0 equiv), degassed DCM (4.0 mL), rt, N_2_, irradiated with UV light for 16 h; isolated yield; the d.r. values were determined by ^1^H NMR analysis. ^b)^ **1a** (0.3 mmol), **sulfides** (10.0 equiv), DCM (6.0 mL). ^c)^ **1a** (0.1 mmol), **sulfides** (10.0 equiv), DCM (2.0 mL).

We then explored the scope of allyl sulfides (Scheme 3a). The reaction exhibited excellent tolerance toward both electron‐withdrawing and electron‐donating groups on the arylsulfides, and the corresponding products **3ab‐3am** were obtained in good yields ranging from 51% to 72%. When the phenyl ring on the sulfide either bore a strong electron‐donating group or more than one electron‐donating group, the yield of product (**3aj**, **3ak**, and **3am**) dropped slightly, affording the products in 51%, 55%, and 57% yield, respectively. In addition, allyl sulfides with an α‐naphthyl group, a 2‐pyridyl group and a 2‐thienyl group could be converted (**3an‐3ap**). As a next step, replacement of the aryl group with a benzyl group also gave **3aq** successfully. In particular, allyl sulfides carrying alkyl groups showed good results (**3ar**–**3at**, 67%–74%), despite the presence of bulky, sterically hindered substituents on the sulfur atom (**3as**, **3at**). Subsequently, different substitution patterns of allyl groups were also examined, including unbranched crotyl‐, cinnamyl‐ and dimethylallyl‐substituted sulfides, as well as branched crotyl‐substituted sulfides. All of which readily provided the desired products (**3au**‐**3ax**). For the obtained diastereomers of **3au** and **3av**, diastereomeric ratios comparable to the results observed in metal‐catalyzed reactions were achieved.^[^ ^20^, ^23^, ^35^, ^58^ ^]^ Additionally, compound **3ax** was isolated in 65% yield with a diastereomeric ratio (dr) of 1:6.7.

In order to broaden the scope of this transformation, we further assessed the reactivity of different propargyl sulfides (Scheme 3b). The initially attempted transformation using the non‐substituted terminal alkyne propargyl sulfide and **1a** as model system successfully delivered the addressed allene derivative **5a** in a yield of 63%. Based on this result, we then proceeded to introduce different substituents to the phenyl group of propargyl sulfides, both electron‐withdrawing and electron‐donating groups were tolerated all delivering similar acceptable yields (**5b–5g**, 55%–64%). The x‐ray crystallographic data of compound **5d** was also obtained which clearly underlines the correct assignment (see Supporting Information).^[^ ^59^ ^]^ Notably, propargyl sulfides in which the terminal alkyne was substituted with either a methyl or a phenyl group gave satisfactory yields, specifically 54% for **5h** and 60% for **5i**.

Encouraged by the above results, we were increasingly motivated to investigate the substrate scope of allenyl sulfides as well (Scheme 3c). According to the literature,^[^ ^60^ ^]^ we successfully synthesized allenyl sulfide **6a** and subsequently attempted its transformation under standard conditions. To our delight, the reaction effectively yielded the desired 1,3‐diene compound **7a**, a transformation which has not been previously reported in any Doyle–Kirmse reaction. To better investigate its reactivity, the benzyl group was replaced by a phenyl group, however, this modification led to clear reductions in yield (**7b–7f**). Due to the instability of these compounds, decomposition occurred during prolonged UV irradiation and in the separation process, which explains the lower yield. Lastly, the aryl ring was substituted with an alkyl group, which delivered a favorable yield (**7g**, 61%).

**Scheme 4:**
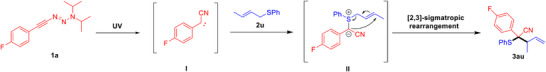
Plausible mechanism.

With reference to recent reports on the blue‐light induced [2,3]‐sigmatropic rearrangement of diazo compounds and sulfides,^[^ ^44^, ^45^, ^46^, ^47^, ^48^ ^]^ we speculated that the reaction between alkynyl triazenes and sulfides under UV irradiation could be mediated by a [2,3]‐sigmatropic rearrangement process (Scheme 4). As an example, alkynyl triazene **1a** and methylallylsulfide **2u** were used to illustrate the reaction mechanism. Under UV irradiation, alkynyl triazene **1a** first generates cyanocarbene intermediate **I**, which subsequently reacts with sulfide **2u** to afford sulfonium ylide intermediate **II**. Ultimately, intermediate **II** undergoes a [2,3]‐sigmatropic rearrangement, leading to the formation of compound **3au**. However, considering the key intermediate **II**, we questioned whether compound **3au** was formed by the formation of the allyl cation in intermediate **II**, followed by nucleophilic attack by the carbanion to form the product.

**Scheme 5:**
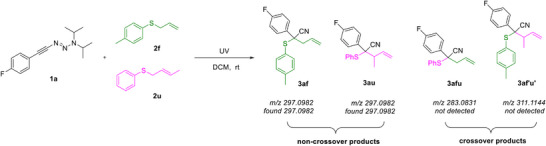
Crossover experiment. Crossover experiment was carried out using **1a** (0.1 mmol), **2f** (10.0 equiv.) and **2u** (10.0 equiv.) in DCM (2.0 mL) at room temperature under UV irradiation for 16 h under nitrogen atmosphere.

**Figure 1:**
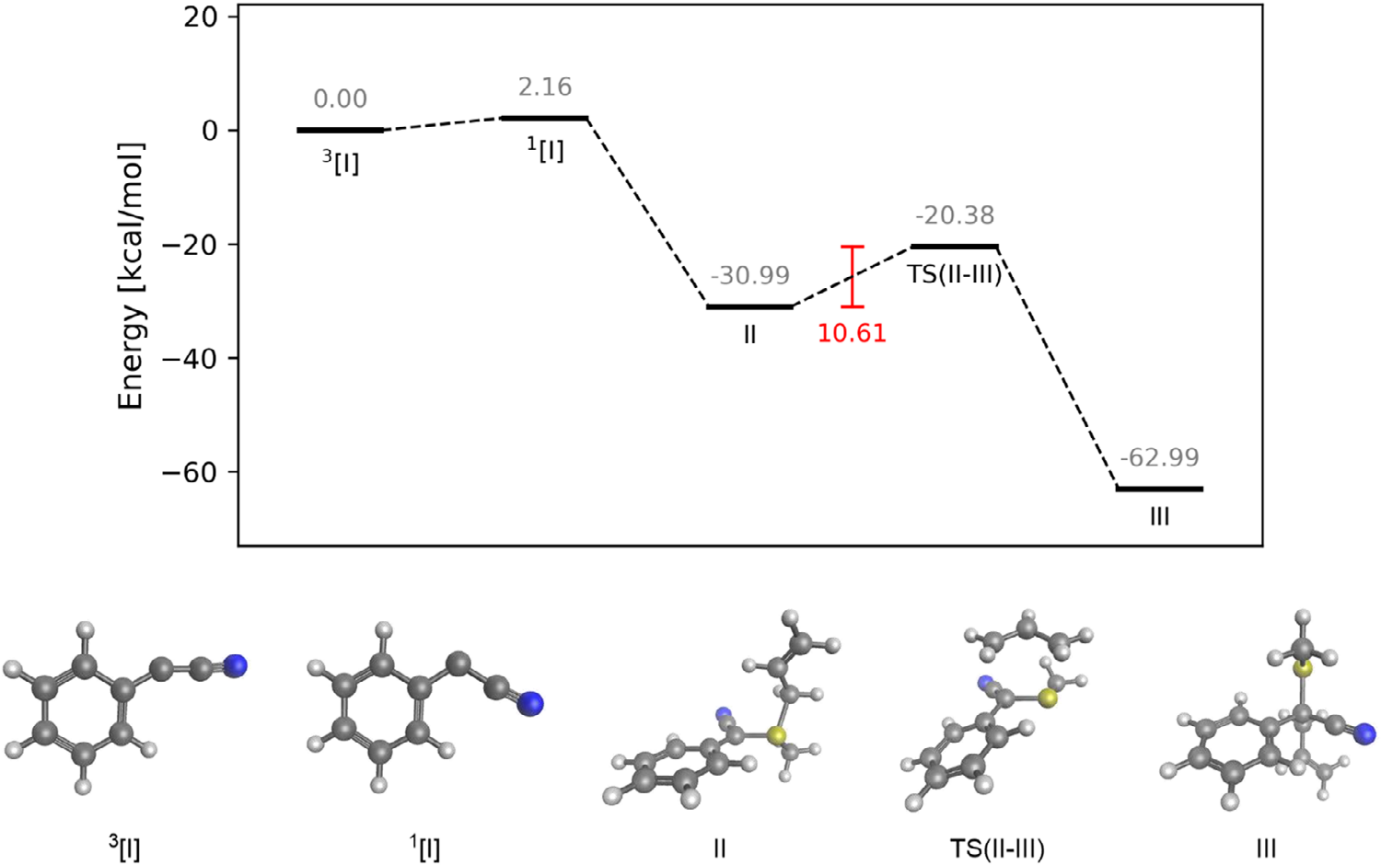
Top: Energetic profile of the proposed reaction mechanism. All energies are given in kcal mol^−1^ and were evaluated using ωB97M(2)/def2‐QZVPPD/CPCM with DCM as a solvent plus a zero‐point vibrational energy correction at the ωB97M‐D4/def2‐QZVPPD level of theory. Bottom: Three‐dimensional structures of the intermediates and transition states in the proposed reaction mechanism.

Motivated by the need to clarify the reaction mechanism, we conducted a crossover experiment (Scheme 5). Alkynyl triazene **1a** and a 1:1 mixture of allyl sulfide **2f** and **2u** was subjected to the standard reaction condition. The results showed that only the non‐crossover products **3af** and **3au** were formed (analyzed by crude ^19^F NMR spectra and high‐resolution mass spectrometry; the crossover product **3afu**, **3af'u’** were not detected). This result indicates that the product was not formed through the formation of a free allyl cation after the formation of the sulfonium ylide. To further elucidate the proposed reaction mechanism, we utilized quantum chemical calculations (computational details are given in the Supporting Information). We considered a model reaction of phenyl triazene with methylallylsulfide, whose reaction energy profile is given in Figure 1. Since the generation of cyanocarbenes from triazenes via UV irradiation has been explored in detail in our earlier work,^[^ ^15^ ^]^ we start our mechanistic discussion with the free carbene. As previously established, a key determinant for the reactivity of cyanocarbenes is the relative stability of their singlet and triplet spin states. Generally, carbenes in solution rapidly interconvert between spin states, wherefore the reaction from singlet to triplet carbene can be assumed to be in thermodynamic equilibrium at any given time. For this transformation, we found that only the singlet state of the carbene produces structure **II**, while to our surprise, this adduct does not have a stable triplet state. This implies that the reaction can only proceed via the singlet carbene. Notably, this addition is barrier free, wherefore its kinetics are entirely determined by the diffusion of the reactants and the equilibrium constant of singlet‐triplet interconversion. Our calculations confirmed that the next step in the reaction is indeed a [2,3]‐sigmatropic rearrangement with a barrier of 10.61 kcal/mol, which directly generates the product. Note that this reaction barrier corresponds to a half‐life in the microsecond regime. The rate determining step is therefore the photoinduced formation of the cyanocarbene, after which the rest of the reaction proceeds swiftly.

**Scheme 6:**
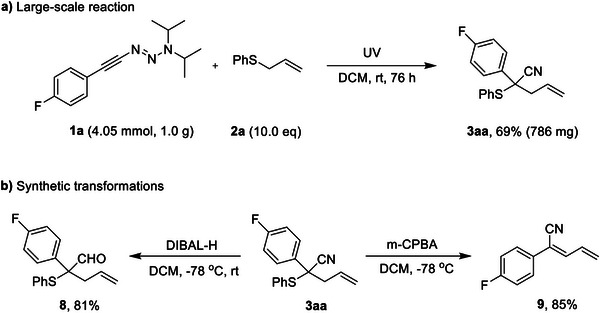
Scale‐up reaction and synthetic transformations of product **3aa**.

To further evaluate the practical applicability of the present approach, a large‐scale reaction was performed on a 4.05 mmol scale, affording the compound **3aa** in good yield (Scheme 6a). In addition, transformations involving compound **3aa** were undertaken. For instance, in the presence of DIBAL‐H, compound **3aa** was easily converted into the corresponding aldehyde derivative **8** with a yield of 81%.^[^ ^61^ ^]^ Moreover, under oxidative conditions with *m*‐chloroperbenzoic acid (*m*‐CPBA), elimination of the intermediate sulfur oxide enabled double bond formation, affording the unsaturated nitrile **9** in 85% yield (Scheme 6b).^[^ ^62^ ^]^

## Conclusion

In conclusion, we have developed a photocatalytic synthetic strategy for the efficient generation of valuable α‐mercapto‐nitriles. This transformation employed the Doyle–Kirmse reaction of alkynyl triazenes with allyl, propargyl or allenyl sulfides. The protocol is remarkably simple, it can be carried out under metal‐free conditions without the need for additives using a simple and convenient operation. The method can be applied for a wide range of substrates, showcasing a high tolerance to various functional groups, which overcomes the limitation of the range of diazoacetonitrile as a single substrate for the reaction of cyanocarbene precursor with sulfides. A crossover experiment accompanied by theoretical analysis provided evidence for the mechanism that is characterized by a [2,3]‐sigmatropic rearrangement initiated by the attack of the sulfide onto the singlet cyanocarbene. The reaction could be scaled up and initial examples for converting the obtained α‐mercapto‐nitriles into follow‐up products demonstrate the potential of this methodology. Overall, this method offers a unique tool to apply cyanocarbenes as intermediates in Doyle–Kirmse reaction to enable the construction of synthetically desirable highly substituted asymmetric carbon centers.

## Supporting Information

The authors have cited additional references within the Supporting Information.

## Conflict of Interests

The authors declare no conflict of interest.

## Supporting information



Supporting Information

Supporting Information

## Data Availability

The data that support the findings of this study are available in the Supporting Information of this article.
