# Supplementary material for: Alkynyltriazenes in Photochemical Metal‐Free Doyle–Kirmse Rearrangements
Source: Angew Chem Int Ed Engl. 2026 Jan 10;65(7):e21012. doi: 10.1002/anie.202521012 (PMC12887635; doi:10.1002/anie.202521012)
Supplement: Supplementary file 1 — Supporting Information [file ANIE-65-e21012-s001.docx]

Supporting Information

**Alkynyltriazenes in Photochemical Metal-Free Doyle-Kirmse Rearrangements**

Ningning Liu,^[a]^ Linus Bjarne Dittmer,^[b]^ Melina Maag,^[a]^ Elena Michel,^[a]^ Frank Rominger,^[a]^ Matthias Rudolph,^[a]^ Andreas Dreuw^[b]^ and A. Stephen K. Hashmi*^[a,c]^

^[a]^Organisch-Chemisches Institut, Heidelberg University, Im Neuenheimer Feld 270, 69120 Heidelberg, Germany.

^[b]^Interdisziplinäres Zentrum für Wissenschaftliches Rechnen (IWR), Heidelberg University, Im Neuenheimer Feld 205, 60120 Heidelberg, Germany.

^[c]^Chemistry Department, Faculty of Science, King Abdulaziz University, Jeddah 21589, Saudi Arabia

E-mail: hashmi@hashmi.de

**Table of content**

[1. General Information 1](#_Toc213091434)

[2. General Procedures 2](#_Toc213091435)

[3. Characterization Data of the Compounds 4](#_Toc213091436)

[4. Mechanistic Experiments 32](#_Toc213091437)

[5. Computational Details 35](#_Toc213091438)

[6. References 43](#_Toc213091439)

[7. NMR Spectra 44](#_Toc213091440)

[8. X-ray Crystal Structure 125](#_Toc213091441)

# 1. General Information

Chemicals were purchased from commercial suppliers (abcr, Acros, Alfa Aesar, BLDPharm, Carbolution, Chempur, Fluka, Merck, Sigma Aldrich and TCI) and used as delivered. Anhydrous solvents were dispensed from a solvent purification system MB SPS-800. Solvents were degassed by freeze-pump-thaw-technique. Deuterated solvents were bought from Eurisotop and Sigma Aldrich.

Nuclear magnetic resonance (NMR) spectra, if not noted otherwise, were recorded at room temperature on the following spectrometers: Bruker Avance III 300 (300 MHz), Bruker Avance DRX 300 (300 MHz), Bruker Avance II 400 (400 MHz), Bruker Avance III 400 (400 MHz), Bruker Avance III 500 (500 MHz), Bruker Avance III 600 (600 MHz) or Fourier 300 (300 MHz). Chemical shifts δ are quoted in parts per million (ppm) and coupling constants *J* in hertz (Hz). ^1^H and ^13^C NMR spectra are calibrated in relation to the deuterated solvents. The following abbreviations were used to indicate the signal multiplicity: for the ^1^H NMR spectra: s (singlet), d (doublet), t (triplet), q (quartet), quint (quintet), sext (sextet), sept (septet), m (multiplet), as well as their combinations; for the ^13^C NMR spectra: s (quaternary carbon), d (tertiary carbon (CH)), t (secondary carbon (CH_2_)) and q (primary carbon (CH_3_)). All spectra were integrated and processed using MestreNova software.

Mass spectra (MS and HRMS) were determined in the chemistry department of the University of Heidelberg under the direction of Dr. J. Gross. Spectra were measured on a JOEL AccuTOF GCx spectrometer, Bruker timsTOFfleX and Bruker Autoflex speed MALDI-TOF Instruments.

Gas chromatography / Mass Spectrometry (GC MS) was carried out on an Agilent 5975C Mass Selective Detector, coupled with an Agilent 7890A Gas Chromatograph. As a capillary column an OPTIMA 5 cross-linked Methyl Silicone column (30 Mesh: 0.25 mm, 0.25 μm) was employed, and nitrogen was used as the carrier gas.

Flash Column Chromatography was accomplished using Silica gel 60 (0.04 – 0.063 mm / 230 – 400 mesh ASTM) purchased from Macherey-Nagel as stationary phase. As eluents the respectively mentioned proportions of petroleum ether (PE), ethyl acetate (EA), diethyl ether (Et_2_O), toluene, and triethylamine (NEt_3_) were used. Analytical Thin Layer Chromatography (TLC) was carried out on precoated Macherey-Nagel POLYGRAM®SIL G/UV254 or Merck TLC Silical Gel 60 F254 aluminium sheets. Detection was accomplished using UV-light (254 and 366 nm).

Infrared (IR) spectra were measured using a Bruker Lumos, Germanium ATR-Crystal spectrometer.

X-ray structures were measured by a Stoe Stadivari or a Bruker Smart APEX II Quazar instrument and processed using Mercury 3.10.3 software.

The UV-photoreactor was equipped with a fan and a magnetic stirring machine at the bottom to keep the reaction at ambient temperature during the reaction processes. The reaction tubes were placed in a sample holder about 8 cm in front of the UVA lamps (8*24 W black light lamps). The UVA lamps (340 - 400 nm, rayonet PRP-3500 Å) were purchased from Southern New England Ultraviolet Company.

# 2. General Procedures

**General procedure for synthesis of Alkynyl Triazenes 1. (GP1)**

According to the literature,^[1]^ under nitrogen atmosphere, the alkyne (16.7 mmol, 1.50 eq.) was dissolved in 20 mL dry THF and 5.55 mL of ethyl magnesium bromide (3.00 M in Et_2_O, 16.7 mmol, 1.50 eq.) was added dropwise. The reaction mixture was then heated to 50 °C for 4 hours.

Parallel, a dried Schlenk flask with 20 mL of dry THF was charged with N_2_O atmosphere and 5.55 mL of LDA (2 M in THF, 11.1 mmol, 1.00 eq.) was added. The reaction mixture was stirred at room temperature for 3 hours and a colorless precipitate formed. Afterwards, the N_2_O atmosphere was replaced by nitrogen gas and the alkyl magnesium bromide solution in THF/Et_2_O added. The combined reaction mixture was stirred overnight at 50 °C. Then, 10 mL water were added, and the reaction filtered over a Celite plug with ethyl acetate. After evaporation of the solvent, the crude product was purified via column chromatography on deactivated silica (PE with 3% triethylamine as deactivator) to give **1**.

Comprehensive data on alkynyl triazenes **1** have been reported in the literature.^[1]^

**General procedure for synthesis of** **sulfide 2. (GP2)**

According to the literature,^[2]^ NaOH (400 mg, 10.0 mmol, 1.00 eq.) was added to a 100 mL round bottom flask and dissolved in 13 mL of deionized H_2_O. The stirred solution was cooled to 0 ˚C and thiol (10.0 mmol, 1.00 eq.) was added. After 10 minutes of stirring, allyl chloride (0.90 mL, 11.0 mol, 1.10 eq.) was added. The resulting cloudy reaction mixture was stirred vigorously for 10 hours at room temperature. TLC (10% EtOAc in hexane) indicated the formation of a single product. The reaction was diluted with 130 mL Et_2_O and washed successively with 0.1 M NaOH (3 × 50 mL) and brine (50 mL). The organic layer was dried over MgSO_4_, filtered, and concentrated under reduced pressure to give **2** as a clear oil that required no purification.^[3-8]^

Sulfides **2a** and **2r** are commercially available.

**General procedure for synthesis of** **sulfide 4.**

The synthesis was carried out according to **GP2**, NaOH (400 mg, 10.0 mmol, 1.00 eq.) was added to a 100 mL round bottom flask and dissolved in 13 mL of deionized H_2_O. The stirred solution was cooled to 0 ˚C and thiol (10.0 mmol, 1.00 eq.) was added. After 10 minutes of stirring, propargyl chloride (0.80 mL, 11.0 mol, 1.10 eq.) was added. The resulting cloudy reaction mixture was stirred vigorously for 10 hours at room temperature. TLC (10% EtOAc in hexane) indicated the formation of a single product. The reaction was diluted with 130 mL Et_2_O and washed successively with 0.1 M NaOH (3 × 50 mL) and brine (50 mL). The organic layer was dried over MgSO_4_, filtered, and concentrated under reduced pressure to give **4** as a yellow oil that required no purification.^[4, 9-10]^

According to the literature,^[11]^ to a solution of thiophenol (0.60 mL, 6.00 mmol, 1.00 eq.) in anhydrous DMF (5.50 mL), K_2_CO_3_ (1.66 g, 12.0 mmol, 2.00 eq.) was added. The reaction mixture was stirred for 30 min at room temperature under argon atmosphere. Next 1-bromobut-2-yne (0.63 mL, 7.20 mmol, 1.20 eq.) was added dropwise and stirred. After 12 hours reaction mixture was quenched with water (10 mL) and extracted with diethyl ether (3 × 10 mL). The organic layers were combined, washed with saturated brine solution (15 mL), dried over anhydrous MgSO_4_, and concentrated under reduced pressure. The crude product was purified by column chromatography using petroleum ether as mobile phase to afford but-2-yn-1-yl(phenyl)sulfane **4h** as a yellow oil (810 mg, 83%).

Step 1: According to the literature,^[11]^ to a solution of 3-phenylprop-2-yn-1-ol (1.25 mL, 10.0 mmol, 1.00 eq.) in anhydrous dichloromethane (40 mL), triethylamine (2.10 mL, 15.0 mmol, 1.50 eq.) was added at 0 ºC. Next, methanesulfonyl chloride (1.00 mL, 12.5 mmol, 1.25 eq.) was added dropwise and stirred for 1 hour. To this reaction mixture, 10% aqueous HCl solution (30 mL) was added and extracted with ethyl acetate (3 × 30 mL). The organic layers were combined, dried over anhydrous MgSO_4_, and concentrated under reduced pressure. The crude mesylated alcohol was used in the next step without further purification.

Step 2: According to the literature,^[11]^ thiophenol (2.00 mL, 20.0 mmol, 2.00 eq.) and NaOH (800 mg, 20.0 mmol, 2.00 eq.) were dissolved in water (50 mL). A solution of mesylated alcohol in THF (12.5 mL) was added dropwise at room temperature and stirred. After 3 hours, the reaction mixture was quenched with 10% aqueous NaOH (30 mL) and extracted with diethyl ether (3 × 50 mL). The organic layers were combined, washed with water (50 mL), dried over anhydrous MgSO_4_, and concentrated under reduced pressure. The crude product was purified by column chromatography using petroleum ether as mobile phase to afford the desired phenyl(3-phenylprop-2-yn-1-yl)sulfane **4i** as a yellow oil (1.15 g, 51%).

**General procedure for synthesis of** **allenes 6. (GP3)**

According to the literature,^[12]^ to a 50 mL three-neck round-bottom flask equipped with a magnetic stirring bar were added CuI (191 mg, 1.00 mmol, 0.10 eq.), paraformaldehyde (481 mg, 16.0 mmol, 1.60 eq.), and dioxane (10 mL). The resulting mixture was stirred at room temperature before subsequent addition of *i*Pr_2_NH (2.00 mL, 14.0 mmol, 1.40 eq.), propargyl sulfide (10.0 mmol, 1.00 eq.), and dioxane (5 mL). The mixture was stirred at 110 °C without protection with an inert atmosphere. After 16 h, the reaction mixture was cooled to room temperature and filtered through a short column of silica gel (Et_2_O). The solvent was evaporated, and the residue was diluted with Et_2_O. After filtration again to remove the precipitate, evaporation of the solvent and chromatography on silica gel (petroleum ether) afforded **6** as a liquid.

**General procedure for synthesis of** **Compound 3, 5, 7. (GP4)**

Triazene (0.10 - 0.30 mmol, 1.00 eq.) and sulfide (1.00 - 3.00 mmol, 10.0 eq.) were added in a pyrex-screw tube. Then under the protection of N_2_, anhydrous and degassed dichloromethane (2 - 6 mL) was added. The reaction mixture was stirred under UV light at room temperature for 16 hours.

**Experimental procedure for the large-scale reaction**

Under nitrogen atmosphere, alkynyl triazene **1a** (4.05 mmol, 1.00 g) was added in two portions over 9 hours to 40 mL of degassed DCM containing 6.08 g of allyl (phenyl) sulfide **2a**. After the final portion was added, the reaction was stirred for an additional 67 hours. After the reaction was complete, the DCM was removed under reduced pressure, and the crude product was purified by column chromatography, first eluting with petroleum ether, followed by petroleum ether/ethyl ether (75:1) as the eluent to give the compound **3aa** as a yellow oil (786 mg, 69%). Additionally, 5.43 g of **2a** was recovered after the irradiation time was prolonged to 76 h.

# 3. Characterization Data of the Compounds

**Buta-2,3-dien-1-yl(phenyl)sulfane (6b)**

The reaction was carried out according to **GP3**, using **4a** (1.48 g, 10.1 mmol, 1.00 eq.), paraformaldehyde (487 mg, 16.2 mmol, 1.60 eq.), CuI (193 mg, 1.00 mmol, 0.10 eq.), and *i*Pr_2_NH (2.00 mL, 14.2 mmol, 1.40 eq.). Purification by flash column chromatography gave the product as a yellow oil (918 mg, 56%).

**R_f_** (SiO_2_, PE): 0.20; **^1^H NMR** (400 MHz, CDCl_3_): δ = 7.35-7.38 (m, 2H), 7.27-7.31 (m, 2H), 7.19-7.22 (m, 1H), 5.18-5.25 (m, 1H), 4.71 (dt, *J* = 6.7, 2.5 Hz, 2H), 3.54 (dt, *J* = 7.6, 2.5 Hz, 2H) ppm.

Data is consistent with literature values.^[13]^

**Buta-2,3-dien-1-yl(o-tolyl)sulfane (6c)**

The reaction was carried out according to **GP3**, using **4e** (1.62 g, 10.0 mmol, 1.00 eq.), paraformaldehyde (481 mg, 16.0 mmol, 1.60 eq.), CuI (191 mg, 1.00 mmol, 0.10 eq.), and *i*Pr_2_NH (2.00 mL, 14.0 mmol, 1.40 eq.). Purification by flash column chromatography gave the product as a yellow oil (783 mg, 44%).

**R_f_** (SiO_2_, PE): 0.20; **^1^H NMR** (400 MHz, CDCl_3_): δ = 7.30-7.32 (m, 1H), 7.10-7.19 (m, 3H), 5.18-5.25 (m, 1H), 4.72 (dt, *J* = 6.6, 2.5 Hz, 2H), 3.52 (dt, *J* = 7.6, 2.5 Hz, 2H), 2.39 (s, 3H) ppm. **^13^C NMR** (101 MHz, CDCl_3_): δ = 209.70 (s, 1C), 138.22 (s, 1C), 135.12 (s, 1C), 130.22 (d, 1C), 129.24 (d, 1C), 126.37 (d, 1C), 126.21 (d, 1C), 87.31 (d, 1C), 76.34 (t, 1C), 32.47 (t, 1C), 20.56 (q, 1C) ppm. **IR** (Reflexion)**:** ṽ = 3059, 3010, 2974, 2919, 2853, 1949, 1695, 1679, 1589, 1553, 1468, 1436, 1379, 1275, 1225, 1161, 1064, 1047, 985, 848, 744, 710, 677 cm^-1^. **HR-MS** (EI(+)): *m/z* = 176.0664, calcd. for [M]^+^ = [C_11_H_12_S]^+^: 176.0654.

**Buta-2,3-dien-1-yl(3-chlorophenyl)sulfane (6d)**

The reaction was carried out according to **GP3**, using **4f** (1.19 g, 6.50 mmol, 1.00 eq.), paraformaldehyde (312 mg, 10.4 mmol, 1.60 eq.), CuI (124 mg, 0.65 mmol, 0.10 eq.), and *i*Pr_2_NH (1.30 mL, 9.10 mmol, 1.40 eq.). Purification by flash column chromatography gave the product as a yellow oil (725 mg, 57%).

**R_f_** (SiO_2_, PE): 0.18; **^1^H NMR** (700 MHz, CDCl_3_): δ = 7.33-7.34 (m, 1H), 7.19-7.22 (m, 2H), 7.15-7.18 (m, 1H), 5.18-5.22 (m, 1H), 4.76 (dt, *J* = 6.6, 2.5 Hz, 2H), 3.54 (dt, *J* = 7.6, 2.5 Hz, 2H) ppm. **^13^C NMR** (176 MHz, CDCl_3_): δ = 209.92 (s, 1C), 138.08 (s, 1C), 134.66 (s, 1C), 129.86 (d, 1C), 129.20 (d, 1C), 127.69 (d, 1C), 126.34 (d, 1C), 87.16 (d, 1C), 76.70 (t, 1C), 32.92 (t, 1C) ppm. **IR** (Reflexion)**:** ṽ = 3058, 2920, 1949, 1577, 1563, 1461, 1406, 1322, 1225, 1163, 1119, 1086, 994, 849, 776, 678 cm^-1^. **HR-MS** (EI(+)): *m/z* = 196.0108, calcd. for [M]^+^ = [C_10_H_9_ClS]^+^: 196.0108.

**Buta-2,3-dien-1-yl(4-chlorophenyl)sulfane (6e)**

The reaction was carried out according to **GP3**, using (4-chlorophenyl)(prop-2-yn-1-yl)sulfane (1.49 g, 8.15 mmol, 1.00 eq.), paraformaldehyde (392 mg, 13.0 mmol, 1.60 eq.), CuI (155 mg, 0.82 mmol, 0.10 eq.), and *i*Pr_2_NH (1.60 mL, 11.4 mmol, 1.40 eq.). Purification by flash column chromatography gave the product as a yellow oil (824 mg, 52%).

**R_f_** (SiO_2_, PE): 0.18; **^1^H NMR** (400 MHz, CDCl_3_): δ = 7.27-7.30 (m, 2H), 7.24-7.27 (m, 2H), 5.15-5.22 (m, 1H), 4.72 (dt, *J* = 6.6, 2.5 Hz, 2H), 3.50 (dt, *J* = 7.5, 2.5 Hz, 2H) ppm. **^13^C NMR** (101 MHz, CDCl_3_): δ = 209.81 (s, 1C), 134.30 (s, 1C), 132.60 (s, 1C), 131.71 (d, 2C), 129.04 (d, 2C), 87.33 (d, 1C), 76.53 (t, 1C), 33.61 (t, 1C) ppm. **IR** (Reflexion)**:** ṽ = 3061, 2922, 1949, 1682, 1573, 1557, 1477, 1436, 1418, 1389, 1225, 1095, 1012, 851, 816, 745 cm^-1^. **HR-MS** (EI(+)): *m/z* = 195.0070, calcd. for [M-H]^+^ = [C_10_H_8_ClS]^+^: 195.0040.

**Buta-2,3-dien-1-yl(4-(tert-butyl)phenyl)sulfane (6f)**

The reaction was carried out according to **GP3**, using (4-(tert-butyl)phenyl)(prop-2-yn-1-yl)sulfane (2.00 g, 10.0 mmol, 1.00 eq.), paraformaldehyde (481 mg, 16.0 mmol, 1.60 eq.), CuI (191 mg, 1.00 mmol, 0.10 eq.), and *i*Pr_2_NH (2.00 mL, 14.0 mmol, 1.40 eq.). Purification by flash column chromatography gave the product as a yellow oil (1.16 g, 53%).

**R_f_** (SiO_2_, PE): 0.20; **^1^H NMR** (400 MHz, CDCl_3_): δ = 7.31 (s, 4H), 5.17-5.25 (m, 1H), 4.67-4.70 (m, 2H), 3.49-3.52 (m, 2H), 1.31 (s, 9H) ppm. **^13^C NMR** (101 MHz, CDCl_3_): δ = 209.54 (s, 1C), 149.81 (s, 1C), 132.02 (s, 1C), 130.40 (d, 2C), 125.86 (d, 2C), 87.53 (d, 1C), 76.09 (t, 1C), 34.50 (s, 1C), 33.71 (t, 1C), 31.32 (q, 3C) ppm. **IR** (Reflexion)**:** ṽ = 3077, 2963, 2903, 2868, 1950, 1680, 1556, 1489, 1461, 1398, 1363, 1269, 1224, 1202, 1120, 1086, 1013, 849, 825, 778, 743, 724 cm^-1^. **HR-MS** (EI(+)): *m/z* = 218.1120, calcd. for [M]^+^ = [C_14_H_18_S]^+^: 218.1124.

**Buta-2,3-dien-1-yl(heptyl)sulfane (6g)**

The reaction was carried out according to **GP3**, using heptyl(prop-2-yn-1-yl)sulfane (1.70 g, 10.0 mmol, 1.00 eq.), paraformaldehyde (481 mg, 16.0 mmol, 1.60 eq.), CuI (191 mg, 1.00 mmol, 0.10 eq.), and *i*Pr_2_NH (2.00 mL, 14.0 mmol, 1.40 eq.). Purification by flash column chromatography gave the product as a yellow oil (945 mg, 51%).

**R_f_** (SiO_2_, PE): 0.25; **^1^H NMR** (400 MHz, CDCl_3_): δ = 5.12-5.19 (m, 1H), 4.78 (dt, *J* = 6.5, 2.4 Hz, 2H), 3.13 (dt, *J* = 7.6, 2.4 Hz, 2H), 2.51-2.54 (m, 2H), 1.54-1.61 (m, 2H), 1.33-1.40 (m, 2H), 1.23-1.32 (m, 6H), 0.86-0.90 (m, 3H) ppm. **^13^C NMR** (101 MHz, CDCl_3_): δ = 209.61 (s, 1C), 88.19 (d, 1C), 75.98 (t, 1C), 31.88 (t, 1C), 31.04 (t, 1C), 30.79 (t, 1C), 29.40 (t, 1C), 29.04 (t, 2C), 22.75 (t, 1C), 14.22 (q, 1C) ppm. **IR** (Reflexion)**:** ṽ = 2955, 2923, 2854, 1950, 1682, 1557, 1465, 1378, 1224, 1057, 845, 724 cm^-1^. **HR-MS** (EI(+)): *m/z* = 185.1376, calcd. for [M+H]^+^ = [C_11_H_21_S]^+^: 185.1358.

**2-(4-Fluorophenyl)-2-(phenylthio)pent-4-enenitrile (3aa)**

The product was prepared according to **GP4**, using 49.5 mg of **1a** (0.20 mmol, 1.00 eq.) and 301 mg of **2a** (2.00 mmol, 10.0 eq.) in 4 mL of DCM. Purification by flash column chromatography gave the product as a yellow oil (39.8 mg, 70%).

**R_f_** (SiO_2_, PE/Et_2_O, 15:1): 0.40; **^1^H NMR** (300 MHz, CDCl_3_): δ = 7.27-7.38 (m, 5H), 7.17-7.22 (m, 2H), 6.90-6.97 (m, 2H), 5.54-5.68 (m, 1H), 5.09-5.15 (m, 2H), 2.84-2.96 (m, 2H) ppm. **^13^C NMR** (75 MHz, CDCl_3_): δ = 162.53 (s, d: *J*_C-F_ = 249.0 Hz, 1C), 136.78 (d, 2C), 131.91 (s, d: *J*_C-F_ = 3.2 Hz, 1C), 130.53 (d, 1C), 130.43 (d, 1C), 129.41 (s, 1C), 129.11 (d, 2C), 128.89 (d, d: J_C-F_ = 8.3 Hz, 2C), 121.15 (t, 1C), 119.43 (s, 1C), 115.65 (d, d: *J*_C-F_ = 21.9 Hz, 2C), 53.50 (s, 1C), 44.46 (t, 1C) ppm. **^19^F NMR** (283 MHz, CDCl_3_): δ **=** -112.79 (s, 1F) ppm. **IR** (Reflexion)**:** ṽ = 3078, 2983, 2923, 2232, 1641, 1600, 1508, 1475, 1439, 1412, 1306, 1236, 1164, 1105, 1068, 1014, 993, 927, 816, 790, 749, 690 cm^-1^. **HR-MS** (EI(+)): *m/z* = 283.0820, calcd. for [M]^+^ = [C_17_H_14_FNS]^+^: 283.0826.

**2-Phenyl-2-(phenylthio)pent-4-enenitrile (3ba)**

The product was prepared according to **GP4**, using 45.8 mg of **1b** (0.20 mmol, 1.00 eq.) and 301 mg of **2a** (2.00 mmol, 10.0 eq.) in 4 mL of DCM. Purification by flash column chromatography gave the product as a yellow oil (33.9 mg, 64%).

**R_f_** (SiO_2_, PE/Et_2_O, 15:1): 0.39; **^1^H NMR** (500 MHz, CDCl_3_): δ = 7.42-7.44 (m, 2H), 7.34-7.37 (m, 3H), 7.27-7.33 (m, 3H), 7.23-7.26 (m, 2H), 5.62- 5.71 (m, 1H), 5.15-5.21 (m, 2H), 2.92-3.04 (m, 2H) ppm. **^13^C NMR** (126 MHz, CDCl_3_): δ = 136.87 (d, 2C), 135.98 (s, 1C), 130.82 (d, 1C), 130.35 (d, 1C), 129.65 (s, 1C), 129.07 (d, 2C), 128.74 (d, 2C), 128.69 (d, 1C), 127.03 (d, 2C), 121.00 (t, 1C), 119.58 (s, 1C), 54.14 (s, 1C), 44.45 (t, 1C) ppm. **IR** (Reflexion)**:** ṽ = 3078, 3061, 2982, 2921, 2232, 1956, 1885, 1640, 1598, 1583, 1490, 1475, 1439, 1416, 1333, 1306, 1289, 1262, 1179, 1159, 1133, 1085, 1068, 1024, 993, 927, 863, 748, 691, 653 cm^-1^. **HR-MS** (EI(+)): *m/z* = 265.0925, calcd. for [M]^+^ = [C_17_H_15_NS]^+^: 265.0920.

**2-(4-Chlorophenyl)-2-(phenylthio)pent-4-enenitrile (3ca)**

The product was prepared according to **GP4**, using 52.6 mg of **1c** (0.20 mmol, 1.00 eq.) and 301 mg of **2a** (2.00 mmol, 10.0 eq.) in 4 mL of DCM. Purification by flash column chromatography gave the product as a yellow oil (34.8 mg, 58%).

**R_f_** (SiO_2_, PE/Et_2_O, 15:1): 0.39; **^1^H NMR** (600 MHz, CDCl_3_): δ = 7.33-7.37 (m, 5H), 7.24-7.28 (m, 4H), 5.60-5.67 (m, 1H), 5.15-5.19 (m, 2H), 2.91-2.97 (m, 2H) ppm. **^13^C NMR** (151 MHz, CDCl_3_): δ = 136.84 (d, 2C), 134.75 (s 1C), 134.64 (s, 1C), 130.56 (d, 1C), 130.44 (d, 1C), 129.29 (s, 1C), 129.22 (d, 2C), 128.92 (d, 2C), 128.45 (d, 2C), 121.36 (t, 1C), 119.27 (s, 1C), 53.68 (s, 1C), 44.40 (t, 1C) ppm. **IR** (Reflexion)**:** ṽ = 3079, 3062, 2982, 2919, 2849, 2233, 1641, 1593, 1574, 1492, 1475, 1439, 1402, 1306, 1262, 1178, 1096, 1068, 1014, 994, 929, 834, 793, 750, 719, 692 cm^-1^. **HR-MS** (EI(+)): *m/z* = 299.0505, calcd. for [M]^+^ = [C_17_H_14_ClNS]^+^: 299.0530.

**2-(4-Bromophenyl)-2-(phenylthio)pent-4-enenitrile (3da)**

The product was prepared according to **GP4**, using 61.4 mg of **1d** (0.20 mmol, 1.00 eq.) and 301 mg of **2a** (2.00 mmol, 10.0 eq.) in 4 mL of DCM. Purification by flash column chromatography gave the product as a yellow oil (38.5 mg, 56%).

**R_f_** (SiO_2_, PE/Et_2_O, 15:1): 0.38; **^1^H NMR** (500 MHz, CDCl_3_): δ = 7.43-7.46 (m, 2H), 7.36-7.39 (m, 3H), 7.26-7.30 (m, 4H), 5.61-5.69 (m, 1H), 5.16-5.20 (m, 2H), 2.91-2.98 (m, 2H) ppm. **^13^C NMR** (126 MHz, CDCl_3_): δ =136.84 (d, 2C), 135.31 (s 1C), 131.89 (d, 2C), 130.58 (d, 1C), 130.42 (d, 1C), 129.27 (s, 1C), 129.24 (d, 2C), 128.74 (d, 2C), 122.82 (s, 1C), 121.39 (t, 1C), 119.21 (s, 1C), 53.76 (s, 1C), 44.37 (t, 1C) ppm. **IR** (Reflexion)**:** ṽ = 3079, 3062, 3012, 2981, 2919, 2232, 1900, 1640, 1587, 1487, 1439, 1397, 1306, 1118, 1076, 1024, 1010, 928, 830, 789, 749, 691, 668 cm^-1^. **HR-MS** (EI(+)): *m/z* = 343.0022, calcd. for [M]^+^ = [C_17_H_14_BrNS]^+^: 343.0025.

**2-(Phenylthio)-2-(p-tolyl)pent-4-enenitrile (3fa)**

The product was prepared according to **GP4**, using 48.6 mg of **1f** (0.20 mmol, 1.00 eq.) and 301 mg of **2a** (2.00 mmol, 10.0 eq.) in 4 mL of DCM. Purification by flash column chromatography gave the product as a yellow oil (42.9 mg, 77%).

**R_f_** (SiO_2_, PE/Et_2_O, 15:1): 0.45; **^1^H NMR** (500 MHz, CDCl_3_): δ = 7.39 (d, *J* = 7.8 Hz, 2H), 7.35 (d, *J* = 7.4 Hz, 1H), 7.32 (d, *J* = 7.9 Hz, 2H), 7.26 (t, *J* = 7.6 Hz, 2H), 7.12 (d, *J* = 7.9 Hz, 2H), 5.61-5.69 (m, 1H), 5.13-5.18 (m, 2H), 2.99 (dd, *J* = 14.1, 6.7 Hz, 1H), 2.90 (dd, *J* = 14.1, 7.6 Hz, 1H), 2.33 (s, 3H) ppm. **^13^C NMR** (126 MHz, CDCl_3_): δ = 138.64 (s, 1C), 136.88 (d, 2C), 132.89 (s, 1C), 130.96 (d, 1C), 130.30 (d, 1C), 129.85 (s, 1C), 129.44 (d, 2C), 129.06 (d, 2C), 126.92 (d, 2C), 120.84 (t, 1C), 119.73 (s, 1C), 53.88 (s, 1C), 44.49 (t, 1C), 21.20 (q, 1C) ppm. **IR** (Reflexion)**:** ṽ = 3078, 3059, 3028, 2981, 2921, 2232, 1641, 1573, 1511, 1475, 1439, 1306, 1263, 1191, 1124, 1068, 1023, 993, 926, 800, 778, 749, 717, 691 cm^-1^. **HR-MS** (EI(+)): *m/z* = 279.1067, calcd. for [M]^+^ = [C_18_H_17_NS]^+^: 279.1076.

**2-(4-(Tert-butyl)phenyl)-2-(phenylthio)pent-4-enenitrile (3ga)**

The product was prepared according to **GP4**, using 57.0 mg of **1g** (0.20 mmol, 1.00 eq.) and 301 mg of **2a** (2.00 mmol, 10.0 eq.) in 4 mL of DCM. Purification by flash column chromatography gave the product as a yellow oil (48.7 mg, 76%).

**R_f_** (SiO_2_, PE/Et_2_O, 15:1): 0.45; **^1^H NMR** (400 MHz, CDCl_3_): δ = 7.31-7.39 (m, 7H), 7.22-7.27 (m, 2H), 5.64-5.74 (m, 1H), 5.14-5.22 (m, 2H), 3.03 (dd, *J* = 14.1, 6.6 Hz, 1H), 2.91 (dd, *J* = 14.1, 7.7 Hz, 1H), 1.31 (s, 9H) ppm. **^13^C NMR** (101 MHz, CDCl_3_): δ = 151.92 (s, 1C), 136.96 (d, 2C), 132.78 (s, 1C), 131.05 (d, 1C), 130.26 (d, 1C), 129.95 (s, 1C), 128.98 (d, 2C), 126.72 (d, 2C), 125.64 (d, 2C), 120.77 (t, 1C), 119.74 (s, 1C), 53.78 (s, 1C), 44.25 (t, 1C), 34.70 (s, 1C), 31.34 (q, 3C) ppm. **IR** (Reflexion)**:** ṽ = 3079, 3060, 2963, 2905, 2869, 2232, 1641, 1609, 1574, 1507, 1475, 1462, 1439, 1414, 1364, 1269, 1203, 1125, 1108, 1068, 1023, 993, 926, 835, 798, 750, 726, 705, 691 cm^-1^. **HR-MS** (EI(+)): *m/z* = 321.1556, calcd. for [M]^+^ = [C_21_H_23_NS]^+^: 321.1546.

**2-(2-Fluorophenyl)-2-(phenylthio)pent-4-enenitrile (3ha)**

The product was prepared according to **GP4**, using 49.4 mg of **1h** (0.20 mmol, 1.00 eq.) and 301 mg of **2a** (2.00 mmol, 10.0 eq.) in 4 mL of DCM. Purification by flash column chromatography gave the product as a yellow oil (36.9 mg, 65%).

**R_f_** (SiO_2_, PE/Et_2_O, 15:1): 0.41; **^1^H NMR** (400 MHz, CDCl_3_): δ = 7.33-7.37 (m, 3H), 7.28-7.32 (m, 1H), 7.22-7.27 (m, 2H), 7.08-7.18 (m, 2H), 6.93-6.97 (m, 1H), 5.67-5.78 (m, 1H), 5.25 (dd, *J* = 17.1, 1.5 Hz, 1H), 5.16 (dd, *J* = 10.3, 1.5 Hz, 1H), 3.30-3.36 (m, 1H), 3.01-3.07 (m, 1H) ppm. **^13^C NMR** (101 MHz, CDCl_3_): δ = 160.03 (s, d: *J*_C-F_ = 251.8 Hz, 1C), 137.10 (d, 2C), 130.99 (d, 1C), 130.90 (d, 1C), 130.51 (d, 1C), 130.18 (d, d: *J*_C-F_ = 3.0 Hz, 1C), 129.47 (s, 1C), 129.04 (d, 2C), 124.03 (d, d: *J*_C-F_ = 3.6 Hz, 1C), 122.89 (s, d: *J*_C-F_ = 8.9 Hz, 1C), 120.92 (t, 1C), 119.17 (s, 1C), 117.03 (d, d: *J*_C-F_ = 22.2 Hz, 1C), 52.83 (s, d: *J*_C-F_ = 4.5 Hz, 1C), 41.96 (t, d: *J*_C-F_ = 6.4 Hz, 1C) ppm. **^19^F NMR** (283 MHz, CDCl_3_): δ **=** -108.52 (s, 1F) ppm. **IR** (Reflexion)**:** ṽ = 3079, 3064, 3011, 2983, 2925, 2854, 2233, 1957, 1803, 1641, 1612, 1582, 1490, 1475, 1440, 1417, 1280, 1226, 1186, 1159, 1141, 1099, 1068, 1024, 993, 928, 862, 816, 750, 705, 691, 629 cm^-1^. **HR-MS** (EI(+)): *m/z* = 283.0831, calcd. for [M]^+^ = [C_17_H_14_FNS]^+^: 283.0826.

**2-(Phenylthio)-2-(o-tolyl)pent-4-enenitrile (3ia)**

The product was prepared according to **GP4**, using 48.6 mg of **1i** (0.20 mmol, 1.00 eq.) and 301 mg of **2a** (2.00 mmol, 10.0 eq.) in 4 mL of DCM. Purification by flash column chromatography gave the product as a yellow oil (43.3 mg, 78%).

**R_f_** (SiO_2_, PE/Et_2_O, 15:1): 0.44; **^1^H NMR** (300 MHz, CDCl_3_): δ = 7.31-7.37 (m, 3H), 7.20-7.29 (m, 3H), 7.16-7.19 (m, 2H), 7.00-7.07 (m, 1H), 5.59-5.73 (m, 1H), 5.10-5.23 (m, 2H), 3.24 (dd, *J* = 14.6, 6.6 Hz, 1H), 2.91 (dd, *J* = 14.6, 7.4 Hz, 1H), 2.73 (s, 3H) ppm. **^13^C NMR** (75 MHz, CDCl_3_): δ = 136.97 (d, 2C), 136.69 (s, 1C), 133.35 (d, 1C), 132.15 (s, 1C), 131.18 (d, 1C), 130.36 (d, 1C), 129.75 (s, 1C), 129.13 (d, 1C), 129.02 (d, 2C), 128.93 (d, 1C), 126.14 (d, 1C), 120.49 (s, 1C), 120.23 (t, 1C), 54.42 (s, 1C), 42.39 (t, 1C), 22.00 (q, 1C) ppm. **IR** (Reflexion)**:** ṽ = 3070, 2978, 2230, 1453, 1439, 1135, 1068, 985, 931, 754, 705, 691 cm^-1^. **HR-MS** (EI(+)): *m/z* = 279.1090, calcd. for [M]^+^ = [C_18_H_17_NS]^+^: 279.1076.

**2-(3-Fluorophenyl)-2-(phenylthio)pent-4-enenitrile (3ja)**

The product was prepared according to **GP4**, using 49.4 mg of **1j** (0.20 mmol, 1.00 eq.) and 301 mg of **2a** (2.00 mmol, 10.0 eq.) in 4 mL of DCM. Purification by flash column chromatography gave the product as a yellow oil (18.8 mg, 33%).

**R_f_** (SiO_2_, PE/Et_2_O, 15:1): 0.40; **^1^H NMR** (500 MHz, CDCl_3_): δ = 7.35-7.38 (m, 3H), 7.25-7.29 (m, 3H), 7.15-7.21 (m, 2H), 6.97-7.01 (m, 1H), 5.62-5.70 (m, 1H), 5.17-5.22 (m, 2H), 2.91-2.99 (m, 2H) ppm. **^13^C NMR** (126 MHz, CDCl_3_): δ = 162.81 (s, d: *J*_C-F_ = 247.3 Hz, 1C), 138.75 (s, d: *J*_C-F_ = 7.1 Hz, 1C), 136.82 (d, 2C), 130.58 (d, 1C), 130.40 (d, 1C), 130.30 (d, d: *J*_C-F_ = 8.1 Hz, 1C), 129.25 (s, 1C), 129.20 (d, 2C), 122.86 (d, d: *J*_C-F_ = 3.2 Hz, 1C), 121.37 (t, 1C), 119.21 (s, 1C), 115.78 (d, d: *J*_C-F_ = 21.3 Hz, 1C), 114.28 (d, d: *J*_C-F_ = 23.7 Hz, 1C), 53.80 (s, d: *J*_C-F_ = 2.3 Hz, 1C), 44.43 (t, 1C) ppm. **^19^F NMR** (283 MHz, CDCl_3_): δ **=** -111.56 (s, 1F) ppm. **IR** (Reflexion)**:** ṽ = 3079, 3065, 2983, 2923, 2234, 1641, 1612, 1591, 1489, 1441, 1270, 1233, 1160, 1068, 1024, 988, 929, 897, 877, 832, 775, 749, 706, 690 cm^-1^. **HR-MS** (EI(+)): *m/z* = 283.0839, calcd. for [M]^+^ = [C_17_H_14_FNS]^+^: 283.0826.

**2-(Phenylthio)-2-(m-tolyl)pent-4-enenitrile (3ka)**

The product was prepared according to **GP4**, using 48.2 mg of **1k** (0.20 mmol, 1.00 eq.) and 301 mg of **2a** (2.00 mmol, 10.0 eq.) in 4 mL of DCM. Purification by flash column chromatography gave the product as a yellow oil (36.2 mg, 65%).

**R_f_** (SiO_2_, PE/Et_2_O, 15:1): 0.45; **^1^H NMR** (500 MHz, CDCl_3_): δ = 7.34-7.38 (m, 3H), 7.24-7.27 (m, 3H), 7.19-7.22 (m, 2H), 7.09 (d, *J* = 7.3 Hz, 1H), 5.61-5.69 (m, 1H), 5.14-5.19 (m, 2H), 3.01 (dd, *J* = 14.1, 6.5 Hz, 1H), 2.90 (dd, *J* = 14.1, 7.7 Hz, 1H), 2.31 (s, 3H) ppm. **^13^C NMR** (126 MHz, CDCl_3_): δ = 138.54 (s, 1C), 136.91 (d, 2C), 135.77 (s, 1C), 130.94 (d, 1C), 130.34 (d, 1C), 129.79 (s, 1C), 129.45 (d, 1C), 129.04 (d, 2C), 128.65 (d, 1C), 127.73 (d, 1C), 123.99 (d, 1C), 120.88 (t, 1C), 119.69 (s, 1C), 54.03 (s, 1C), 44.37 (t, 1C), 21.56 (q, 1C) ppm. **IR** (Reflexion)**:** ṽ = 3078, 3060, 3023, 2981, 2954, 2921, 2861, 2232, 1954, 1884, 1641, 1606, 1586, 1511, 1475, 1439, 1306, 1265, 1177, 1095, 1068, 1024, 991, 927, 882, 750, 692 cm^-1^. **HR-MS** (EI(+)): *m/z* = 279.1070, calcd. for [M]^+^ = [C_18_H_17_NS]^+^: 279.1076.

**2-(2,6-Dimethylphenyl)-2-(phenylthio)pent-4-enenitrile (3la)**

The product was prepared according to **GP4**, using 51.4 mg of **1l** (0.20 mmol, 1.00 eq.) and 301 mg of **2a** (2.00 mmol, 10.0 eq.) in 4 mL of DCM. Purification by flash column chromatography gave the product as a yellow oil (37.5 mg, 64%).

**R_f_** (SiO_2_, PE/Et_2_O, 15:1): 0.46; **^1^H NMR** (400 MHz, CDCl_3_): δ = 7.49-7.52 (m, 2H), 7.40-7.45 (m, 1H), 7.29-7.34 (m, 2H), 7.07-7.10 (m, 1H), 6.98 (d, *J* = 7.5 Hz, 2H), 5.59-5.69 (m, 1H), 5.22 (dq, *J* = 17.0, 1.5 Hz, 1H), 5.12 (dq, *J* = 10.1, 1.2 Hz, 1H), 3.61 (ddt, *J* = 14.8, 6.2, 1.4 Hz, 1H), 2.81 (ddt, *J* = 14.9, 7.8, 1.2 Hz, 1H), 2.59 (s, 6H) ppm. **^13^C NMR** (101 MHz, CDCl_3_): δ = 138.05 (s, 2C), 137.34 (d, 2C), 131.67 (d, 2C), 131.32 (s, 1C), 131.12 (d, 1C), 130.56 (d, 1C), 130.03 (s, 1C), 129.14 (d, 2C), 128.26 (d, 1C), 122.49 (s, 1C), 120.36 (t, 1C), 53.24 (s, 1C), 44.40 (t, 1C), 24.31 (q, 2C) ppm. **IR** (Reflexion)**:** ṽ = 3062, 3014, 2979, 2940, 2889, 2746, 2224, 1952, 1861, 1640, 1575, 1459, 1439, 1417, 1378, 1305, 1274, 1169, 1137, 1100, 1068, 1025, 991, 926, 772, 750, 717, 705, 691cm^-1^. **HR-MS** (EI(+)): *m/z* = 293.1227, calcd. for [M]^+^ = [C_19_H_19_NS]^+^: 293.1233.

**2-(Naphthalen-2-yl)-2-(phenylthio)pent-4-enenitrile (3ma)**

The product was prepared according to **GP4**, using 55.8 mg of **1m** (0.20 mmol, 1.00 eq.) and 301 mg of **2a** (2.00 mmol, 10.0 eq.) in 4 mL of DCM. Purification by flash column chromatography gave the product as a yellow oil (41.8 mg, 66%).

**R_f_** (SiO_2_, PE/Et_2_O, 15:1): 0.42; **^1^H NMR** (500 MHz, CDCl_3_): δ = 7.88 (d, *J* = 8.7 Hz, 1H), 7.83 (d, *J* = 7.1 Hz, 1H), 7.73-7.75 (m, 2H), 7.67 (dd, *J* = 8.7, 2.0 Hz, 1H), 7.47-7.53 (m, 2H), 7.33-7.35 (m, 2H), 7.28-7.32 (m, 1H), 7.16-7.19 (m, 2H), 5.63-5.71 (m, 1H), 5.21 (dd, *J* = 17.0, 1.5 Hz, 1H), 5.14 (dd, *J* = 10.1, 1.4 Hz, 1H), 3.11 (dd, *J* = 14.1, 6.7 Hz, 1H), 3.03 (dd, *J* = 14.1, 7.6 Hz, 1H) ppm. **^13^C NMR** (126 MHz, CDCl_3_): δ = 136.82 (d, 2C), 133.04 (s, 1C), 133.02 (s, 1C), 132.72 (s, 1C), 130.78 (d, 1C), 130.38 (d, 1C), 129.56 (s, 1C), 129.07 (d, 2C), 129.01 (d, 1C), 128.44 (d, 1C), 127.66 (d, 1C), 127.17 (d, 1C), 127.03 (d, 1C), 126.85 (d, 1C), 123.48 (d, 1C), 121.06 (t, 1C), 119.61 (s, 1C), 54.55 (s, 1C), 44.26 (t, 1C) ppm. **IR** (Reflexion)**:** ṽ = 3059, 3020, 2981, 2920, 2232, 1641, 1599, 1507, 1475, 1439, 1416, 1359, 1306, 1274, 1244, 1204, 1177, 1133, 1068, 1024, 988, 929, 898, 863, 816, 789, 747, 706, 691, 628 cm^-1^. **HR-MS** (EI(+)): *m/z* = 315.1076, calcd. for [M]^+^ = [C_21_H_17_NS]^+^: 315.1076.

**2-(Phenylthio)-2-(thiophen-2-yl)pent-4-enenitrile (3na)**

The product was prepared according to **GP4**, using 47.0 mg of **1n** (0.20 mmol, 1.00 eq.) and 301 mg of **2a** (2.00 mmol, 10.0 eq.) in 4 mL of DCM. Purification by flash column chromatography gave the product as a yellow oil (40.7 mg, 75%).

**R_f_** (SiO_2_, PE/Et_2_O, 15:1): 0.40; **^1^H NMR** (300 MHz, CDCl_3_): δ = 7.35-7.43 (m, 3H), 7.25-7.30 (m, 3H), 6.76-6.81 (m, 2H), 5.73-5.87 (m, 1H), 5.21-5.29 (m, 2H), 3.00 (d, *J* = 7.1 Hz, 2H) ppm. **^13^C NMR** (75 MHz, CDCl_3_): δ = 140.93 (s, 1C), 136.83 (d, 2C), 130.56 (d, 1C), 130.55 (d, 1C), 129.65 (s, 1C), 129.13 (d, 2C), 128.20 (d, 1C), 126.91 (d, 1C), 126.43 (d, 1C), 121.37 (t, 1C), 119.05 (s, 1C), 51.09 (s, 1C), 45.89 (t, 1C) ppm. **IR** (Reflexion)**:** ṽ = 3078, 2982, 2916, 2849, 2234, 1806, 1732, 1641, 1574, 1475, 1438, 1353, 1306, 1239, 1177, 1068, 1024, 991, 928, 831, 782, 750, 705, 691 cm^-1^. **HR-MS** (EI(+)): *m/z* = 271.0473, calcd. for [M]^+^ = [C_15_H_13_NS_2_]^+^: 271.0484.

**2-(4-Fluorophenyl)-2-((4-fluorophenyl)thio)pent-4-enenitrile (3ab)**

The product was prepared according to **GP4**, using 74.2 mg of **1a** (0.30 mmol, 1.00 eq.) and 505mg of **2b** (3.00 mmol, 10.0 eq.) in 6 mL of DCM. Purification by flash column chromatography gave the product as a yellow oil (62.5 mg, 69%).

**R_f_** (SiO_2_, PE/Et_2_O, 15:1): 0.44; **^1^H NMR** (500 MHz, CDCl_3_): δ = 7.36-7.39 (m, 2H), 7.30-7.34 (m, 2H), 6.99-7.04 (m, 2H), 6.93-6.98 (m, 2H), 5.62-5.70 (m, 1H), 5.17-5.22 (m, 2H), 2.92-3.00 (m, 2H) ppm. **^13^C NMR** (126 MHz, CDCl_3_): δ = 164.51 (s, d: *J*_C-F_ = 225.0 Hz, 1C), 162.52 (s, d: *J*_C-F_ = 222.5 Hz, 1C), 139.05 (d, d: *J*_C-F_ = 9.2 Hz, 2C), 131.81 (s, d: *J*_C-F_ = 3.1 Hz, 1C), 130.43 (d, 1C), 128.91 (d, d: *J*_C-F_ = 8.2 Hz, 2C), 124.82 (s, d: *J*_C-F_ = 3.4 Hz, 1C), 121.32 (t, 1C), 119.30 (s, 1C), 116.43 (d, d: *J*_C-F_ = 21.9 Hz, 2C), 115.80 (d, d: *J*_C-F_ = 21.9 Hz, 2C), 53.88 (s, 1C), 44.25 (t, 1C) ppm. **^19^F NMR** (283 MHz, CDCl_3_): δ **=** -109.25 (s, 1F), -112.47 (s, 1F) ppm. **IR** (Reflexion)**:** ṽ = 3080, 3013, 2983, 2922, 2233, 1895, 1642, 1589, 1509, 1489, 1440, 1412, 1398, 1306, 1291, 1235, 1158, 1106, 1091, 1014, 994, 929, 834, 817, 720, 695, 637 cm^-1^. **HR-MS** (EI(+)): *m/z* = 301.0742, calcd. for [M]^+^ = [C_17_H_13_F_2_NS]^+^: 301.0731.

**2-((4-Chlorophenyl)thio)-2-(4-fluorophenyl)pent-4-enenitrile (3ac)**

The product was prepared according to **GP4**, using 74.2 mg of **1a** (0.30 mmol, 1.00 eq.) and 554 mg of **2c** (3.00 mmol, 10.0 eq.) in 6 mL of DCM. Purification by flash column chromatography gave the product as a yellow oil (65.6 mg, 69%).

**R_f_** (SiO_2_, PE/Et_2_O, 15:1): 0.38; **^1^H NMR** (400 MHz, CDCl_3_): δ = 7.39-7.44 (m, 2H), 7.23-7.30 (m, 4H), 7.01-7.07 (m, 2H), 5.62-5.73 (m, 1H), 5.19-5.24 (m, 2H), 2.94-3.00 (m, 2H) ppm. **^13^C NMR** (101 MHz, CDCl_3_): δ = 162.69 (s, d: *J*_C-F_ = 249.5 Hz, 1C), 138.02 (d, 2C), 137.27 (s, 1C), 131.79 (s, d: *J*_C-F_ = 3.3 Hz, 1C), 130.37 (d, 1C), 129.48 (d, 2C), 128.95 (d, d: *J*_C-F_ = 8.4 Hz, 2C), 127.92 (s, 1C), 121.42 (t, 1C), 119.25 (s, 1C), 115.87 (d, d: *J*_C-F_ = 22.0 Hz, 2C), 53.83 (s, 1C), 44.51 (t, 1C) ppm. **^19^F NMR** (283 MHz, CDCl_3_): δ **=** -112.32 (s, 1F) ppm. **IR** (Reflexion)**:** ṽ = 3082, 2983, 2233, 1642, 1601, 1573, 1509, 1476, 1413, 1390, 1236, 1164, 1094, 1014, 929, 823, 748 cm^-1^. **HR-MS** (EI(+)): *m/z* = 317.0463, calcd. for [M]^+^ = [C_17_H_13_ClFNS]^+^: 317.0436.

**2-((4-Bromophenyl)thio)-2-(4-fluorophenyl)pent-4-enenitrile (3ad)**

The product was prepared according to **GP4**, using 74.2 mg of **1a** (0.30 mmol, 1.00 eq.) and 687 mg of **2d** (3.00 mmol, 10.0 eq.) in 6 mL of DCM. Purification by flash column chromatography gave the product as a yellow oil (78.1 mg, 72%).

**R_f_** (SiO_2_, PE/Et_2_O, 15:1): 0.39; **^1^H NMR** (600 MHz, CDCl_3_): δ = 7.38-7.41 (m, 4H), 7.18-7.20 (m, 2H), 7.00-7.04 (m, 2H), 5.62-5.68 (m, 1H), 5.18-5.21 (m, 2H), 2.92-2.98 (m, 2H) ppm. **^13^C NMR** (151 MHz, CDCl_3_): δ = 162.65 (s, d: *J*_C-F_ = 249.6 Hz, 1C), 138.20 (d, 2C), 132.44 (d, 2C), 131.71 (s, d: *J*_C-F_ = 3.3 Hz, 1C), 130.32 (d, 1C), 128.92 (d, d: *J*_C-F_ = 8.3 Hz, 2C), 128.46 (s, 1C), 125.65 (s, 1C), 121.46 (t, 1C), 119.22 (s, 1C), 115.88 (d, d: *J*_C-F_ = 21.7 Hz, 2C), 53.72 (s, 1C), 44.49 (t, 1C) ppm. **^19^F NMR** (283 MHz, CDCl_3_): δ **=** -112.31 (s, 1F) ppm. **IR** (Reflexion)**:** ṽ = 3081, 2926, 2854, 2233, 1646, 1601, 1566, 1508, 1472, 1412, 1386, 1306, 1236, 1164, 1091, 1069, 1010, 928, 817, 731 cm^-1^. **HR-MS** (EI(+)): *m/z* = 360.9921, calcd. for [M]^+^ = [C_17_H_13_BrFNS]^+^: 360.9931.

**2-(4-Fluorophenyl)-2-((4-(trifluoromethyl)phenyl)thio)pent-4-enenitrile (3ae)**

The product was prepared according to **GP4**, using 74.2 mg of **1a** (0.30 mmol, 1.00 eq.) and 655 mg of **2e** (3.00 mmol, 10.0 eq.) in 6 mL of DCM. Purification by flash column chromatography gave the product as a yellow solid (72.1 mg, 68%).

**R_f_** (SiO_2_, PE/Et_2_O, 15:1): 0.40; **^1^H NMR** (500 MHz, CDCl_3_): δ = 7.51 (d, *J* = 8.1 Hz, 2H), 7.41-7.46 (m, 4H), 7.03 (t, *J* = 8.4 Hz, 2H), 5.62-5.71 (m, 1H), 5.20-5.23 (m, 2H), 2.94- 3.01 (m, 2H) ppm. **^13^C NMR** (126 MHz, CDCl_3_): δ = 162.72 (s, d: *J*_C-F_ = 249.7 Hz, 1C), 136.56 (d, 2C), 134.12 (s, q: *J*_C-F_ = 1.3Hz, 1C), 132.23 (s, q, *J*_C-F_ = 32.9 Hz, 1C), 131.58 (s, d: *J*_C-F_ = 3.0 Hz, 1C), 130.13 (d, 1C), 128.89 (d, d: *J*_C-F_ = 8.2 Hz, 2C), 125.97 (d, q: *J*_C-F_ = 3.8 Hz, 2C), 123.77 (q, q: *J*_C-F_ = 273.4 Hz, 1C), 121.67 (t, 1C) 119.12 (s, 1C), 115.96 (d, d: *J*_C-F_ = 21.9 Hz, 2C), 53.71 (s, 1C), 44.80 (t, 1C) ppm. **^19^F NMR** (283 MHz, CDCl_3_): δ **=** -62.97 (s, 3F), -112.06 (s, 1F) ppm. **IR** (Reflexion)**:** ṽ = 3083, 2985, 2924, 2234, 1643, 1605, 1509, 1440, 1400, 1327, 1239, 1167, 1133, 1104, 1063, 1016, 994, 931, 839, 792, 704 cm^-1^. **HR-MS** (EI(+)): *m/z* = 351.0698, calcd. for [M]^+^ = [C_18_H_13_F_4_NS]^+^: 351.0699; **m.p.** = 59.1-60.2 °C.

**2-(4-Fluorophenyl)-2-(p-tolylthio)pent-4-enenitrile (3af)**

The product was prepared according to **GP4**, using 74.2 mg of **1a** (0.30 mmol, 1.00 eq.) and 493 mg of **2f** (3.00 mmol, 10.0 eq.) in 6 mL of DCM. Purification by flash column chromatography gave the product as a yellow oil (60.8 mg, 68%).

**R_f_** (SiO_2_, PE/Et_2_O, 15:1): 0.40; **^1^H NMR** (500 MHz, CDCl_3_): δ = 7.37-7.41 (m, 2H), 7.22-7.25 (m, 2H), 7.05-7.07 (m, 2H), 6.97-7.02 (m, 2H), 5.59-5.68 (m, 1H), 5.13-5.17 (m, 2H), 2.88-2.96 (m, 2H), 2.30 (s, 3H) ppm. **^13^C NMR** (126 MHz, CDCl_3_): δ = 162.57 (s, d: *J*_C-F_ = 248.8 Hz, 1C), 140.95 (s, 1C), 136.85 (d, 2C), 132.07 (s, d: *J*_C-F_ = 3.0 Hz, 1C), 130.67 (d, 1C), 129.98 (d, 2C), 128.96 (d, d: *J*_C-F_ = 8.6 Hz, 2C), 125.97 (s, 1C), 121.08 (t, 1C), 119.53 (s, 1C), 115.67 (d, d: *J*_C-F_ = 22.0 Hz, 2C), 53.59 (s, 1C), 44.46 (t, 1C), 21.44 (q, 1C) ppm. **^19^F NMR** (283 MHz, CDCl_3_): δ **=** -112.90 (s, 1F) ppm. **IR** (Reflexion)**:** ṽ = 3078, 3023, 2982, 2922, 2865, 2232, 1642, 1600, 1509, 1441, 1412, 1305, 1237, 1182, 1164, 1106, 1016, 994, 928, 841, 811, 721 cm^-1^. **HR-MS** (EI(+)): *m/z* = 297.0980, calcd. for [M]^+^ = [C_18_H_16_FNS]^+^: 297.0982.

**2-((4-(Tert-butyl)phenyl)thio)-2-(4-fluorophenyl)pent-4-enenitrile (3ag)**

The product was prepared according to **GP4**, using 74.2 mg of **1a** (0.30 mmol, 1.00 eq.) and 619 mg of **2g** (3.00 mmol, 10.0 eq.) in 6 mL of DCM. Purification by flash column chromatography gave the product as a yellow oil (69.8 mg, 69%).

**R_f_** (SiO_2_, PE/Et_2_O, 15:1): 0.44; **^1^H NMR** (400 MHz, CDCl_3_): δ = 7.39-7.44 (m, 2H), 7.27-7.32 (m, 4H), 6.97-7.03 (m, 2H), 5.61-5.71 (m, 1H), 5.14-5.19 (m, 2H), 2.90-3.00 (m, 2H), 1.29 (s, 9H) ppm. **^13^C NMR** (101 MHz, CDCl_3_): δ = 162.62 (s, d: *J*_C-F_ = 248.8 Hz, 1C), 154.01 (s, 1C), 136.63 (d, 2C), 132.04 (s, d: *J*_C-F_ = 3.3 Hz, 1C), 130.74 (d, 1C), 128.99 (d, d: *J*_C-F_ = 8.4 Hz, 2C), 126.27 (d, 2C), 126.06 (s, 1C), 121.07 (t, 1C), 119.65 (s, 1C), 115.65 (d, d: *J*_C-F_ = 21.8 Hz, 2C), 53.44 (s, 1C), 44.47 (t, 1C), 34.91 (s, 1C), 31.27 (q, 3C) ppm. **^19^F NMR** (283 MHz, CDCl_3_): δ **=** -112.94 (s, 1F) ppm. **IR** (Reflexion)**:** ṽ = 3081, 2964, 2906, 2870, 2232, 1642, 1601, 1509, 1489, 1462, 1396, 1364, 1306, 1268, 1237, 1164, 1117, 1087, 1014, 993, 927, 832, 791 cm^-1^. **HR-MS** (EI(+)): *m/z* = 339.1430, calcd. for [M]^+^ = [C_21_H_22_FNS]^+^: 339.1452.

**2-(4-Fluorophenyl)-2-((2-fluorophenyl)thio)pent-4-enenitrile (3ah)**

The product was prepared according to **GP4**, using 24.7 mg of **1a** (0.10 mmol, 1.00 eq.) and 168 mg of **2h** (1.00 mmol, 10.0 eq.) in 2 mL of DCM. Purification by flash column chromatography gave the product as a yellow oil (19.7 mg, 65%).

**R_f_** (SiO_2_, PE/Tol, 1:3): 0.60; **^1^H NMR** (400 MHz, CDCl_3_): δ = 7.51-7.55 (m, 1H), 7.43-7.48 (m, 2H), 7.36-7.41 (m, 1H), 7.09-7.13 (m, 1H), 6.98-7.05 (m, 3H), 5.59-5.70 (m, 1H), 5.16-5.21 (m, 2H), 3.04 (dd, *J* = 14.1, 6.7 Hz, 1H), 2.94 (dd, *J* = 14.1, 7.5 Hz, 1H) ppm. **^13^C NMR** (101 MHz, CDCl_3_): δ = 163.83 (s, d: *J*_C-F_ = 251.5 Hz, 1C), 162.78 (s, d: *J*_C-F_ = 249.5 Hz, 1C), 139.17 (d, 1C), 133.14 (d, d: *J*_C-F_ = 8.1 Hz, 1C), 131.40 (s, d: *J*_C-F_ = 3.3 Hz, 1C), 130.45 (d, 1C), 128.97 (d, d: *J*_C-F_ = 8.5 Hz, 2C), 124.88 (d, d: *J*_C-F_ = 3.9 Hz, 1C), 121.38 (t, 1C), 119.31 (s, 1C), 116.70 (s, d: *J*_C-F_ = 18.1 Hz, 1C), 116.25 (d, d: *J*_C-F_ = 23.5 Hz, 1C), 115.74 (d, d: *J*_C-F_ = 21.8 Hz, 2C), 53.58 (s, 1C), 44.57 (t, 1C) ppm. **^19^F NMR** (283 MHz, CDCl_3_): δ **=** -104.62 (s, 1F), -112.45 (s, 1F) ppm. **IR** (Reflexion)**:** ṽ = 3079, 2925, 2234, 1732, 1642, 1600, 1572, 1510, 1474, 1445, 1413, 1263, 1236, 1165, 1124, 1106, 1070, 1015, 994, 930, 822, 791, 759, 722 cm^-1^. **HR-MS** (EI(+)): *m/z* = 301.0720, calcd. for [M]^+^ = [C_17_H_13_F_2_NS]^+^: 301.0731.

**2-(4-Fluorophenyl)-2-(o-tolylthio)pent-4-enenitrile (3ai)**

The product was prepared according to **GP4**, using 74.2 mg of **1a** (0.30 mmol, 1.00 eq.) and 493 mg of **2i** (3.00 mmol, 10.0 eq.) in 6.0 mL of DCM. Purification by flash column chromatography gave the product as a yellow oil (59.5 mg, 67%).

**R_f_** (SiO_2_, PE/Et_2_O, 15:1): 0.43; **^1^H NMR** (300 MHz, CDCl_3_): δ = 7.31-7.40 (m, 3H), 7.18-7.28 (m, 2H), 7.05-7.10 (m, 1H), 6.96-7.04 (m, 2H), 5.61-5.74 (m, 1H), 5.15-5.23 (m, 2H), 2.94-3.07 (m, 2H), 2.32 (s, 3H) ppm. **^13^C NMR** (101 MHz, CDCl_3_): δ = 162.67 (s, d, *J*_C-F_ = 249.0 Hz, 1C), 144.21 (s, 1C), 138.01 (d, 1C), 132.07 (s, d: *J*_C-F_ = 3.4 Hz, 1C), 130.92 (d, 1C), 130.67 (d, 1C), 130.65 (d, 1C), 128.92 (d, d: *J*_C-F_ = 8.4 Hz, 2C), 128.76 (s, 1C), 126.57 (d, 1C), 121.24 (t, 1C), 119.48 (s, 1C), 115.71 (d, d: *J*_C-F_ = 21.8 Hz, 2C), 53.17 (s, 1C), 44.18 (t, 1C), 21.06 (q, 1C) ppm. **^19^F NMR** (283 MHz, CDCl_3_): δ **=** -112.74 (s, 1F) ppm. **IR** (Reflexion)**:** ṽ = 3080, 3062, 3013, 2982, 2925, 2855, 2233, 1641, 1601, 1509, 1469, 1413, 1380, 1306, 1237, 1164, 1130, 1106, 1060, 1015, 993, 928, 843, 816, 789, 756, 712 cm^-1^. **HR-MS** (EI(+)): *m/z* = 297.0947, calcd. for [M]^+^ = [C_18_H_16_FNS]^+^: 297.0982.

**2-(4-Fluorophenyl)-2-((2-methoxyphenyl)thio)pent-4-enenitrile (3aj)**

The product was prepared according to **GP4**, using 74.2 mg of **1a** (0.30 mmol, 1.00 eq.) and 505 mg of **2j** (3.00 mmol, 10.0 eq.) in 6 mL of DCM. Purification by flash column chromatography gave the product as a yellow oil (48.3 mg, 51%).

**R_f_** (SiO_2_, PE/Et_2_O, 5:1): 0.38; **^1^H NMR** (500 MHz, CDCl_3_): δ = 7.40-7.45 (m, 3H), 7.32-7.36 (m, 1H), 6.95-6.99 (m, 2H), 6.87 (td, *J* = 7.5, 1.2 Hz, 1H), 6.79 (dd, *J* = 8.3, 1.2 Hz, 1H), 5.62-5.71 (m, 1H), 5.15-5.20 (m, 2H), 3.69 (s, 3H), 3.02 (dd, *J* = 14.2, 6.9 Hz, 1H), 2.95 (dd, *J* = 14.1, 7.4 Hz, 1H) ppm. **^13^C NMR** (126 MHz, CDCl_3_): δ = 162.50 (s, d: *J*_C-F_ = 248.4 Hz, 1C), 160.82 (s, 1C), 139.08 (d, 1C), 132.48 (d, 1C), 131.97 (s, d: *J*_C-F_ = 3.4 Hz, 1C), 130.86 (d, 1C), 129.01 (d, d: *J*_C-F_ = 8.3 Hz, 2C), 121.08 (t, 1C), 121.06 (d, 1C), 119.67 (s, 1C), 117.48 (s, 1C), 115.32 (d, d: *J*_C-F_ = 21.9 Hz, 2C), 111.14 (d, 1C), 55.59 (q, 1C), 53.16 (s, 1C), 44.55 (t, 1C) ppm. **^19^F NMR** (283 MHz, CDCl_3_): δ **=** -113.29 (s, 1F) ppm. **IR** (Reflexion)**:** ṽ = 3072, 3009, 2936, 2837, 2232, 2045, 1890, 1642, 1600, 1583, 1509, 1476, 1432, 1412, 1276, 1249, 1182, 1164, 1131, 1105, 1067, 1042, 1025, 994, 929, 843, 817, 797, 756, 687 cm^-1^. **HR-MS** (EI(+)): *m/z* = 313.0945, calcd. for [M]^+^ = [C_18_H_16_FNOS]^+^: 313.0931.

**2-((2,6-Dimethylphenyl)thio)-2-(4-fluorophenyl)pent-4-enenitrile (3ak)**

The product was prepared according to **GP4**, using 74.2 mg of **1a** (0.30 mmol, 1.00 eq.) and 535 mg of **2k** (3.00 mmol, 10.0 eq.) in 6 mL of DCM. Purification by flash column chromatography gave the product as a yellow oil (51.4 mg, 55%).

**R_f_** (SiO_2_, PE/Et_2_O, 15:1): 0.42; **^1^H NMR** (300 MHz, CDCl_3_): δ = 7.33-7.40 (m, 2H), 7.16-7.21 (m, 1H), 7.08-7.11 (m, 2H), 6.98-7.06 (m, 2H), 5.64-5.78 (m, 1H), 5.17-5.25 (m, 2H), 2.98-3.12 (m, 2H), 2.34 (s, 6H) ppm. **^13^C NMR** (126 MHz, CDCl_3_): δ = 162.73 (s, d: *J*_C-F_ = 249.1 Hz, 1C), 146.18 (s, 2C), 132.66 (s, d: *J*_C-F_ = 3.4 Hz, 1C), 130.91 (d, 1C), 130.55 (d, 1C), 128.74 (d, *J*_C-F_ = 8.6 Hz, 2C), 128.67 (d, 2C), 128.40 (s, 1C), 121.22 (t, 1C), 119.09 (s, 1C), 115.76 (d, d: *J*_C-F_ = 21.9 Hz, 2C), 53.00 (s, 1C), 44.20 (t, 1C), 22.43 (q, 2C) ppm. **^19^F NMR** (283 MHz, CDCl_3_): δ **=** -112.83 (s, 1F) ppm. **IR** (Reflexion)**:** ṽ = 3058, 2981, 2926, 2233, 1642, 1601, 1509, 1459, 1377, 1237, 1165, 1106, 1015, 993, 927, 843, 816, 775 cm^-1^. **HR-MS** (EI(+)): *m/z* = 311.1141, calcd. for [M]^+^ = [C_19_H_18_FNS]^+^: 311.1139.

**2-((3-Chlorophenyl)thio)-2-(4-fluorophenyl)pent-4-enenitrile (3al)**

The product was prepared according to **GP4**, using 74.2 mg of **1a** (0.30 mmol, 1.00 eq.) and 554 mg of **2l** (3.00 mmol, 10.0 eq.) in 6 mL of DCM. Purification by flash column chromatography gave the product as a yellow oil (66.4 mg, 70%).

**R_f_** (SiO_2_, PE/Et_2_O, 15:1): 0.40; **^1^H NMR** (500 MHz, CDCl_3_): δ = 7.38-7.41 (m, 2H), 7.33-7.35 (m, 1H), 7.26-7.29 (m, 2H), 7.19-7.22 (m, 1H), 7.01-7.05 (m, 2H), 5.62-5.70 (m, 1H), 5.18-5.22 (m, 2H), 2.92-3.01 (m, 2H) ppm. **^13^C NMR** (126 MHz, CDCl_3_): δ = 162.69 (s, d: *J*_C-F_ = 249.3 Hz, 1C), 136.29 (d, 1C), 134.72 (d, 1C), 134.59 (s, 1C), 131.58 (s, d: *J*_C-F_ = 3.4 Hz, 1C), 131.17 (s, 1C), 130.67 (d, 1C), 130.28 (d, 1C), 130.19 (d, 1C), 128.93 (d, d: *J*_C-F_ = 8.6 Hz, 2C), 121.51 (t, 1C), 119.17 (s, 1C), 115.86 (d, d: *J*_C-F_ = 22.0 Hz, 2C), 53.69 (s, 1C), 44.49 (t, 1C) ppm. **^19^F NMR** (283 MHz, CDCl_3_): δ **=** -112.28 (s, 1F) ppm. **IR** (Reflexion)**:** ṽ = 3081, 3013, 2983, 2925, 2855, 2233, 1890, 1642, 1601, 1563, 1509, 1461, 1439, 1399, 1291, 1237, 1164, 1114, 1084, 1072, 1015, 996, 930, 841, 817, 783, 720, 683, 664 cm^-1^. **HR-MS** (EI(+)): *m/z* = 317.0434, calcd. for [M]^+^ = [C_17_H_13_ClFNS]^+^: 317.0436.

**2-((3,5-Dimethylphenyl)thio)-2-(4-fluorophenyl)pent-4-enenitrile (3am)**

The product was prepared according to **GP4**, using 74.2 mg of **1a** (0.30 mmol, 1.00 eq.) and 535 mg of **2m** (3.00 mmol, 10.0 eq.) in 6 mL of DCM. Purification by flash column chromatography gave the product as a yellow oil (53.6 mg, 57%).

**R_f_** (SiO_2_, PE/Et_2_O, 15:1): 0.43; **^1^H NMR** (400 MHz, CDCl_3_): δ = 7.39-7.44 (m, 2H), 6.97-7.04 (m, 5H), 5.62-5.72 (m, 1H), 5.15-5.20 (m, 2H), 2.89-3.00 (m, 2H), 2.23 (s, 6H) ppm. **^13^C NMR** (101 MHz, CDCl_3_): δ = 162.61 (s, d: *J*_C-F_ = 248.7 Hz, 1C), 138.75 (s, 2C), 134.41 (d, 2C), 132.22 (d, 1C), 132.16 (s, d: *J*_C-F_ = 3.3 Hz, 1C), 130.75 (d, 1C), 129.07 (d, d: *J*_C-F_ = 8.4 Hz, 2C), 128.90 (s, 1C), 121.07 (t, 1C), 119.54 (s, 1C), 115.56 (d, d: *J*_C-F_ = 21.9 Hz, 2C), 53.32 (s, 1C), 44.44 (t, 1C), 21.13 (q, 2C) ppm. **^19^F NMR** (283 MHz, CDCl_3_): δ **=** -113.09 (s, 1F) ppm. **IR** (Reflexion)**:** ṽ = 3081, 3045, 3004, 2982, 2953, 2920, 2860, 2734, 2232, 1891, 1642, 1601, 1580, 1509, 1441, 1413, 1378, 1305, 1237, 1164, 1105, 1041, 1015, 994, 928, 851, 817, 791, 720, 689 cm^-1^. **HR-MS** (EI(+)): *m/z* = 311.1164, calcd. for [M]^+^ = [C_19_H_18_FNS]^+^: 311.1139.

**2-(4-Fluorophenyl)-2-(naphthalen-1-ylthio)pent-4-enenitrile (3an)**

The product was prepared according to **GP4**, using 74.2 mg of **1a** (0.30 mmol, 1.00 eq.) and 601 mg of **2n** (3.00 mmol, 10.0 eq.) in 6 mL of DCM. Purification by flash column chromatography gave the product as a yellow oil (54.7 mg, 55%).

**R_f_** (SiO_2_, PE/Tol, 1:3): 0.53; **^1^H NMR** (500 MHz, CDCl_3_): δ = 7.95 (d, *J* = 1.7 Hz, 1H), 7.81 (d, *J* = 7.8 Hz, 1H), 7.78 (d, *J* = 7.8 Hz, 1H), 7.72 (d, *J* = 8.5 Hz, 1H), 7.49-7.55 (m, 2H), 7.42-7.46 (m, 2H), 7.36 (dd, *J* = 8.6, 1.8 Hz, 1H), 6.98-7.02 (m, 2H), 5.64-5.72 (m, 1H), 5.17-5.22 (m, 2H), 2.96-3.05 (m, 2H) ppm. **^13^C NMR** (126 MHz, CDCl_3_): δ = 162.61 (s, d: *J*_C-F_ = 249.1 Hz, 1C), 137.50 (d, 1C), 133.82 (s, 1C), 133.41 (s, 1C), 132.48 (d, 1C), 131.94 (s, d: *J*_C-F_ = 3.4 Hz, 1C), 130.56 (d, 1C), 128.99 (d, d: *J*_C-F_ = 8.1 Hz, 2C), 128.72 (d, 1C), 128.34 (d, 1C), 127.77 (d, 1C), 127.68 (d, 1C), 126.74 (d, 1C), 126.70 (s, 1C), 121.27 (t, 1C), 119.53 (s, 1C), 115.74 (d, d: *J*_C-F_ = 21.9 Hz, 2C), 53.65 (s, 1C), 44.55 (t, 1C) ppm. **^19^F NMR** (283 MHz, CDCl_3_): δ **=** -112.72 (s, 1F) ppm. **IR** (Reflexion)**:** ṽ = 3056, 2925, 2854, 2232, 1731, 1644, 1600, 1508, 1439, 1412, 1235, 1164, 1132, 1105, 1015, 993, 927, 898, 859, 816, 746, 691 cm^-1^. **HR-MS** (EI(+)): *m/z* = 333.0992, calcd. for [M]^+^ = [C_21_H_16_FNS]^+^: 333.0982.

**2-(4-Fluorophenyl)-2-(pyridin-2-ylthio)pent-4-enenitrile (3ao)**

The product was prepared according to **GP4**, using 49.4 mg of **1a** (0.20 mmol, 1.00 eq.) and 303 mg of **2o** (2.00 mmol, 10.0 eq.) in 4 mL of DCM. Purification by flash column chromatography gave the product as a yellow oil (30.8 mg, 54%).

**R_f_** (SiO_2_, PE/EA, 5:1): 0.26; **^1^H NMR** (500 MHz, CDCl_3_): δ = 8.32-8.33 (m, 1H), 7.49-7.53 (m, 2H), 7.43 (td, J = 7.8, 1.9 Hz, 1H), 7.17-7.19 (m, 1H), 6.98-7.01 (m, 1H), 6.90-6.95 (m, 2H), 5.54-5.62 (m, 1H), 5.06-5.10 (m, 2H), 3.06 (dd, *J* = 14.1, 7.5 Hz, 1H), 2.87 (dd, *J* = 14.0, 6.9 Hz, 1H) ppm. **^13^C NMR** (126 MHz, CDCl_3_): δ = 162.54 (s, d: *J*_C-F_ = 248.6 Hz, 1C), 154.40 (s, 1C), 150.05 (d, 1C), 136.86 (d, 1C), 132.06 (s, d: *J*_C-F_ = 3.4 Hz, 1C), 130.29 (d, 1C), 128.85 (d, d: *J*_C-F_ = 8.4 Hz, 2C), 126.06 (d, 1C), 122.24 (d, 1C), 121.49 (t, 1C), 119.63 (s, 1C), 115.72 (d, d: *J*_C-F_ = 21.8 Hz, 2C), 51.47 (s, 1C), 45.81 (t, 1C) ppm. **^19^F NMR** (283 MHz, CDCl_3_): δ **=** -112.97 (s, 1F) ppm. **IR** (Reflexion)**:** ṽ = 3077, 2960, 2926, 2235, 1732, 1640, 1601, 1574, 1561, 1508, 1452, 1416, 1235, 1164, 1123, 1044, 1015, 988, 929, 821, 760, 725, 619 cm^-1^. **HR-MS** (EI(+)): *m/z* = 284.0766, calcd. for [M]^+^ = [C_16_H_13_FN_2_S]^+^: 284.0783.

**2-(4-Fluorophenyl)-2-(thiophen-2-ylthio)pent-4-enenitrile (3ap)**

The product was prepared according to **GP4**, using 74.2 mg of **1a** (0.30 mmol, 1.00 eq.) and 469 mg of **2p** (3.00 mmol, 10.0 eq.) in 6 mL of DCM. Purification by flash column chromatography gave the product as a yellow oil (32.2 mg, 37%).

**R_f_** (SiO_2_, PE/Tol, 1:3): 0.61; **^1^H NMR** (500 MHz, CDCl_3_): δ = 7.42-7.45 (m, 3H), 7.21 (d, *J* = 3.6 Hz, 1H), 7.00-7.06 (m, 3H), 5.62-5.70 (m, 1H), 5.17-5.21 (m, 2H), 2.96 (d, *J* = 7.2 Hz, 2H) ppm. **^13^C NMR** (126 MHz, CDCl_3_): δ = 162.81 (s, d: *J*_C-F_ = 249.1 Hz, 1C), 138.68 (d, 1C), 133.45 (d, 1C), 131.45 (s, d: *J*_C-F_ = 3.4 Hz, 1C), 130.42 (d, 1C), 129.02 (d, d: *J*_C-F_ = 8.2 Hz, 2C), 128.08 (d, 1C), 127.33 (s, 1C), 121.38 (t, 1C), 119.37 (s, 1C), 115.91 (d, d: *J*_C-F_ = 22.0 Hz, 2C), 55.26 (s, 1C), 43.91 (t, 1C) ppm. **^19^F NMR** (283 MHz, CDCl_3_): δ **=** -112.38 (s, 1F) ppm. **IR** (Reflexion)**:** ṽ = 3082, 2926, 2233, 1601, 1509, 1399, 1238, 1165, 1106, 993, 932, 851, 789, 716 cm^-1^. **HR-MS** (EI(+)): *m/z* = 289.0370, calcd. for [M]^+^ = [C_15_H_12_FNS_2_]^+^: 289.0390.

**2-(Benzylthio)-2-(4-fluorophenyl)pent-4-enenitrile (3aq)**

The product was prepared according to **GP4**, using 74.2 mg of **1a** (0.30 mmol, 1.0 eq.) and 493 mg of **2q** (3.00 mmol, 10.0 eq.) in 6 mL of DCM. Purification by flash column chromatography gave the product as a yellow oil (60.5 mg, 68%).

**R_f_** (SiO_2_, PE/Et_2_O, 15:1): 0.46; **^1^H NMR** (300 MHz, CDCl_3_): δ = 7.55-7.62 (m, 2H), 7.20-7.27 (m, 3H), 7.13-7.17 (m, 2H), 7.03-7.11 (m, 2H), 5.58-5.72 (m, 1H), 5.11-5.19 (m, 2H), 3.89 (d, *J* = 11.8 Hz, 1H), 3.53 (d, *J* = 11.8 Hz, 1H), 2.74-2.90 (m, 2H) ppm. **^13^C NMR** (101 MHz, CDCl_3_): δ = 162.65 (s, d: *J*_C-F_ = 248.8 Hz, 1C), 135.28 (s, 1C), 132.37 (s, d: *J*_C-F_ = 3.4 Hz, 1C), 130.38 (d, 1C), 129.35 (d, 2C), 128.74 (d, 2C), 128.70 (d, d: *J*_C-F_ = 7.9 Hz, 2C), 127.69 (d, 1C), 121.30 (t, 1C), 119.44 (s, 1C), 116.00 (d, d: *J*_C-F_ = 21.9 Hz, 2C), 50.91 (s, 1C), 45.95 (t, 1C), 36.26 (t, 1C) ppm. **^19^F NMR** (283 MHz, CDCl_3_): δ **=** -112.80 ppm. **IR** (Reflexion)**:** ṽ = 3083, 3064, 3030, 2982, 2923, 2849, 2232, 1641, 1600, 1507, 1454, 1436, 1412, 1304, 1234, 1163, 1105, 1070, 1029, 1014, 993, 928, 819, 801, 696 cm^-1^. **HR-MS** (EI(+)): *m/z* = 297.1001, calcd. for [M]^+^ = [C_18_H_16_FNS]^+^: 297.0982.

**2-(4-Fluorophenyl)-2-(propylthio)pent-4-enenitrile (3ar)**

The product was prepared according to **GP4**, using 49.4 mg of **1a** (0.20 mmol, 1.00 eq.) and 232 mg of **2r** (2.00 mmol, 10.0 eq.) in 4 mL of DCM. Purification by flash column chromatography gave the product as a yellow oil (36.8 mg, 74%).

**R_f_** (SiO_2_, PE/Et_2_O, 30:1): 0.50; ^1^H NMR (500 MHz, CDCl_3_): δ = 7.56-7.60 (m, 2H), 7.06-7.11 (m, 2H), 5.60-5.69 (m, 1H), 5.13-5.18 (m, 2H), 2.86 (dd, *J* = 14.0, 7.5 Hz, 1H), 2.77 (dd, *J* = 14.0, 6.8 Hz, 1H), 2.59-2.64 (m, 1H), 2.28-2.34 (m, 1H), 1.57 (m, 2H), 0.91 (t, *J* = 7.3 Hz, 3H) ppm. **^13^C NMR** (126 MHz, CDCl_3_): δ = 162.58 (s, d: *J*_C-F_ = 249.1 Hz, 1C), 132.79 (s, d: *J*_C-F_ = 3.0 Hz, 1C), 130.58 (d, 1C), 128.65 (d, d: *J*_C-F_ = 8.6 Hz, 2C), 121.10 (t, 1C),119.84 (s, 1C), 115.92 (d, d: *J*_C-F_ = 21.9 Hz, 2C), 50.19 (s, 1C), 46.13 (t, 1C), 33.45 (t, 1C), 21.74 (t, 1C), 13.62 (q, 1C) ppm. **^19^F NMR** (283 MHz, CDCl_3_): δ **=** -113.05 (s, 1F) ppm. **IR** (Reflexion)**:** ṽ = 3082, 2965, 2932, 2874, 2232, 1895, 1642, 1601, 1509, 1459, 1437, 1413, 1380, 1303, 1236, 1164, 1105, 1015, 994, 929, 822, 804, 723, 693 cm^-1^. **HR-MS** (EI(+)): *m/z* = 249.0953, calcd. for [M]^+^ = [C_14_H_16_FNS]^+^: 249.0982.

**2-(Cyclohexylthio)-2-(4-fluorophenyl)pent-4-enenitrile (3as)**

The product was prepared according to **GP4**, using 74.2 mg of **1a** (0.30 mmol, 1.00 eq.) and 469 mg of **2s** (3.00 mmol, 10.0 eq.) in 6 mL of DCM. Purification by flash column chromatography gave the product as a yellow oil (59.9 mg, 69%).

**R_f_** (SiO_2_, PE/Et_2_O, 15:1): 0.56; **^1^H NMR** (300 MHz, CDCl_3_): δ = 7.56-7.63 (m, 2H), 7.04-7.12 (m, 2H), 5.55-5.69 (m, 1H), 5.09-5.17 (m, 2H), 2.85 (dd, *J* = 13.9, 7.4 Hz, 1H), 2.74 (dd, *J* = 14.0, 6.9 Hz, 1H), 2.60-2.68 (m, 1H), 2.03-2.11 (m, 1H), 1.66-1.73 (m, 1H), 1.44-1.58 (m, 4H), 1.09-1.31 (m, 4H) ppm. **^13^C NMR** (101 MHz, CDCl_3_): δ = 162.54 (s, d: *J*_C-F_ = 248.5 Hz, 1C), 133.28 (s, d: *J*_C-F_ = 3.3 Hz, 1C), 130.55 (d, 1C), 128.68 (d, d: *J*_C-F_ = 8.3 Hz, 2C), 121.11 (t, 1C), 120.31 (s, 1C), 115.85 (d, d: *J*_C-F_ = 21.8 Hz, 2C), 49.64 (s, 1C), 46.76 (t, 1C), 44.97 (d, 1C), 34.10 (t, 1C), 33.81 (t, 1C), 25.86 (t, 1C), 25.80 (t, 1C), 25.49 (t, 1C) ppm. **^19^F NMR** (283 MHz, CDCl_3_): δ **=** -113.12 (s, 1F) ppm. **IR** (Reflexion)**:** ṽ = 3081, 2932, 2854, 2232, 1642, 1601, 1508, 1448, 1412, 1342, 1304, 1264, 1236, 1163, 1104, 1014, 996, 928, 886, 821, 803, 740, 697 cm^-1^. **HR-MS** (EI(+)): *m/z* = 289.1291, calcd. for [M]^+^ = [C_17_H_20_FNS]^+^: 289.1295.

**2-(Tert-butylthio)-2-(4-fluorophenyl)pent-4-enenitrile (3at)**

The product was prepared according to **GP4**, using 74.2 mg of **1a** (0.30 mmol, 1.00 eq.) and 391 mg of **2t** (3.00 mmol, 10.0 eq.) in 6 mL of DCM. Purification by flash column chromatography gave the product as a yellow oil (52.7 mg, 67%).

**R_f_** (SiO_2_, PE/Et_2_O, 30:1): 0.48; **^1^H NMR** (500 MHz, CDCl_3_): δ = 7.62-7.65 (m, 2H), 7.05-7.09 (m, 2H), 5.54-5.62 (m, 1H), 5.10-5.15 (m, 2H), 2.85 (dd, *J* = 13.9, 7.5 Hz, 1H), 2.71 (dd, *J* = 13.9, 6.8 Hz, 1H), 1.21 (s, 9H) ppm. **^13^C NMR** (126 MHz, CDCl_3_): δ = 162.55 (s, d: *J*_C-F_ = 248.5 Hz, 1C), 133.78 (s, d: *J*_C-F_ = 3.4 Hz, 1C), 130.17 (d, 1C), 128.83 (d, d: *J*_C-F_ = 8.1 Hz, 2C), 121.23 (t, 1C), 120.69 (s, 1C), 115.74 (d, d: *J*_C-F_ = 21.9 Hz, 2C), 48.72 (s, 1C), 48.60 (s, 1C), 47.91 (t, 1C), 31.74 (q, 3C) ppm. **^19^F NMR** (283 MHz, CDCl_3_): δ **=** -113.34 (s, 1F) ppm. **IR** (Reflexion)**:** ṽ = 3082, 2965, 2926, 2863, 2233, 1646, 1602, 1509, 1459, 1440, 1412, 1396, 1367, 1305, 1230, 1162, 1105, 1015, 993, 929, 820, 803, 724, 692 cm^-1^. **HR-MS** (EI(+)): *m/z* = 263.1119, calcd. for [M]^+^ = [C_15_H_18_FNS]^+^: 263.1139.

**2-(4-Fluorophenyl)-3-methyl-2-(phenylthio)pent-4-enenitrile (3au)**

The product was prepared according to **GP4**, using 24.7 mg of **1a** (0.10 mmol, 1.00 eq.) and 164 mg of **2u** (1.00 mmol, 10.0 eq.) in 2 mL of DCM. Purification by flash column chromatography gave the product as a mixture of diastereoisomers (14.5 mg, 49%, d.r. 1:1.1).

**R_f_** (SiO_2_, PE/EA, 20:1): 0.37; **^1^H NMR** (400 MHz, CDCl_3_): δ = 7.29-7.35 (both isomers, m, 4H), 7.12-7.29 (both isomers, m, 10H), 6.90-6.96 (both isomers, m, 4H), 6.06-6.15 (main isomer, m, 1H), 5.50-5.59 (minor isomer, m, 1H), 5.31-5.39 (main isomer, m, 2H), 4.94-5.00 (minor isomer, m, 2H), 3.04-3.10 (minor isomer, m, 1H), 2.96-3.03 (main isomer, m, 1H), 1.51 (minor isomer, d, *J* = 6.7 Hz, 3H), 1.01 (main isomer, d, *J* = 6.8 Hz, 3H) ppm. **^13^C NMR** (101 MHz, CDCl_3_): δ = 162.36 (main isomer, s, d: *J*_C-F_ = 248.7 Hz, 1C), 162.29 (minor isomer, s, d: *J*_C-F_ = 248.4 Hz, 1C), 137.39 (main isomer, d, 1C), 136.74 (minor isomer, d, 1C), 136.67 (minor isomer, d, 2C), 136.55 (main isomer, d, 2C), 132.37 (minor isomer, s, d: *J*_C-F_ = 3.3 Hz, 1C), 132.19 (main isomer, s, d: *J*_C-F_ = 3.3 Hz, 1C), 130.15 (minor isomer, d, 1C), 130.02 (main isomer, d, 1C), 129.85 (minor isomer, s, 1C), 129.47 (minor isomer, d, d: *J*_C-F_ = 8.2 Hz, 2C), 129.45 (main isomer, s, 1C), 129.18 (main isomer, d, d: *J*_C-F_ = 8.3 Hz, 2C), 128.98 (minor isomer, d, 2C), 128.91 (main isomer, d, 2C), 118.78 (both isomers, s, 2C), 118.57 (main isomer, t, 1C), 118.24 (minor isomer, t, 1C), 115.52 (main isomer, d, d: *J*_C-F_ = 21.8 Hz, 2C), 115.28 (minor isomer, d, d: *J*_C-F_ = 21.9 Hz, 2C), 59.46 (minor isomer, s, 1C), 58.99 (main isomer, s, 1C), 47.95 (main isomer, d, 1C), 46.91 (minor isomer, d, 1C), 17.63 (main isomer, q, 1C), 17.15 (minor isomer, q, 1C) ppm. **^19^F NMR** (283 MHz, CDCl_3_): δ **=** -113.14 (main isomer, s, 1F), -113.33 (minor isomer, s, 1F) ppm. **IR** (Reflexion)**:** ṽ = 3078, 2978, 2933, 2874, 2231, 1890, 1639, 1602, 1510, 1477, 1455, 1440, 1412, 1377, 1305, 1234, 1164, 1123, 1069, 1015, 995, 928, 850, 811, 748, 721, 691 cm^-1^. **HR-MS** (EI(+)): *m/z* = 297.0980, calcd. for [M]^+^ = [C_18_H_16_FNS]^+^: 297.0982.

**2-(4-Fluorophenyl)-3-phenyl-2-(phenylthio)pent-4-enenitrile (3av)**

The product was prepared according to **GP4**, using 24.7 mg of **1a** (0.10 mmol, 1.00 eq.) and 226 mg of **2v** (1.00 mmol, 10.0 eq.) in 2 mL of DCM. Purification by flash column chromatography gave the product as a mixture of diastereoisomers (20.5 mg, 57%, d.r. 1:1.6).

**R_f_** (SiO_2_, PE/Tol, 1:3): 0.63; **^1^H NMR** (500 MHz, CDCl_3_): δ = 7.34-7.46 (both isomers, m, 12H), 7.02-7.25 (both isomers, m, 21H), 6.94 (main isomer, t, *J* = 8.5 Hz, 3H), 6.79 (minor isomer, t, *J* = 8.5 Hz, 2H), 6.56-6.64 (minor isomer, m, 1H), 6.05-6.12 (main isomer, m, 1H), 5.39-5.43 (minor isomer, m, 2H), 5.08 (main isomer, d, *J* = 10.2 Hz, 1H), 4.90 (main isomer, d, *J* = 16.8 Hz, 1H), 4.05 (main isomer, d, *J* = 8.5 Hz, 1H), 4.00 (minor isomer, d, *J* = 9.2 Hz, 1H) ppm. **^13^C NMR** (126 MHz, CDCl_3_): δ = 162.41 (main isomer, s, d, *J*_C-F_ = 249.1 Hz, 1C), 162.17 (minor isomer, s, d, *J*_C-F_ = 248.6 Hz, 1C), 138.05 (minor isomer, s, 1C), 137.41 (main isomer, s, 1C), 136.44 (both isomers, d, 4C), 135.33 (minor isomer, d, 1C), 134.60 (main isomer, d, 1C), 131.97 (main isomer, s, d: *J*_C-F_ = 3.4 Hz, 1C), 131.64 (minor isomer, s, d: *J*_C-F_ = 2.9 Hz, 1C), 130.07 (minor isomer, d, 1C), 130.00 (main isomer, d, 1C), 129.87 (main isomer, d, d: *J*_C-F_ = 8.5 Hz, 2C), 129.72 (main isomer, s, 1C), 129.70 (minor isomer, d, d: *J*_C-F_ = 8.0 Hz, 2C), 129.67 (minor isomer, s, 1C), 129.60 (main isomer, d, 2C), 128.96 (minor isomer, d, 2C), 128.91 (main isomer, d, 2C), 128.83 (minor isomer, d, 2C), 128.69 (main isomer, d, 2C), 128.52 (main isomer, d, 1C), 128.45 (minor isomer, d, 2C), 127.66 (minor isomer, d, 1C), 120.12 (main isomer, t, 1C), 120.07 (minor isomer, t, 1C), 119.28 (main isomer, s, 1C), 119.18 (minor isomer, s, 1C), 115.36 (main isomer, d, d: *J*_C-F_ = 22.1 Hz, 2C), 115.18 (minor isomer, d, d: *J*_C-F_ = 22.5 Hz, 2C), 59.51 (minor isomer, d, 1C), 59.26 (main isomer, d, 1C), 59.06 (main isomer, s, 1C), 59.03 (minor isomer, s, 1C) ppm. **^19^F NMR** (283 MHz, CDCl_3_): δ **=** -113.03 (main isomer, s, 1F), -113.22 (minor isomer, s, 1F) ppm. **IR** (Reflexion)**:** ṽ = 3062, 3032, 2981, 2926, 2230, 1955, 1890, 1807, 1732, 1637, 1601, 1509, 1476, 1455, 1439, 1411, 1306, 1272, 1237, 1164, 1134, 1107, 1070, 1025, 1015, 989, 930, 840, 810, 787, 750, 729, 705, 666 cm^-1^. **HR-MS** (EI(+)): *m/z* = 359.1119, calcd. for [M]^+^ = [C_23_H_18_FNS]^+^: 359.1139.

**2-(4-Fluorophenyl)-3,3-dimethyl-2-(phenylthio)pent-4-enenitrile (3aw)**

The product was prepared according to **GP4**, using 49.4 mg of **1a** (0.20 mmol, 1.00 eq.) and 357 mg of **2w** (2.00 mmol, 10.0 eq.) in 4 mL of DCM. Purification by flash column chromatography gave the product as a yellow oil (29.3 mg, 47%).

**R_f_** (SiO_2_, PE/Et_2_O, 15:1): 0.58; **^1^H NMR** (500 MHz, CDCl_3_): δ = 7.58-7.62 (m, 2H), 7.30-7.33 (m, 2H), 7.23-7.27 (m, 1H), 7.14-7.18 (m, 2H), 6.95-6.99 (m, 2H), 6.06 (dd, *J* = 17.3, 10.7 Hz, 1H), 5.23 (d, *J* = 10.7 Hz, 1H), 5.09 (d, *J* = 17.2 Hz, 1H), 1.41 (s, 3H), 1.22 (s, 3H) ppm. **^13^C NMR** (126 MHz, CDCl_3_): δ = 162.48 (s, d: *J*_C-F_ = 248.6 Hz, 1C), 141.18 (d, 1C), 136.08 (d, 2C), 131.47 (d, d: *J*_C-F_ = 8.5 Hz, 2C), 130.31 (s, d: *J*_C-F_ = 3.4 Hz, 1C), 130.08 (s, 1C), 129.80 (d, 1C), 128.95 (d, 2C), 119.75 (s, 1C), 116.20 (t, 1C), 114.60 (d, d: *J*_C-F_ = 21.9 Hz, 2C), 64.03 (s, 1C), 45.44 (s, 1C), 25.00 (q, 1C), 23.87 (q, 1C) ppm. **^19^F NMR** (283 MHz, CDCl_3_): δ **=** -113.47 (s, 1F) ppm. **IR** (Reflexion)**:** ṽ = 3077, 3063, 2978, 2935, 2875, 2231, 1733, 1638, 1600, 1508, 1477, 1439, 1414, 1384, 1367, 1306, 1231, 1165, 1107, 1069, 1015, 1001, 925, 857, 828, 808, 783, 748, 690 cm^-1^. **HR-MS** (EI(+)): *m/z* = 311.1130, calcd. for [M]^+^ = [C_19_H_18_FNS]^+^: 311.1139.

**2-(4-Fluorophenyl)-2-(phenylthio)hex-4-enenitrile (3ax)**

The product was prepared according to **GP4**, using 49.4 mg of **1a** (0.20 mmol, 1.00 eq.) and 328 mg of **2x** (2.00 mmol, 10.0 eq.) in 4 mL of DCM. Purification by flash column chromatography gave the product as a yellow oil (38.6 mg, 65%, d.r. 1:6.7).

**R_f_** (SiO_2_, PE/Et_2_O, 15:1): 0.48; **^1^H NMR** (400 MHz, CDCl_3_): δ = 7.33-7.44 (main isomer, m, 5H), 7.23-7.28 (main isomer, m, 2H), 6.96-7.02 (main isomer, m, 2H), 5.56-5.69 (main isomer, m, 1H), 5.25-5.35 (main isomer, m, 1H), 2.84-2.92 (main isomer, m, 2H), 1.63 (main isomer, ddd, *J* = 6.5, 2.5, 1.1 Hz, 3H) ppm. **^13^C NMR** (101 MHz, CDCl_3_): δ = 162.55 (main isomer, s, d: *J*_C-F_ = 248.7 Hz, 1C), 136.79 (main isomer, d, 2C), 132.32 (main isomer, d, 1C), 132.27 (main isomer, s, d, *J*_C-F_ = 3.2 Hz, 1C), 130.36 (main isomer, d, 1C), 129.70 (main isomer, s, 1C), 129.12 (main isomer, d, 2C), 128.97 (main isomer, d, d: *J*_C-F_ = 8.3 Hz, 2C), 123.09 (main isomer, d, 1C), 119.71 (main isomer, s, 1C), 115.62 (main isomer, d, d: *J*_C-F_ = 21.7 Hz, 2C), 54.05 (main isomer, s, 1C), 43.60 (main isomer, t, 1C), 18.07 (main isomer, q, 1C) ppm. **^19^F NMR** (283 MHz, CDCl_3_): δ **=** -113.04 (main isomer, s, 1F) ppm. **IR** (Reflexion)**:** ṽ = 3060, 3029, 2968, 2940, 2918, 2855, 2232, 1891, 1670, 1602, 1509, 1475, 1439, 1411, 1306, 1238, 1164, 1106, 1069, 1025, 966, 840, 818, 750, 692, 643 cm^-1^. **HR-MS** (EI(+)): *m/z* = 297.0983, calcd. for [M]^+^ = [C_18_H_16_FNS]^+^: 297.0982.

**2-(4-Fluorophenyl)-2-(phenylthio)penta-3,4-dienenitrile (5a)**

The product was prepared according to **GP4**, using 49.4 mg of **1a** (0.20 mmol, 1.00 eq.) and 296 mg of **4a** (2.00 mmol, 10.0 eq.) in 4 mL of DCM. Purification by flash column chromatography gave the product as a yellow oil (35.2 mg, 63%).

**R_f_** (SiO_2_, PE/Tol, 1:3): 0.60; **^1^H NMR** (300 MHz, CDCl_3_): δ = 7.54-7.59 (m, 2H), 7.49-7.53 (m, 2H), 7.41-7.47 (m, 1H), 7.31-7.37 (m, 2H), 7.02-7.10 (m, 2H), 5.58 (t, *J* = 6.5 Hz, 1H), 5.03 (dd, *J* = 12.0, 6.5 Hz, 1H), 4.80 (dd, *J* = 12.0, 6.4 Hz, 1H) ppm. **^13^C NMR** (101 MHz, CDCl_3_): δ = 207.60 (s, 1C), 162.90 (s, d: *J*_C-F_ = 249.4 Hz, 1C), 137.48 (d, 2C), 132.06 (s, d: *J*_C-F_ = 3.2 Hz, 1C), 130.73 (d, 1C), 130.19 (s, 1C), 129.11 (d, 2C), 128.91 (d, d: *J*_C-F_ = 8.4 Hz, 2C), 118.23 (s, 1C), 115.93 (d, d: *J*_C-F_ = 21.9 Hz, 2C), 92.41 (d, 1C), 81.17 (t, 1C), 53.35 (s, 1C) ppm. **^19^F NMR** (283 MHz, CDCl_3_): δ **=** -112.11 (s, 1F) ppm. **IR** (Reflexion)**:** ṽ = 3063, 2927, 2854, 2237, 1953, 1729, 1643, 1600, 1507, 1475, 1439, 1411, 1305, 1237, 1164, 1103, 1068, 1015, 858, 787, 749, 691 cm^-1^. **HR-MS** (EI(+)): *m/z* = 280.0579, calcd. for [M-H]^+^ = [C_17_H_11_FNS]^+^: 280.0591.

**2-(4-Fluorophenyl)-2-((4-fluorophenyl)thio)penta-3,4-dienenitrile (5b)**

The product was prepared according to **GP4**, using 49.4 mg of **1a** (0.20 mmol, 1.00 eq.) and 332 mg of **4b** (2.00 mmol, 10.0 eq.) in 4 mL of DCM. Purification by flash column chromatography gave the product as a yellow solid (35.8 mg, 60%).

**R_f_** (SiO_2_, PE/Tol, 1:3): 0.58; **^1^H NMR** (300 MHz, CDCl_3_): δ = 7.44-7.58 (m, 4H), 7.00-7.11 (m, 4H), 5.58 (t, *J* = 6.5 Hz, 1H), 5.06 (dd, *J* = 12.1, 6.5 Hz, 1H), 4.85 (dd, *J* = 12.1, 6.5 Hz, 1H) ppm. **^13^C NMR** (101 MHz, CDCl_3_): δ = 207.68 (s, 1C), 164.67 (s, d: *J*_C-F_ = 252.1 Hz, 1C), 162.96 (s, d: *J*_C-F_ = 249.7 Hz, 1C), 139.67 (d, d: *J*_C-F_ = 8.9 Hz, 2C), 131.89 (s, d: *J*_C-F_ = 3.4 Hz, 1C), 128.90 (d, d: *J*_C-F_ = 8.5 Hz, 2C), 125.67 (s, d: *J*_C-F_ = 3.3 Hz, 1C), 118.04 (s, 1C), 116.36 (d, d: *J*_C-F_ = 22.0 Hz, 2C), 116.00 (d, d: *J*_C-F_ = 21.9 Hz, 2C), 92.30 (d, 1C), 81.28 (t, 1C), 53.59 (s, 1C) ppm. **^19^F NMR** (283 MHz, CDCl_3_): δ **=** -108.91 (s, 1F), -111.83 (s, 1F) ppm. **IR** (Reflexion)**:** ṽ = 3088, 2243, 1971, 1600, 1589, 1509, 1490, 1413, 1399, 1294, 1238, 1160, 1092, 1015, 939, 872, 832, 778, 693, 639 cm^-1^. **HR-MS** (EI(+)): *m/z* = 299.0549, calcd. for [M]^+^ = [C_17_H_11_F_2_NS]^+^: 299.0575; **m.p.** = 80.2-81.3 °C.

**2-(4-Fluorophenyl)-2-(p-tolylthio)penta-3,4-dienenitrile (5c)**

The product was prepared according to **GP4**, using 49.4 mg of **1a** (0.20 mmol, 1.00 eq.) and 325 mg of **4c** (2.00 mmol, 10.0 eq.) in 4 mL of DCM. Purification by flash column chromatography gave the product as a yellow oil (33.4 mg, 57%).

**R_f_** (SiO_2_, PE/Tol, 1:3): 0.64; **^1^H NMR** (300 MHz, CDCl_3_): δ = 7.52-7.59 (m, 2H), 7.36-7.39 (m, 2H), 7.13-7.16 (m, 2H), 7.03-7.10 (m, 2H), 5.57 (t, *J* = 6.5 Hz, 1H), 5.03 (dd, *J* = 11.9, 6.5 Hz, 1H), 4.82 (dd, *J* = 12.0, 6.5 Hz, 1H), 2.37 (s, 3H) ppm. **^13^C NMR** (75 MHz, CDCl_3_): δ = 207.64 (s, 1C), 162.88 (s, d: *J*_C-F_ = 249.3 Hz, 1C), 141.23 (s, 1C), 137.41 (d, 2C), 132.22 (s, d: *J*_C-F_ = 3.3 Hz, 1C), 129.90 (d, 2C), 128.94 (d, d: *J*_C-F_ = 8.4 Hz, 2C), 126.76 (s, 1C), 118.30 (s, 1C), 115.87 (d, d: *J*_C-F_ = 21.9 Hz, 2C), 92.54 (d, 1C), 81.11 (t, 1C), 53.37 (s, 1C), 21.53 (q, 1C) ppm. **^19^F NMR** (283 MHz, CDCl_3_): δ **=** -112.28 (s, 1F) ppm. **IR** (Reflexion)**:** ṽ = 3073, 3024, 2923, 2865, 2236, 1953, 1908, 1733, 1600, 1508, 1469, 1411, 1379, 1304, 1238, 1181, 1164, 1104, 1016, 968, 813, 720, 693 cm^-1^. **HR-MS** (EI(+)): *m/z* = 295.0825, calcd. for [M]^+^ = [C_18_H_14_FNS]^+^: 295.0826.

**2-(4-Fluorophenyl)-2-((2-fluorophenyl)thio)penta-3,4-dienenitrile (5d)**

The product was prepared according to **GP4**, using 49.4 mg of **1a** (0.20 mmol, 1.00 eq.) and 332 mg of **4d** (2.00 mmol, 10.0 eq.) in 4 mL of DCM. Purification by flash column chromatography gave the product as a yellow solid (32.7 mg, 55%).

**R_f_** (SiO_2_, PE/Et_2_O, 20:1): 0.44; **^1^H NMR** (500 MHz, CDCl_3_): δ = 7.61-7.68 (m, 3H), 7.44-7.49 (m, 1H), 7.16-7.19 (m, 1H), 7.06-7.13 (m, 3H), 5.63 (t, *J* = 6.4 Hz, 1H), 5.03 (dd, *J* = 12.0, 6.5 Hz, 1H), 4.77 (dd, *J* = 12.1, 6.4 Hz, 1H) ppm. **^13^C NMR** (126 MHz, CDCl_3_): δ = 207.43 (s, 1C), 164.16 (s, d: *J*_C-F_ = 250.8 Hz, 1C), 163.05 (s, d: *J*_C-F_ = 249.7 Hz, 1C), 139.70 (d, 1C), 133.45 (d, d: *J*_C-F_ = 8.1 Hz, 1C), 131.56 (s, d: *J*_C-F_ = 3.4 Hz, 1C), 128.90 (d, d: *J*_C-F_ = 8.7 Hz, 2C), 124.84 (d, d: *J*_C-F_ = 4.0 Hz, 1C), 118.00 (s, 1C), 117.47 (s, d: *J*_C-F_ = 17.9 Hz, 1C), 116.18 (d, d: *J*_C-F_ = 23.7 Hz, 1C), 116.02 (d, d: *J*_C-F_ = 22.0 Hz, 2C), 92.26 (d, 1C), 81.12 (t, 1C), 53.72 (s, 1C) ppm. **^19^F NMR** (283 MHz, CDCl_3_): δ **=** -104.10 (s, 1F), -111.76 (s, 1F) ppm. **IR** (Reflexion)**:** ṽ = 3075, 2239, 1957, 1601, 1573, 1509, 1472, 1443, 1414, 1314, 1266, 1239, 1229, 1169, 1127, 1069, 943, 858, 823, 769, 696 cm^-1^. **HR-MS** (EI(+)): *m/z* = 299.0573, calcd. for [M]^+^ = [C_17_H_11_F_2_NS]^+^: 299.0575; **m.p.** = 80.4-81.6 °C.

**2-(4-Fluorophenyl)-2-(o-tolylthio)penta-3,4-dienenitrile (5e)**

The product was prepared according to **GP4**, using 49.4 mg of **1a** (0.20 mmol, 1.00 eq.) and 325 mg of **4e** (2.00 mmol, 10.0 eq.) in 4 mL of DCM. Purification by flash column chromatography gave the product as a yellow oil (37.6 mg, 64%).

**R_f_** (SiO_2_, PE/Tol, 1:3): 0.60; **^1^H NMR** (300 MHz, CDCl_3_): δ = 7.52-7.59 (m, 3H), 7.25-7.37 (m, 2H), 7.14-7.20 (m, 1H), 7.01-7.11 (m, 2H), 5.60 (t, *J* = 6.5 Hz, 1H), 5.03 (dd, *J* = 12.0, 6.5 Hz, 1H), 4.79 (dd, *J* = 12.0, 6.5 Hz, 1H), 2.43 (s, 3H) ppm. **^13^C NMR** (75 MHz, CDCl_3_): δ = 207.60 (s, 1C), 162.93 (s, d, *J*_C-F_ = 249.6 Hz, 1C), 144.64 (s, 1C), 138.67 (d, 1C), 132.23 (s, d: *J*_C-F_ = 3.2 Hz, 1C), 130.92 (d, 1C), 130.83 (d, 1C), 129.47 (s, 1C), 128.89 (d, d: *J*_C-F_ = 8.4 Hz, 2C), 126.50 (d, 1C), 118.27 (s, 1C), 115.89 (d, d: *J*_C-F_ = 22.0 Hz, 2C), 92.18 (d, 1C), 81.09 (t, 1C), 53.09 (s, 1C), 21.26 (q, 1C) ppm. **^19^F NMR** (283 MHz, CDCl_3_): δ **=** -112.11 (s, 1F) ppm. **IR** (Reflexion)**:** ṽ = 3062, 2926, 2855, 2237, 1953, 1732, 1600, 1508, 1470, 1411, 1380, 1305, 1238, 1164, 1103, 1060, 1015, 859, 787, 756, 711 cm^-1^. **HR-MS** (EI(+)): *m/z* = 295.0848, calcd. for [M]^+^ = [C_18_H_14_FNS]^+^: 295.0826.

**2-((3-Chlorophenyl)thio)-2-(4-fluorophenyl)penta-3,4-dienenitrile (5f)**

The product was prepared according to **GP4**, using 49.4 mg of **1a** (0.20 mmol, 1.00 eq.) and 365 mg of **4f** (2.00 mmol, 10.0 eq.) in 4 mL of DCM. Purification by flash column chromatography gave the product as a yellow oil (34.5 mg, 55%).

**R_f_** (SiO_2_, PE/Tol, 1:2): 0.56; **^1^H NMR** (500 MHz, CDCl_3_): δ = 7.55-7.57 (m, 2H), 7.41-7.44 (m, 3H), 7.28-7.31 (m, 1H), 7.07-7.10 (m, 2H), 5.59 (t, *J* = 6.5 Hz, 1H), 5.08 (dd, *J* = 12.2, 6.5 Hz, 1H), 4.88 (dd, *J* = 12.2, 6.4 Hz, 1H) ppm. **^13^C NMR** (126 MHz, CDCl_3_): δ = 207.69 (s, 1C), 163.01 (s, d, *J*_C-F_ = 250.2 Hz, 1C), 136.91 (d, 1C), 135.33 (d, 1C), 134.52 (s, 1C), 131.93 (s, 1C), 131.69 (s, d: *J*_C-F_ = 3.4 Hz, 1C), 130.88 (d, 1C), 130.17 (d, 1C), 128.90 (d, d: *J*_C-F_ = 8.7 Hz, 2C), 117.90 (s, 1C), 116.07 (d, d: *J*_C-F_ = 22.0 Hz, 2C), 92.26 (d, 1C), 81.48 (t, 1C), 53.46 (s, 1C) ppm. **^19^F NMR** (283 MHz, CDCl_3_): δ **=** -111.65 (s, 1F) ppm. **IR** (Reflexion)**:** ṽ = 3067, 2237, 1952, 1600, 1563, 1509, 1461, 1399, 1292, 1238, 1164, 1117, 1072, 1015, 835, 780, 682 cm^-1^. **HR-MS** (EI(+)): *m/z* = 315.0241, calcd. for [M]^+^ = [C_17_H_11_ClFNS]^+^: 315.0279.

**2-(4-Fluorophenyl)-2-(m-tolylthio)penta-3,4-dienenitrile (5g)**

The product was prepared according to **GP4**, using 49.4 mg of **1a** (0.20 mmol, 1.00 eq.) and 325 mg of **4g** (2.00 mmol, 10.0 eq.) in 4 mL of DCM. Purification by flash column chromatography gave the product as a yellow oil (34.1 mg, 58%).

**R_f_** (SiO_2_, PE/Tol, 1:2): 0.53; **^1^H NMR** (300 MHz, CDCl_3_): δ = 7.53-7.59 (m, 2H), 7.28-7.32 (m, 2H), 7.21-7.24 (m, 2H), 7.03-7.09 (m, 2H), 5.58 (t, *J* = 6.5 Hz, 1H), 5.04 (dd, *J* = 12.0, 6.5 Hz, 1H), 4.82 (dd, *J* = 12.0, 6.5 Hz, 1H), 2.32 (s, 3H) ppm. **^13^C NMR** (75 MHz, CDCl_3_): δ = 207.65 (s, 1C), 162.89 (s, d: *J*_C-F_ = 249.4 Hz, 1C), 138.95 (s, 1C), 137.98 (d, 1C), 134.33 (d, 1C), 132.22 (s, d: *J*_C-F_ = 3.4 Hz, 1C), 131.48 (d, 1C), 129.90 (s, 1C), 128.96 (d, d: *J*_C-F_ = 8.3 Hz, 2C), 128.88 (d, 1C), 118.28 (s, 1C), 115.84 (d, d: *J*_C-F_ = 22.0 Hz, 2C), 92.49 (d, 1C), 81.13 (t, 1C), 53.22 (s, 1C), 21.27 (q, 1C) ppm. **^19^F NMR** (283 MHz, CDCl_3_): δ **=** -112.27 (s, 1F) ppm. **IR** (Reflexion)**:** ṽ = 3056, 2923, 2857, 2236, 1953, 1688, 1599, 1508, 1475, 1411, 1304, 1237, 1164, 1102, 1015, 836, 783, 692 cm^-1^. **HR-MS** (EI(+)): *m/z* = 295.0835, calcd. for [M]^+^ = [C_18_H_14_FNS]^+^: 295.0826.

**2-(4-Fluorophenyl)-3-methyl-2-(phenylthio)penta-3,4-dienenitrile (5h)**

The product was prepared according to **GP4**, using 49.4 mg of **1a** (0.20 mmol, 1.00 eq.) and 325 mg of **4h** (2.00 mmol, 10.0 eq.) in 4 mL of DCM. Purification by flash column chromatography gave the product as a yellow oil (31.6 mg, 54%).

**R_f_** (SiO_2_, PE/Et_2_O, 15:1): 0.45; **^1^H NMR** (400 MHz, CDCl_3_): δ = 7.34-7.39 (m, 3H), 7.30-7.33 (m, 2H), 7.23-7.27 (m, 2H), 6.95-7.01 (m, 2H), 5.07 (dq, *J* = 11.1, 3.0 Hz, 1H), 4.93 (dq, *J* = 11.2, 3.0 Hz, 1H), 1.76 (t, *J* = 3.0 Hz, 3H) ppm. **^13^C NMR** (101 MHz, CDCl_3_): δ = 206.68 (s, 1C), 162.75 (s, d: *J*_C-F_ = 249.1 Hz, 1C), 137.15 (d, 2C), 131.84 (s, d: *J*_C-F_ = 3.2 Hz, 1C), 130.44 (d, 1C), 129.87 (s, 1C), 129.45 (d, d: *J*_C-F_ = 8.4 Hz, 2C), 128.99 (d, 2C), 118.64 (s, 1C), 115.56 (d, d: *J*_C-F_ = 21.9 Hz, 2C), 98.58 (s, 1C), 80.44 (t, 1C), 55.99 (s, 1C), 16.29 (q, 1C) ppm. **^19^F NMR** (283 MHz, CDCl_3_): δ **=** -112.60 (s, 1F) ppm. **IR** (Reflexion)**:** ṽ = 3061, 2988, 2953, 2922, 2856, 2233, 1956, 1892, 1600, 1505, 1475, 1439, 1409, 1373, 1304, 1269, 1235, 1162, 1105, 1069, 1015, 851, 801, 748, 691, 617 cm^-1^. **HR-MS** (EI(+)): *m/z* = 294.0751, calcd. for [M-H]^+^ = [C_18_H_13_FNS]^+^: 294.0747.

**2-(4-Fluorophenyl)-3-phenyl-2-(phenylthio)penta-3,4-dienenitrile (5i)**

The product was prepared according to **GP4**, using 49.4 mg of **1a** (0.20 mmol, 1.00 eq.) and 449 mg of **4i** (2.00 mmol, 10.0 eq.) in 4 mL of DCM. Purification by flash column chromatography gave the product as a yellow solid (42.8 mg, 60%).

**R_f_** (SiO_2_, PE/Tol, 1:3): 0.50; **^1^H NMR** (400 MHz, CDCl_3_): δ = 7.35-7.39 (m, 1H), 7.30-7.34 (m, 2H), 7.23-7.28 (m, 4H), 7.16-7.22 (m, 5H), 6.88-6.94 (m, 2H), 5.50 (d, *J* = 12.7 Hz, 1H), 5.40 (d, *J* = 12.7 Hz, 1H) ppm. **^13^C NMR** (101 MHz, CDCl_3_): δ = 208.90 (s, 1C), 162.66 (s, d: *J*_C-F_ = 249.2 Hz, 1C), 137.47 (d, 2C), 132.85 (s, 1C), 132.43 (s, d: *J*_C-F_ = 3.3 Hz, 1C), 130.59 (d, 1C), 129.78 (s, 1C), 129.48 (d, d: *J*_C-F_ = 8.4 Hz, 2C), 129.00 (d, 2C), 128.49 (d, 2C), 128.31 (d, 2C), 127.98 (d, 1C), 118.88 (s, 1C), 115.59 (d, d: *J*_C-F_ = 22.0 Hz, 2C), 105.62 (s, 1C), 83.18 (t, 1C), 53.82 (s, 1C) ppm. **^19^F NMR** (283 MHz, CDCl_3_): δ **=** -112.52 (s, 1F) ppm. **IR** (Reflexion)**:** ṽ = 3059, 2232, 1939, 1892, 1732, 1601, 1506, 1475, 1440, 1410, 1304, 1237, 1162, 1105, 1069, 1015, 1002, 915, 865, 842, 808, 751, 694, 647 cm^-1^. **HR-MS** (EI(+)): *m/z* = 357.0970, calcd. for [M]^+^ = [C_23_H_16_FNS]^+^: 357.0982; **m.p.** = 83.1-84.1 °C.

**2-(Benzylthio)-2-(4-fluorophenyl)-3-methylenepent-4-enenitrile (7a)**

The product was prepared according to **GP4**, using 49.4 mg of **1a** (0.20 mmol, 1.00 eq.) and 353 mg of **6a** (2.00 mmol, 10.0 eq.) in 4 mL of DCM. Purification by flash column chromatography gave the product as a yellow oil (34.1 mg, 55%).

**R_f_** (SiO_2_, PE/Et_2_O, 15:1): 0.44; **^1^H NMR** (400 MHz, CDCl_3_): δ = 7.56-7.61 (m, 2H), 7.24-7.33 (m, 5H), 7.05-7.11 (m, 2H), 6.23 (dd, *J* = 17.3, 11.0 Hz, 1H), 5.72 (s, 1H), 5.69 (s, 1H), 5.54 (dd, *J* = 17.3, 1.0 Hz, 1H), 5.20 (dd, *J* = 11.0, 1.0 Hz, 1H), 3.87 (s, 2H) ppm. **^13^C NMR** (101 MHz, CDCl_3_): δ = 162.96 (s, d: *J*_C-F_ = 249.6 Hz, 1C), 141.88 (s, 1C), 134.90 (s, 1C), 132.59 (d, 1C), 131.22 (s, d: *J*_C-F_ = 3.3 Hz, 1C), 129.61 (d, 2C), 129.41 (d, d: *J*_C-F_ = 8.5 Hz, 2C), 128.87 (d, 2C), 127.88 (d, 1C), 118.89 (s, 1C), 118.55 (t, 1C), 116.90 (t, 1C), 116.13 (d, d: *J*_C-F_ = 22.0 Hz, 2C), 54.31 (s, 1C), 37.10 (t, 1C) ppm. **^19^F NMR** (283 MHz, CDCl_3_): δ **=** -112.12 (s, 1F) ppm. **IR** (Reflexion)**:** ṽ = 3064, 3030, 2927, 2231, 1601, 1505, 1454, 1410, 1304, 1236, 1163, 1106, 1070, 1015, 984, 921, 830, 776, 710 cm^-1^. **HR-MS** (EI(+)): *m/z* = 309.0995, calcd. for [M]^+^ = [C_19_H_16_FNS]^+^: 309.0982.

**2-(4-Fluorophenyl)-3-methylene-2-(phenylthio)pent-4-enenitrile (7b)**

The product was prepared according to **GP4**, using 49.4 mg of **1a** (0.20 mmol, 1.00 eq.) and 325 mg of **6b** (2.00 mmol, 10.0 eq.) in 4 mL of DCM. Purification by flash column chromatography gave the product as a yellow oil (22.3 mg, 38%). The product slowly decomposes.

**R_f_** (SiO_2_, PE/Et_2_O, 15:1): 0.49; **^1^H NMR** (400 MHz, CDCl_3_): δ = 7.39-7.47 (m, 5H), 7.29-7.33 (m, 2H), 7.00-7.06 (m, 2H), 6.24 (dd, *J* = 17.4, 11.1 Hz, 1H), 5.59 (s, 1H), 5.43-5.48 (m, 2H), 5.20 (d, *J* = 11.1 Hz, 1H) ppm. **^13^C NMR** (101 MHz, CDCl_3_): δ = 162.82 (s, d: *J*_C-F_ = 249.5 Hz, 1C), 141.57 (s, 1C), 137.21 (d, 2C), 133.08 (d, 1C), 131.46 (s, d: *J*_C-F_ = 3.3 Hz, 1C), 130.64 (d, 1C), 129.63 (d, d: *J*_C-F_ = 8.4 Hz, 2C), 129.25 (s, 1C), 129.14 (d, 2C), 118.93 (s, 1C), 118.44 (t, 1C), 117.40 (t, 1C), 115.86 (d, d: *J*_C-F_ = 22.0 Hz, 2C), 56.97 (s, 1C) ppm. **^19^F NMR** (283 MHz, CDCl_3_): δ **=** -112.33 (s, 1F) ppm. **IR** (Reflexion)**:** ṽ = 3071, 2233, 2211, 1893, 1686, 1602, 1576, 1507, 1461, 1399, 1304, 1239, 1164, 1117, 1084, 1072, 1015, 997, 923, 881, 839, 784, 683, 664 cm^-1^. **HR-MS** (EI(+)): *m/z* = 295.0821, calcd. for [M]^+^ = [C_18_H_14_FNS]^+^: 295.0826.

**2-(4-Fluorophenyl)-3-methylene-2-(o-tolylthio)pent-4-enenitrile (7c)**

The product was prepared according to **GP4**, using 49.4 mg of **1a** (0.20 mmol, 1.00 eq.) and 352 mg of **6c** (2.00 mmol, 10.0 eq.) in 4 mL of DCM. Purification by flash column chromatography gave the product as a yellow oil (17.6 mg, 28%). The product slowly decomposes.

**R_f_** (SiO_2_, PE/Et_2_O, 15:1): 0.50; **^1^H NMR** (400 MHz, CDCl_3_): δ = 7.37-7.43 (m, 3H), 7.27-7.31 (m, 1H), 7.22-7.24 (m, 1H), 7.10-7.14 (m, 1H), 6.98-7.03 (m, 2H), 6.24 (dd, *J* = 17.4, 11.0 Hz, 1H), 5.68 (s, 1H), 5.59 (s, 1H), 5.43 (d, *J* = 17.4 Hz, 1H), 5.19 (d, *J* = 11.1 Hz, 1H), 2.33 (s, 3H) ppm. **^13^C NMR** (101 MHz, CDCl_3_): δ = 162.86 (s, d: *J*_C-F_ = 249.5 Hz, 1C), 144.62 (s, 1C), 141.79 (s, 1C), 138.09 (d, 1C), 133.38 (d, 1C), 131.75 (s, d: *J*_C-F_ = 3.4 Hz, 1C), 130.92 (d, 1C), 130.64 (d, 1C), 129.64 (d, d: *J*_C-F_ = 8.5 Hz, 2C), 128.42 (s, 1C), 126.48 (d, 1C), 118.77 (s, 1C), 118.40 (t, 1C), 117.63 (t, 1C), 115.79 (d, d: *J*_C-F_ = 21.8 Hz, 2C), 56.32 (s, 1C), 21.03 (q, 1C) ppm. **^19^F NMR** (283 MHz, CDCl_3_): δ **=** -112.44 (s, 1F) ppm. **IR** (Reflexion)**:** ṽ = 3060, 3013, 2925, 2232, 2211, 1600, 1506, 1469, 1380, 1304, 1235, 1162, 1106, 1015, 985, 919, 836, 754 cm^-1^. **HR-MS** (EI(+)): *m/z* = 309.0965, calcd. for [M]^+^ = [C_19_H_16_FNS]^+^: 309.0982.

**2-((3-Chlorophenyl)thio)-2-(4-fluorophenyl)-3-methylenepent-4-enenitrile (7d)**

The product was prepared according to **GP4**, using 49.4 mg of **1a** (0.20 mmol, 1.00 eq.) and 392 mg of **6d** (2.00 mmol, 10.0 eq.) in 4 mL of DCM. Purification by flash column chromatography gave the product as a yellow oil (26.8 mg, 41%). The product slowly decomposes.

**R_f_** (SiO_2_, PE/Et_2_O, 15:1): 0.48; **^1^H NMR** (400 MHz, CDCl_3_): δ = 7.43-7.48 (m, 2H), 7.36-7.40 (m, 3H), 7.25-7.29 (m, 1H), 7.02-7.09 (m, 2H), 6.22 (dd, *J* = 17.4, 11.1 Hz, 1H), 5.63 (s, 1H), 5.45-5.49 (m, 2H), 5.22 (d, *J* = 11.1 Hz, 1H) ppm. **^13^C NMR** (101 MHz, CDCl_3_): δ = 162.98 (s, d: *J*_C-F_ = 250.1 Hz, 1C), 141.45 (s, 1C), 136.60 (d, 1C), 135.10 (d, 1C), 134.59 (s, 1C), 132.91 (d, 1C), 131.13 (s, d: *J*_C-F_ = 3.2 Hz, 1C), 131.13 (s, 1C), 130.82 (d, 1C), 130.18 (d, 1C), 129.65 (d, d: *J*_C-F_ = 8.5 Hz, 2C), 118.76 (t 1C), 118.64 (s, 1C), 117.62 (t, 1C), 116.02 (d, d: *J*_C-F_ = 22.1 Hz, 2C), 57.14 (s, 1C) ppm. **^19^F NMR** (283 MHz, CDCl_3_): δ **=** -111.85 (s, 1F) ppm. **IR** (Reflexion)**:** ṽ = 3069, 2232, 2211, 1686, 1600, 1575, 1506, 1462, 1400, 1304, 1237, 1162, 1117, 1084, 1072, 1015, 985, 924, 840, 784, 682, 664 cm^-1^. **HR-MS** (EI(+)): *m/z* = 329.0438, calcd. for [M]^+^ = [C_18_H_13_ClFNS]^+^: 329.0436.

**2-((4-Chlorophenyl)thio)-2-(4-fluorophenyl)-3-methylenepent-4-enenitrile (7e)**

The product was prepared according to **GP4**, using 49.4 mg of **1a** (0.20 mmol, 1.00 eq.) and 392 mg of **6e** (2.00 mmol, 10.0 eq.) in 4 mL of DCM. Purification by flash column chromatography gave the product as a yellow oil (27.6 mg, 42%). The product slowly decomposes.

**R_f_** (SiO_2_, PE/Et_2_O, 15:1): 0.48; **^1^H NMR** (400 MHz, CDCl_3_): δ = 7.43-7.48 (m, 2H), 7.35-7.38 (m, 2H), 7.28-7.31 (m, 2H), 7.02-7.07 (m, 2H), 6.22 (dd, *J* = 17.4, 11.1 Hz, 1H), 5.61 (s, 1H), 5.43-5.48 (s, 2H), 5.21 (d, *J* = 11.0 Hz, 1H) ppm. **^13^C NMR** (101 MHz, CDCl_3_): δ = 162.89 (s, d: *J*_C-F_ = 249.9 Hz, 1C), 141.31 (s, 1C), 138.42 (d, 2C), 137.43 (s, 1C), 132.87 (d, 1C), 131.15 (s, d: *J*_C-F_ = 3.4 Hz, 1C), 129.62 (d, d: *J*_C-F_ = 8.7 Hz, 2C), 129.46 (d, 2C), 127.67 (s, 1C), 118.71 (s, 1C), 118.63 (t, 1C), 117.54 (t, 1C), 116.01 (d, d: *J*_C-F_ = 22.1 Hz, 2C), 57.14 (s, 1C) ppm. **^19^F NMR** (283 MHz, CDCl_3_): δ **=** -111.90 (s, 1F) ppm. **IR** (Reflexion)**:** ṽ = 3090, 2232, 1899, 1602, 1573, 1506, 1475, 1410, 1389, 1304, 1237, 1163, 1094, 1014, 985, 919, 823, 748 cm^-1^. **HR-MS** (EI(+)): *m/z* = 329.0427, calcd. for [M]^+^ = [C_18_H_13_ClFNS]^+^: 329.0436.

**2-((4-(Tert-butyl)phenyl)thio)-2-(4-fluorophenyl)-3-methylenepent-4-enenitrile (7f)**

The product was prepared according to **GP4**, using 49.4 mg of **1a** (0.20 mmol, 1.00 eq.) and 436 mg of **6f** (2.00 mmol, 10.0 eq.) in 4 mL of DCM. Purification by flash column chromatography gave the product as a yellow oil (23.8 mg, 34%). The product slowly decomposes.

**R_f_** (SiO_2_, PE/Et_2_O, 15:1): 0.60; **^1^H NMR** (400 MHz, CDCl_3_): δ = 7.41-7.48 (m, 2H), 7.30-7.37 (m, 4H), 6.98-7.03 (m, 2H), 6.24 (dd, *J* = 17.4, 11.1 Hz, 1H), 5.60 (s, 1H), 5.50 (s, 1H), 5.45 (d, *J* = 17.4 Hz, 1H), 5.19 (d, *J* = 11.0 Hz, 1H), 1.30 (s, 9H) ppm. **^13^C NMR** (101 MHz, CDCl_3_): δ = 162.80 (s, d: *J*_C-F_ = 249.4 Hz, 1C), 154.10 (s, 1C), 141.61 (s, 1C), 136.94 (d, 2C), 133.22 (d, 1C), 131.60 (s, d: *J*_C-F_ = 3.4 Hz, 1C), 129.64 (d, d: *J*_C-F_ = 8.5 Hz, 2C), 126.21 (d, 2C), 125.75 (s, 1C), 119.08 (s, 1C), 118.33 (t, 1C), 117.46 (t, 1C), 115.77 (d, d: *J*_C-F_ = 22.0 Hz, 2C), 56.97 (s, 1C), 34.95 (s, 1C), 31.29 (q, 3C) ppm. **^19^F NMR** (283 MHz, CDCl_3_): δ **=** -112.53 (s, 1F) ppm. **IR** (Reflexion)**:** ṽ = 3078, 2966, 2905, 2869, 2211, 1687, 1601, 1504, 1462, 1397, 1364, 1305, 1268, 1239, 1163, 1118, 1087, 1014, 985, 921, 833, 732, 645 cm^-1^. **HR-MS** (EI(+)): *m/z* = 351.1444, calcd. for [M]^+^ = [C_22_H_22_FNS]^+^: 351.1452.

**2-(4-Fluorophenyl)-2-(heptylthio)-3-methylenepent-4-enenitrile (7g)**

The product was prepared according to **GP4**, using 49.4 mg of **1a** (0.20 mmol, 1.00 eq.) and 368 mg of **6g** (2.00 mmol, 10.0 eq.) in 4 mL of DCM. Purification by flash column chromatography gave the product as a yellow oil (38.6 mg, 61%).

**R_f_** (SiO_2_, PE/Et_2_O, 15:1): 0.60; **^1^H NMR** (400 MHz, CDCl_3_): δ = 7.54-7.59 (m, 2H), 7.04-7.11 (m, 2H), 6.18 (dd, *J* = 17.3, 11.1 Hz, 1H), 5.67 (s, 1H), 5.64 (s, 1H), 5.49 (dd, *J* = 17.4, 1.0 Hz, 1H), 5.16 (dd, *J* = 11.0, 1.0 Hz, 1H), 2.64 (t, *J* = 7.4 Hz, 2H), 1.59-1.66 (m, 2H), 1.34-1.41 (m, 2H), 1.22-1.32 (m, 6H), 0.86-0.89 (m, 3H) ppm. **^13^C NMR** (101 MHz, CDCl_3_): δ = 162.89 (s, d: *J*_C-F_ = 249.3 Hz, 1C), 142.14 (s, 1C), 132.68 (d, 1C), 131.71 (s, d: *J*_C-F_ = 3.3 Hz, 1C), 129.39 (d, d: *J*_C-F_ = 8.5 Hz, 2C), 119.17 (s, 1C), 118.30 (t, 1C), 116.51 (t, 1C), 116.02 (d, d: *J*_C-F_ = 21.8 Hz, 2C), 53.70 (s, 1C), 32.25 (t, 1C), 31.75 (t, 1C), 29.08 (t, 1C), 28.92 (t, 1C), 28.02 (t, 1C), 22.69 (t, 1C), 14.17 (q, 1C) ppm. **^19^F NMR** (283 MHz, CDCl_3_): δ **=** -112.44 (s, 1F) ppm. **IR** (Reflexion)**:** ṽ = 2956, 2928, 2856, 2232, 1893, 1603, 1506, 1466, 1431, 1378, 1304, 1237, 1163, 1106, 1015, 984, 920, 830, 723 cm^-1^. **HR-MS** (EI(+)): *m/z* = 317.1583, calcd. for [M]^+^ = [C_19_H_24_FNS]^+^: 317.1608.

**2-(4-Fluorophenyl)-2-(phenylthio)pent-4-enal (8)**

According to the literature,^[14]^ to a solution of **3aa** (28.3 mg, 0.10 mmol, 1.00 eq.) in 1 mL of dry DCM was added DIBAL-H (1M in toluene 0.12 mmol, 1.20 eq.) -78 °C. After stirring at 25 °C for 16 h, the reaction mixture was quenched by addition of saturated aqueous NH_4_Cl solution, extracted with Et_2_O, washed with brine, dried over anhydrous MgSO_4_, filtered, and concentrated. Purification by flash column chromatography gave the product as a yellow oil (23.1 mg, 81%).

**R_f_** (SiO_2_, PE/Et_2_O, 20:1): 0.32; **^1^H NMR** (400 MHz, CDCl_3_): δ = 9.52 (s, 1H), 7.36-7.42 (m, 5H), 7.28-7.33 (m, 2H), 7.06-7.12 (m, 2H), 5.63 (ddt, *J* = 17.2, 10.2, 6.9 Hz, 1H), 5.01 (dq, *J* = 10.3, 1.3 Hz, 1H), 4.91 (dq, *J* = 17.0, 1.5 Hz, 1H), 2.68 (ddt, *J* = 14.9, 6.7, 1.4 Hz, 1H), 2.59 (ddt, *J* = 15.0, 7.1, 1.4 Hz, 1H) ppm. **^13^C NMR** (101 MHz, CDCl_3_): δ = 191.61 (d, 1C), 162.53 (s, d: *J*_C-F_ = 248.3 Hz, 1C), 137.25 (d, 2C), 132.33 (d, 1C), 131.53 (s, d: *J*_C-F_ = 3.4 Hz, 1C), 130.24 (d, d: *J*_C-F_ = 8.3 Hz, 2C), 130.07 (d, 1C), 129.26 (d, 2C), 129.06 (s, 1C), 119.28 (t, 1C), 115.87 (d, d: *J*_C-F_ = 21.4 Hz, 2C), 65.97 (s, 1C), 38.45 (t, 1C) ppm. **^19^F NMR** (283 MHz, CDCl_3_): δ **=** -113.15 (s, 1F) ppm. **IR** (Reflexion)**:** ṽ = 3085, 2830, 1893, 1854, 1709, 1639, 1602, 1509, 1474, 1439, 1410, 1233, 1165, 1108, 1013, 925, 842, 799, 754, 707, 694 cm^-1^. **HR-MS** (EI(+)): *m/z* = 286.0818, calcd. for [M]^+^ = [C_17_H_15_FOS]^+^: 286.0822.

**2-(4-Fluorophenyl)penta-2,4-dienenitrile (9)**

According to the literature,^[15]^ to a stirred solution of compound **3aa** (28.3 mg, 0.10 mmol, 1.00 eq.) in DCM (1 mL) under N_2_ atmosphere and at -78 °C, *m*-CPBA (27.0 mg, *ca.* 70-75% purity, 0.11 mmol, 1.10 eq.) in DCM (1 mL) was slowly added under those conditions. The reaction mixture was stirred at -78 °C for 1.5 h. When the reaction completed, the reaction was quenched with 10% Na_2_S_2_O_5_ (aq) and then NaHCO_3_ (saturated solution) was added, the resulting mixture was extracted with DCM (3 x 5 mL), washed with brine (3 mL), dried over anhydrous MgSO_4_, filtered, and concentrated. Purification by flash column chromatography gave the product as a white powder (14.7 mg, 85%).

**R_f_** (SiO_2_, PE/EA, 15:1): 0.35; **^1^H NMR** (400 MHz, CDCl_3_): δ = 7.55-7.60 (m, 2H), 7.17 (d, *J* = 11.1 Hz, 1H), 7.06-7.14 (m, 2H), 6.92-7.01 (m, 1H), 5.74 (dt, *J* = 16.7, 1.0 Hz, 1H), 5.66 (dt, *J* = 10.1, 1.1 Hz, 1H) ppm. **^13^C{^19^F} NMR** (126 MHz, CDCl_3_): δ = 163.45 (s, 1C), 141.79 (d, 1C), 133.63 (d, 1C), 129.22 (s, 1C), 127.81 (d, 2C), 126.63 (t, 1C), 116.41 (s, 1C), 116.35 (d, 2C), 113.79 (s, 1C) ppm. **^19^F NMR** (283 MHz, CDCl_3_): δ **=** -111.13 (s, 1F) ppm. **IR** (Reflexion)**:** ṽ = 2928, 2215, 1883, 1604, 1510, 1405, 1326, 1239, 1165, 1105, 990, 936, 837, 668 cm^-1^. **HR-MS** (EI(+)): *m/z* = 173.0654, calcd. for [M]^+^ = [C_11_H_8_FN]^+^: 173.0635. **m.p.** = 58.9-59.7 °C.

# 4. Mechanistic Experiments

**(1) Crossover experiment**:

Alkynyl triazene **1a** (24.7 mg, 0.10 mmol, 1.00 eq.) and a 1:1 mixture of allyl sulfide **2f** (164 mg, 1.00 mmol, 10.0 eq.) and **2u** (164 mg, 1.00 mmol, 10.0 eq.) were dissolved in DCM (2 mL), the reaction mixture was stirred at room temperature under UV irradiation for 16 h. The solvent was removed under reduced pressure and the residue was analysed by a) high resolution mass spectrometry using EI(+) and b) crude ^19^F NMR spectra.


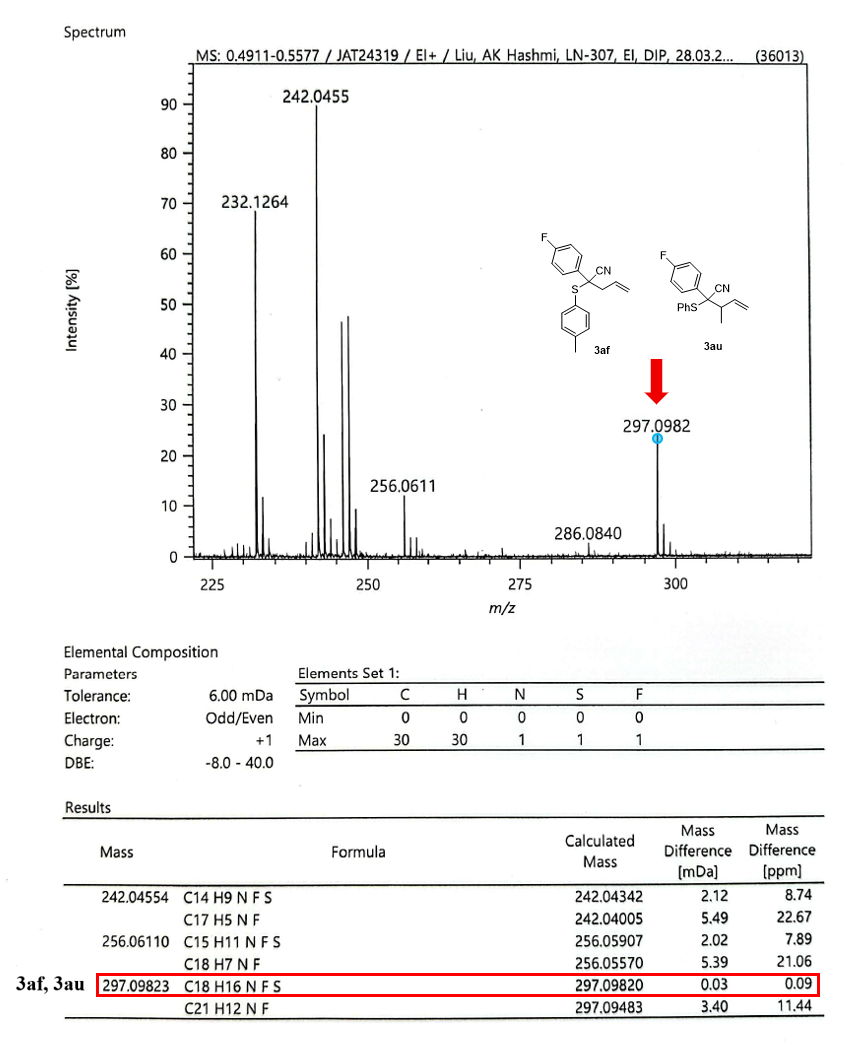


**Figure S1**: HRMS spectrum using EI(+) of the crossover experiment with highlighted peak of reaction products **3af** and **3au**.


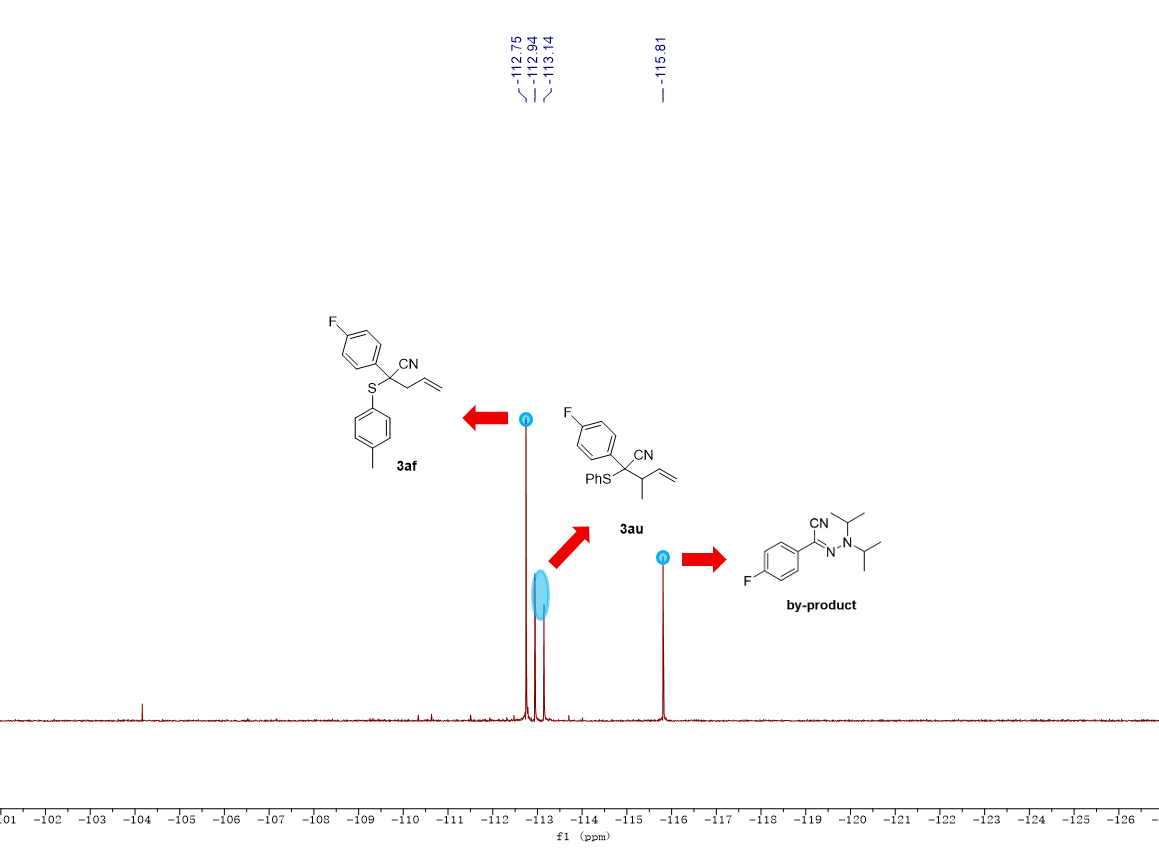


**Figure S2**: Crude ^19^F NMR spectra of the crossover experiment with highlighted peak of reaction products **3af** and **3au**.

**(2) Control experiments**:

To investigate the extent of carbene dimerization in this reaction, we performed several control experiments. First, alkynyl triazene **1a** was irradiated with UV-light in DCM in the absence of the sulfide **2a**, and HRMS with crude ¹⁹F NMR analyses revealed that, in addition to the corresponding hydrazone (by-product A), a carbene dimerization product (3,6-difluorophenanthrene-9,10-dicarbonitrile) was also formed, which has been reported in our previous work.^[1]^ When 2.0 equiv of **2a** was employed, only a trace of carbene dimerization product (by-product B) was observed. Upon successive additions of 5.0 equiv and 10.0 equiv of **2a**, carbene dimerization product (by-product B) became nearly undetectable, and the proportion of hydrazone (by-product A) decreased accordingly, resulting in an improved yield of product **3aa** (see Figures S3 and S4).


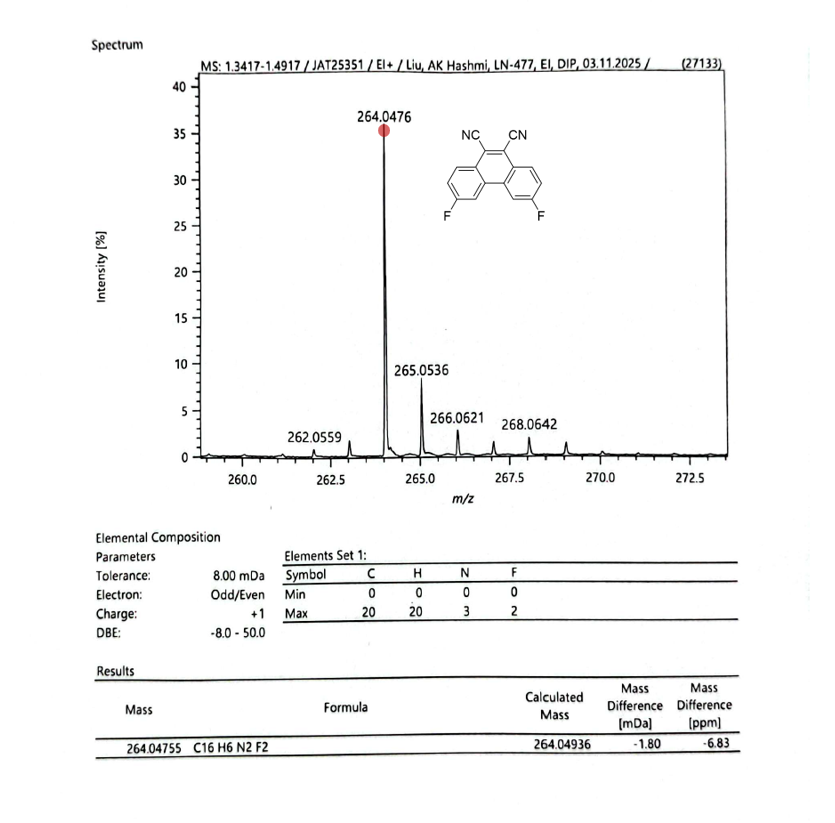


**Figure S3**: HRMS spectrum of 3,6-difluorophenanthrene-9,10-dicarbonitrile (C_16_H_6_N_2_F_2_) in the reaction solution detected using EI(+).


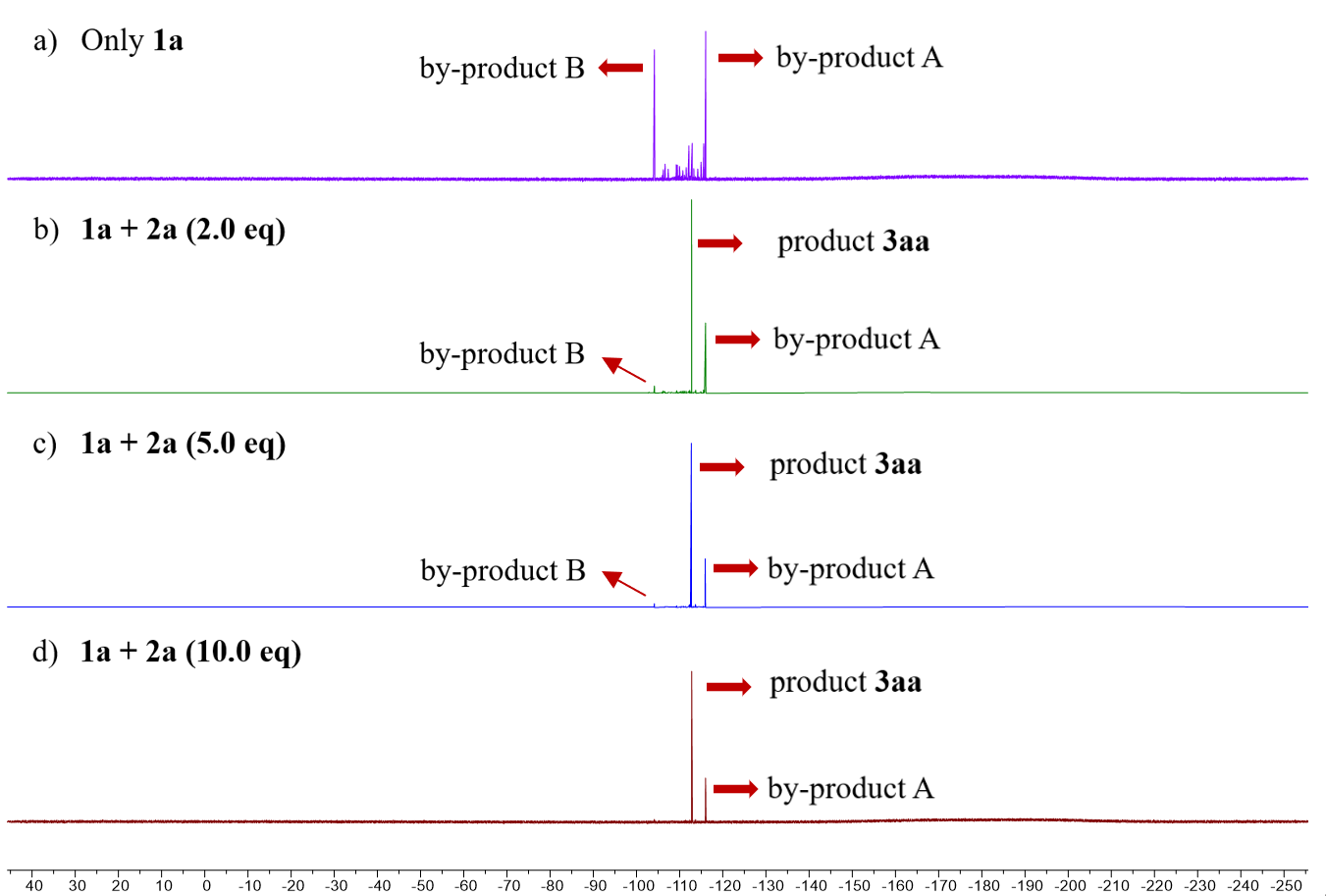


**Figure S4**: Crude ^19^F NMR spectra of the control experiments.

# 5. Computational Details

Unless explicitly stated otherwise, all calculations in this work were performed using ORCA 6.1.^[16]^ Standard parameters were used throughout. Optimized structures were obtained at the ωB97X-D4/def2-QZVPP level of theory. ^[17-25]^ Harmonic analysis at the same level of theory was used to confirm the absence of imaginary frequencies in all reactant, product and intermediate molecules in the reaction mechanism, while the presence of exactly one imaginary frequency was confirmed for the determined transition state. Final energy evaluations were performed at the ωB97M(2)/def2-QZVPPD^[24, 26-27]^ level of theory using Q-Chem 6.3^[28]^ and include a self-consistent solvent correction using the conductor-like polarizable continuum model (CPCM)^[29]^ with solvent parameters for DCM as implemented in Q-Chem. The resolution of identity (RI) approximation was used for the MP2 correction in these calculations using RIMP2-def2-QZVPPD^[30]^ as an auxiliary basis set. Increased self-consistent field convergence criteria (DIIS error norm < ${10}^{-8}$) were employed. Zero-point vibrational energy corrections were calculated using ωB97M-D4/def2-QZVPPD with ORCA 6.1.

The absence of a barrier was confirmed by a relaxed surface scan of the carbon-sulfur bond of structure II employing 30 evenly spaced points from a bond distance of 1.678 Å to 3.5 Å. To ensure that this result was not a method artifact, we performed this scan both with ωB97X-D4/def2-TZVPP and with r²SCAN-3c/def2-mTZVPP.^[31]^ To elucidate whether this reaction can also take place via the triplet ground state, we attempted to perform the same scan on the lowest triplet state of the molecule. However, we found that optimisation of the lowest triplet state (starting from the optimised singlet ground state structure) resulted in fragmentation of the molecule ejecting an allyl radical. The resulting structure is shown in figure S5.


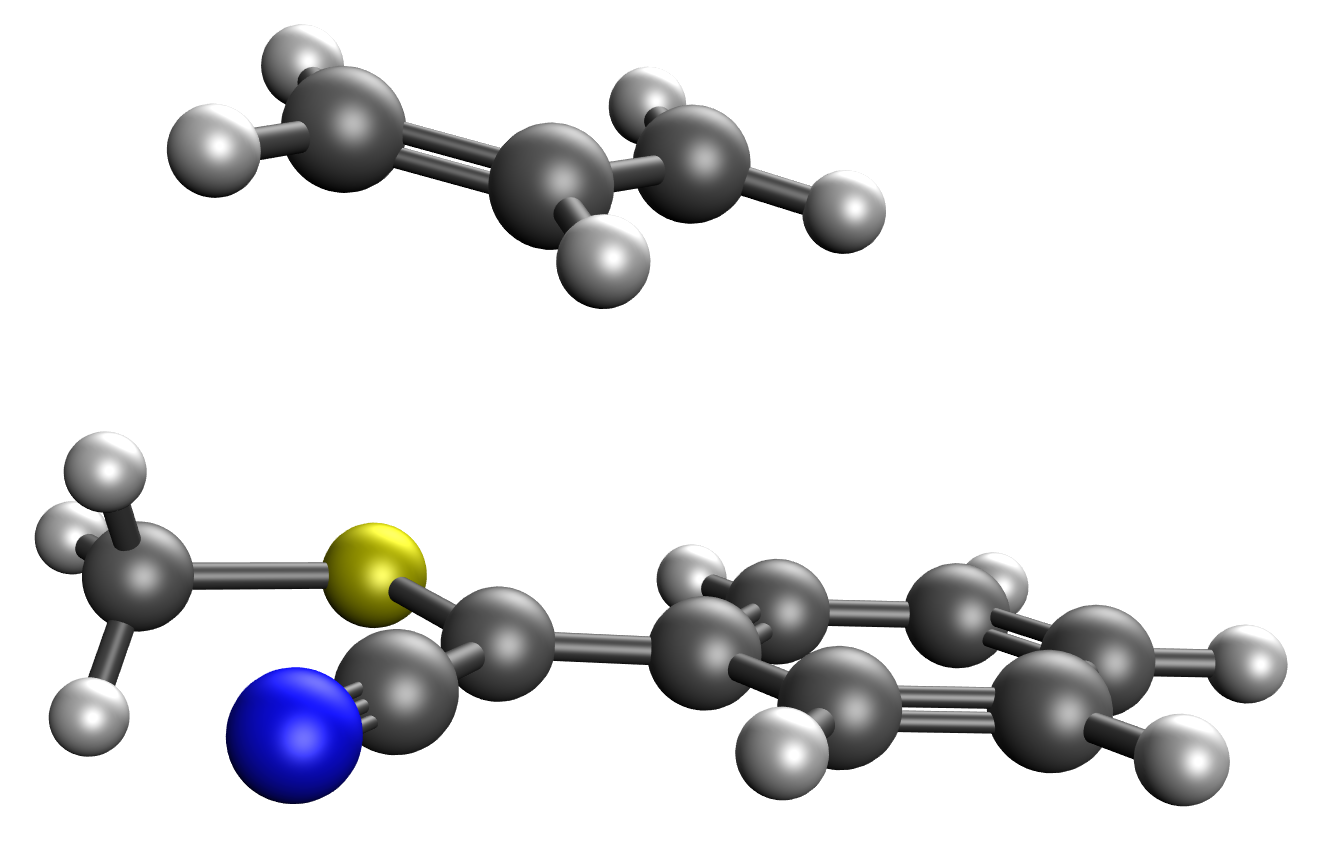


**Figure S5**: Optimized structure of the lowest triplet state of structure II obtained at ωB97X-D4/def2-QVZPP level of theory.

All obtained, optimized structures are given below, including their associated single-point energies and zero-point vibrational corrections. All energies are given in Hartree, XYZ coordinates are provided in Angstrom.

| Structure | ${}^{3}$[I] |
| --- | --- |
| Energy (ωB97M(2)/def2-QZVPPD/CPCM) | -362.7497169798 |
| ZVPE (ωB97M-D4/def2-QZVPPD) | 0.06811388 |
| C -2.01281104668653 1.54301722365570 0.00470916954648  C -2.26493358620405 0.17335262749126 0.00187315511756  C 0.09851959298747 -0.27828381023524 -0.06005150544298  C 1.68881728826270 1.57069293040198 -0.08714136892987  C -0.71433633716242 2.01339482430921 -0.02452162820836  H -3.28515188305992 -0.18937119413071 0.02487319671008  C -1.20578566114175 -0.73095648764111 -0.03055949280990  H -1.40284485913127 -1.79602254361965 -0.03276509509187  C 0.36670248155995 1.10882798346610 -0.05743919601906  H -0.51166295797926 3.07672719197783 -0.02247778441552  H -2.83699192948174 2.24523189984972 0.02989425829957  C 2.99881737496891 1.37987565220360 -0.12057322523140  N 4.17638632443819 1.29426228410038 -0.15018441643442  H 0.92590319862972 -0.97612258182907 -0.08529206709031 | |

| Structure | ${}^{1}$[I] |
| --- | --- |
| Energy (ωB97M(2)/def2-QZVPPD/CPCM) | -362.7484460534 |
| ZVPE (ωB97M-D4/def2-QZVPPD) | 0.07028691 |
| C -1.10018871319790 0.65216025000339 0.00011881625745  C -1.18303782538474 -0.73711864300213 0.00016969317986  C 1.20522245801425 -0.93256402233815 0.00013966926097  H -2.00243826750628 1.25029423247510 0.00011204975932  C 0.14333811183591 1.25428529154211 0.00008074011398  H 2.10771976362276 -1.53280858701127 0.00014308350713  H 0.25245935973465 2.33133237825729 0.00003954965559  C 2.55937306373520 1.18185269591436 0.00005055652289  C 1.32072479463122 0.47684507926815 0.00009323078147  C -0.03631553177345 -1.53202993617474 0.00018115021280  H -0.12572080703306 -2.61097966328843 0.00022263939082  H -2.15778148542030 -1.21186783127649 0.00019886607634  C 3.72380165749049 0.39768922231580 0.00003004931169  N 4.74642742125126 -0.15125846668499 -0.00001109403032 | |

| Structure | Methyl allyl sulfide |
| --- | --- |
| Energy (ωB97M(2)/def2-QZVPPD/CPCM) | -555.5162739886 |
| ZVPE (ωB97M-D4/def2-QZVPPD) | 0.07976784 |
| C -2.94162671657405 1.10177970701104 -0.18138090616609  S -1.37285455975362 0.32303454489574 -0.59184775890957  H -2.98478462801170 2.12375035809323 -0.55921905572597  H -3.72807097209603 0.51950756172658 -0.66020036238253  H -3.11092174918305 1.10061897790679 0.89595568895416  C -0.25331275918652 1.44169476631825 0.28919515108771  C 1.12331437502641 0.85131766645902 0.30921111061069  H -0.24728814754710 2.41935377363185 -0.19583250820556  H -0.62824303015230 1.55984144607012 1.30917767855719  C 2.16601523248411 1.37969565270379 -0.31434044090478  H 1.23525742089473 -0.07578648591120 0.86439691712594  H 2.07727501232505 2.29718271749236 -0.88669916720953  H 3.14333652177405 0.91432431360243 -0.27447434683166 | |

| Structure | II |
| --- | --- |
| Energy (ωB97M(2)/def2-QZVPPD/CPCM) | -918.3446943683 |
| ZVPE (ωB97M-D4/def2-QZVPPD) | 0.17720217 |
| C -1.65036307113914 0.52220391690881 1.80322828760721  S -1.34705429326836 -0.53441903403299 0.38298552722109  H -1.29560419048193 1.52817816931575 1.58091364340387  H -2.72517712456341 0.52898425889434 1.96990943126520  H -1.13720041991654 0.10975671032750 2.67139215236521  C 0.45904029521927 -0.33969841985769 0.29367028259389  C 1.00075817633729 -1.23673170880885 -0.77355465709971  H 0.66022556520385 0.71209034221626 0.08598369121866  H 0.84687050040431 -0.60342350777504 1.28046147185788  C 1.59243836078495 -0.79053291414893 -1.87134353780161  H 0.87819917457420 -2.30123044538510 -0.60409662278578  H 1.72244525876761 0.27030432017467 -2.05863922103225  H 1.97570839890245 -1.47358793558450 -2.61898815592980  C -1.65630738823674 -2.11082521405087 0.86880667981092  C -0.99958690223982 -2.50414949811092 2.04831046414520  C -2.50040753301417 -3.00821517914693 0.07512739056970  N -0.42004984536641 -2.73876507066404 3.02292156255898  H -4.73643870499419 -5.48547861193922 -1.94356587086473  C -3.05722387013865 -2.64114485499176 -1.15536607802253  H -3.75117444796947 -6.16458414665093 0.23290393870233  C -3.85409481177185 -3.52364627471426 -1.86824031111189  H -2.86703914852922 -1.65785472901212 -1.56879391217175  C -4.11534406592123 -4.79814727063853 -1.38285371245957  C -2.76861921031402 -4.29591816441689 0.55478799896273  H -2.34923064172778 -4.60761257324965 1.50427783736653  C -3.56244574709491 -5.17437972718117 -0.16579519569076  H -4.27179131350605 -3.20936043747690 -2.81805308467901 | |

| Structure | TS(II-III) |
| --- | --- |
| Energy (ωB97M(2)/def2-QZVPPD/CPCM) | -918.3270032931 |
| ZVPE (ωB97M-D4/def2-QZVPPD) | 0.17641735 |
| C 2.76997530555485 -0.01423644499488 1.54260526807048  S 1.30751664304289 0.83490836021608 0.95632268521438  H 2.49797614921334 -0.69296937068366 2.34699855888923  H 3.23360766107086 -0.57036513459893 0.73116791271211  H 3.44682297308360 0.75480534497667 1.91200938921730  C 2.29739245102754 1.62493002311744 -0.91605258255019  C 2.29608780634046 0.43436314275066 -1.66015720240263  H 1.52177662952048 2.35679740260329 -1.11418232868855  H 3.23165321901280 2.05393520960805 -0.56779018083377  C 1.08987277198027 -0.12941136055859 -1.97719407563702  H 3.19084452711460 -0.17907982592109 -1.67817910844451  H 0.20702467270819 0.48785565393549 -2.08685812923143  H 1.02526607192929 -1.12789744232684 -2.39142666694253  C 0.36413263687846 -0.39266158049236 0.25733903790139  C 0.84382299111147 -1.72247535098757 0.36175649016634  C -1.08299711329192 -0.14707516686630 0.11331239991024  N 1.25975624116565 -2.79573429240667 0.44438023138489  H -4.88833780219278 0.49263407404931 -0.35514018223067  C -1.58047260269975 1.15250476217693 -0.01097687389232  H -4.02068904358912 -1.82252998770646 -0.16295582534003  C -2.93932982811585 1.37992899522003 -0.16900333737830  H -0.90516630733031 2.00069217941684 0.01604800640193  C -3.82793429021273 0.31497591095032 -0.22562716989671  C -1.98318148451547 -1.21281748964171 0.05008193227997  H -1.61665187319944 -2.22842456571353 0.13922634341250  C -3.33944701188606 -0.98096618212452 -0.11907113542478  H -3.30314439372134 2.39691613600200 -0.25673145666730 | |

| Structure | III |
| --- | --- |
| Energy (ωB97M(2)/def2-QZVPPD/CPCM) | -918.3968272595 |
| ZVPE (ωB97M-D4/def2-QZVPPD) | 0.17833054 |
| C 1.79038282129302 -2.29849316248806 -0.32246479522150  S 1.88687656799029 -0.99522375913999 0.90996851718815  H 0.77201255582424 -2.67379930132442 -0.41464093197549  H 2.15371062074232 -1.94744599367944 -1.28777949712949  H 2.43739157038004 -3.10154010853420 0.02833190590033  C 0.30557226600010 3.71992694783055 0.20822101724656  C -0.13712978489338 2.55668925244785 0.66082379739643  H -0.37619193908171 4.48855885272493 -0.13487129467654  H 1.36624147420926 3.94251272645083 0.15966785934662  C 0.76243287573399 1.45270105039047 1.13461466098008  H -1.20387315409018 2.35557740092570 0.68389835926585  H 1.78663216825627 1.81356423197226 1.24094406640267  H 0.43421378499371 1.08556764672274 2.10964815459124  C 0.76566384180914 0.23915175845543 0.16856417758942  C 1.35958875337475 0.65855044007230 -1.10832291144324  C -0.64075181717424 -0.31430845192499 -0.03228712266244  N 1.84375534361692 0.97178718578428 -2.10063450043641  H -4.25627476149439 -1.65161047094744 -0.41557515710288  C -1.29657004215668 -0.22334218660260 -1.25238885848985  H -3.08937890710426 -1.81958184485872 1.76553861415178  C -2.59474554522650 -0.70426313295727 -1.38929659207088  H -0.79971280173064 0.22501955727842 -2.10355332437201  C -3.24583045954155 -1.27613368462395 -0.30837572072868  C -1.29731062575296 -0.89423068243334 1.05112990898108  H -0.78392750170127 -0.99019387887464 1.99989046956968  C -2.59113333402775 -1.36949182963518 0.91525623712750  H -3.09325363634855 -0.62861784633146 -2.34810702432795 | |

| Structure | Optimized lowest triplet state of II |
| --- | --- |
| C -1.68724726787923 0.80528813167327 2.07678073573554  S -2.44770116686362 0.01609206718333 0.65332184113625  H -1.83586263208243 1.87476835832652 1.93550491379115  H -2.16644777720486 0.49072123154285 3.00144170448851  H -0.62258611389528 0.58422295301103 2.11089454208837  C 1.33321965926427 -0.71633663180291 0.29247646543555  C 0.82399315107568 -1.84652255609815 -0.31354450468692  H 1.35191150770399 0.23270758028003 -0.23142178629856  H 1.73158843593311 -0.74946429925911 1.29797616701492  C 0.29218360484345 -1.87863837338799 -1.58672230568104  H 0.83151435090268 -2.77142657348771 0.25685290485016  H 0.25030141838272 -0.98127200171170 -2.19321767585387  H -0.11382008856959 -2.78978585059756 -2.00529568849133  C -2.16951627555844 -1.65682516250035 0.96028725442461  C -1.47745050652394 -2.02855956103776 2.12542673318235  C -2.58477333639813 -2.65990700821035 0.00662933826713  N -0.89335042201958 -2.31920949830501 3.08020374377644  H -3.59326925634154 -5.36857954188339 -2.58850969024248  C -3.26797540967917 -2.30899133754876 -1.16788829520338  H -2.39730136866720 -6.01341142004616 -0.51416967017359  C -3.62539022894048 -3.27721204638174 -2.08900317321273  H -3.51501451937308 -1.27313423807760 -1.36380561475651  C -3.31325810686920 -4.61381676237771 -1.86392708086525  C -2.28254013695452 -4.01339613242487 0.22738427599989  H -1.75768394551687 -4.30321328905710 1.12958322623409  C -2.64214592838838 -4.97453680338756 -0.69972151428700  H -4.15084464038027 -2.98774923443345 -2.99114684667227 | |

# 6. References

[1] E. Michel, F. F. Grieser, A. V. Mackenroth, M. Schukin, P. Krämer, S. Tahir, F. Rominger, M. Rudolph, A. S. K. Hashmi, *Angew. Chem. Int. Ed*. **2023**, *62*, e202309274.

[2] S. J. Pye, S. J. Dalgarno, J. M. Chalker, C. L. Raston, *Green Chem.* **2018**, *20*, 118-124.

[3] X. Zhang, B. Lin, J. Chen, J. Chen, Y. Luo, Y. Xia, *Org. Lett.* **2021**, *23*, 819-825.

[4] V. Kanchupalli, L. A. Thorbole, J. Kalepu, D. Joseph, M. Arshad, S. Katukojvala, *Org. Lett.* **2022**, *24*, 3850-3854.

[5] A. Parodi, S. Battaglioli, Y. Liu, M. Monari, M. Marín-Luna, C. Silva-López, M. Bandini, *Chem. Commun.* **2019**, *55*, 9669-9672.

[6] B. Gao, T. Jiang, R. Yang, Q. Yan, X. Liu, Q. Xie, X. Zhang, *J. Org. Chem.* **2022**, *87*, 7895-7904.

[7] H. Zhang, B. Wang, H. Yi, Y. Zhang, J.-B. Wang, *Org. Lett.* **2015**, *17*, 3322-3325.

[8] Y.-D. Hao, J. Liang, Z.-Q. Lin, T.-L. Huang, Y.-D. Xu, L. Guo, Z.-Z. Yang, Y. Wu, *Org. Chem. Front.* **2023**, *10*, 4038-4042.

[9] H. Vaid, A. Sharma, P. Jamwal, P. Sharma, R. Gurubrahamam, *Org. Lett.* **2024**, *26*, 2135-2140.

[10] F.-F. He, S. Jana, R. M. Koenigs, *J. Org. Chem.* **2020**, *85*, 11882-11891.

[11] S. Midya, D. P. Hari, *Chem. Sci.* **2023**, *14*, 13560-13567.

[12] H.-W. Luo, S. Ma, *Eur. J. Org. Chem.* **2013**, 3041-3048.

[13] M.-H. Lin, W.-S. Tsai, L.-Z. Lin, S.-F. Hung, T.-H. Chuang, Y.-J. Su, *J. Org. Chem.* **2011**, *76*, 8518-8523.

[14] Y. Hong, Y. Qin, X. Yang, L. Zhou, Y. Yuan, G. Zhu, *Eur. J. Org. Chem.* **2024**, *27*, e202400405.

[15] F. J. McEvoy, J. D. Albright, *J. Org. Chem.* **1979**, *44*, 4597-4603.

[16] F. Neese, Software update: The ORCA program system—Version 5.0. WIREs Comput Mol Sci. 2022; 12:e1606.

[17] N. Mardirossian, M. Head-Gordon, *Phys. Chem. Chem. Phys.* **2014**, *16*, 9904-9924.

[18] B. Helmich-Paris, B. D. Souza, F. Neese, R. Izsák, *J. Chem. Phys*. **2021**, *155*, 104109.

[19] F. Neese, *J Comput Chem.* **2003**, *24* 1740-1747.

[20] D. Bykov, T. Petrenko, R. Izsák, S. Kossmann, U. Becker, E. Valeev, F. Neese, *Molecular Physics*, **2015**, *113*, 1961-1977.

[21] F. Neese, *J Comput Chem.* **2023**, *44*, 381-396.

[22] E. Caldeweyher, S. Ehlert, A. Hansen, H. Neugebauer, S. Spicher, C. Bannwarth, S. Grimme, *J. Chem. Phys*. **2019**, *150*, 154122.

[23] L. Wittmann, I. Gordiy, M. Friede, B. Helmich-Paris, S. Grimme, A. Hansen, M. Bursch, *Phys. Chem. Chem.* *Phys.* **2024**, *26*, 21379-21394.

[24] F. Weigend, F. Furche, R. Ahlrichs, *J. Chem. Phys.* **2003**, *119*, 12753-12762.

[25] F. Weigend, R. Ahlrichs, *Phys. Chem. Chem. Phys.* **2005**, *7*, 3297-3305.

[26] N. Mardirossian, M. Head-Gordon, *J. Chem. Phys.* **2016**, *144*, 214110.

[27] N. Mardirossian, M. Head-Gordon, *J. Chem. Phys*. **2018**, *148*, 241736.

[28] R. Chakraborty, *J. Chem. Phys*. **2021**, *155*, 084801.

[29] S. Miertuš, E. Scrocco, J. Tomasi, *Chemical Physics* **1981**, *55*, 117-129.

[30] A. Hellweg, D. Rappoport, *Phys. Chem. Chem. Phys.* **2015**, *17*, 1010-1017.

[31] S. Grimme, A. Hansen, S. Ehlert, J.-M. Mewes, *J. Chem. Phys*. **2021**, *154*, 064103.

# 7. NMR Spectra





**Figure S3**. ^1^H NMR (400 MHz, CDCl_3_) of compound 6c.





**Figure S4.** ^13^C NMR (101 MHz, CDCl_3_) of compound 6c.





**Figure S5**. ^1^H NMR (700 MHz, CDCl_3_) of compound 6d.





**Figure S6.** ^13^C NMR (176 MHz, CDCl_3_) of compound 6d.





**Figure S7**. ^1^H NMR (400 MHz, CDCl_3_) of compound 6e.





**Figure S8.** ^13^C NMR (101 MHz, CDCl_3_) of compound 6e.





**Figure S9**. ^1^H NMR (400 MHz, CDCl_3_) of compound 6f.





**Figure S10.** ^13^C NMR (101 MHz, CDCl_3_) of compound 6f.





**Figure S11**. ^1^H NMR (400 MHz, CDCl_3_) of compound 6g.





**Figure S12.** ^13^C NMR (101 MHz, CDCl_3_) of compound 6g.





**Figure S13**. ^1^H NMR (300 MHz, CDCl_3_) of compound 3aa.





**Figure S14.** ^13^C NMR (75 MHz, CDCl_3_) of compound 3aa.





**Figure S15.** ^19^F NMR (283 MHz, CDCl_3_) of compound 3aa.





**Figure S16**. ^1^H NMR (500 MHz, CDCl_3_) of compound 3ba.





**Figure S17.** ^13^C NMR (126 MHz, CDCl_3_) of compound 3ba.





**Figure S18.** ^1^H NMR (600 MHz, CDCl_3_) of compound 3ca.





**Figure S19.** ^13^C NMR (151 MHz, CDCl_3_) of compound 3ca.





**Figure S20.** ^1^H NMR (500 MHz, CDCl_3_) of compound 3da.





**Figure S21.** ^13^C NMR (126 MHz, CDCl_3_) of compound 3da.





**Figure S22.** ^1^H NMR (500 MHz, CDCl_3_) of compound 3fa.





**Figure S23.** ^13^C NMR (126 MHz, CDCl_3_) of compound 3fa.





**Figure S24.** ^1^H NMR (400 MHz, CDCl_3_) of compound 3ga.





**Figure S25.** ^13^C NMR (101 MHz, CDCl_3_) of compound 3ga.





**Figure S26.** ^1^H NMR (400 MHz, CDCl_3_) of compound 3ha.





**Figure S27.** ^13^C NMR (101 MHz, CDCl_3_) of compound 3ha.





**Figure S28.** ^19^F NMR (283 MHz, CDCl_3_) of compound 3ha.





**Figure S29.** ^1^H NMR (300 MHz, CDCl_3_) of compound 3ia.





**Figure S30.** ^13^C NMR (75 MHz, CDCl_3_) of compound 3ia.





**Figure S31.** ^1^H NMR (500 MHz, CDCl_3_) of compound 3ja.





**Figure S32.** ^13^C NMR (126 MHz, CDCl_3_) of compound 3ja.





**Figure S33.** ^19^F NMR (283 MHz, CDCl_3_) of compound 3ja.





**Figure S34.** ^1^H NMR (500 MHz, CDCl_3_) of compound 3ka.





**Figure S35.** ^13^C NMR (126 MHz, CDCl_3_) of compound 3ka.





**Figure S36.** ^1^H NMR (400 MHz, CDCl_3_) of compound 3la.





**Figure S37.** ^13^C NMR (101 MHz, CDCl_3_) of compound 3la.





**Figure S38.** ^1^H NMR (500 MHz, CDCl_3_) of compound 3ma.





**Figure S39.** ^13^C NMR (126 MHz, CDCl_3_) of compound 3ma.





**Figure S40.** ^1^H NMR (300 MHz, CDCl_3_) of compound 3na.





**Figure S41.** ^13^C NMR (75 MHz, CDCl_3_) of compound 3na.





**Figure S42.** ^1^H NMR (500 MHz, CDCl_3_) of compound 3ab.





**Figure S43.** ^13^C NMR (126 MHz, CDCl_3_) of compound 3ab.





**Figure S44.** ^19^F NMR (283 MHz, CDCl_3_) of compound 3ab.





**Figure S45.** ^1^H NMR (400 MHz, CDCl_3_) of compound 3ac.





**Figure S46.** ^13^C NMR (101 MHz, CDCl_3_) of compound 3ac.





**Figure S47.** ^19^F NMR (283 MHz, CDCl_3_) of compound 3ac.





**Figure S48.** ^1^H NMR (600 MHz, CDCl_3_) of compound 3ad.





**Figure S49.** ^13^C NMR (151 MHz, CDCl_3_) of compound 3ad.





**Figure S50.** ^19^F NMR (283 MHz, CDCl_3_) of compound 3ad.





**Figure S51.** ^1^H NMR (500 MHz, CDCl_3_) of compound 3ae.





**Figure S52.** ^13^C NMR (126 MHz, CDCl_3_) of compound 3ae.





**Figure S53.** ^19^F NMR (283 MHz, CDCl_3_) of compound 3ae.





**Figure S54.** ^1^H NMR (500 MHz, CDCl_3_) of compound 3af.





**Figure S55.** ^13^C NMR (126 MHz, CDCl_3_) of compound 3af.





**Figure S56.** ^19^F NMR (283 MHz, CDCl_3_) of compound 3af.





**Figure S57.** ^1^H NMR (400 MHz, CDCl_3_) of compound 3ag.





**Figure S58.** ^13^C NMR (101 MHz, CDCl_3_) of compound 3ag.





**Figure S59.** ^19^F NMR (283 MHz, CDCl_3_) of compound 3ag.





**Figure S60.** ^1^H NMR (400 MHz, CDCl_3_) of compound 3ah.





**Figure S61.** ^13^C NMR (101 MHz, CDCl_3_) of compound 3ah.





**Figure S62.** ^19^F NMR (283 MHz, CDCl_3_) of compound 3ah.





**Figure S63.** ^1^H NMR (300 MHz, CDCl_3_) of compound 3ai.





**Figure S64.** ^13^C NMR (101 MHz, CDCl_3_) of compound 3ai.





**Figure S65.** ^19^F NMR (283 MHz, CDCl_3_) of compound 3ai.





**Figure S66.** ^1^H NMR (500 MHz, CDCl_3_) of compound 3aj.





**Figure S67.** ^13^C NMR (126 MHz, CDCl_3_) of compound 3aj.





**Figure S68.** ^19^F NMR (283 MHz, CDCl_3_) of compound 3aj.





**Figure S69.** ^1^H NMR (300 MHz, CDCl_3_) of compound 3ak.





**Figure S70.** ^13^C NMR (126 MHz, CDCl_3_) of compound 3ak.





**Figure S71.** ^19^F NMR (283 MHz, CDCl_3_) of compound 3ak.





**Figure S72.** ^1^H NMR (500 MHz, CDCl_3_) of compound 3al.





**Figure S73.** ^13^C NMR (126 MHz, CDCl_3_) of compound 3al.





**Figure S74.** ^19^F NMR (283 MHz, CDCl_3_) of compound 3al.





**Figure S75.** ^1^H NMR (400 MHz, CDCl_3_) of compound 3am.





**Figure S76.** ^13^C NMR (101 MHz, CDCl_3_) of compound 3am.





**Figure S77.** ^19^F NMR (283 MHz, CDCl_3_) of compound 3am.





**Figure S78.** ^1^H NMR (500 MHz, CDCl_3_) of compound 3an.





**Figure S79.** ^13^C NMR (126 MHz, CDCl_3_) of compound 3an.





**Figure S80.** ^19^F NMR (283 MHz, CDCl_3_) of compound 3an.





**Figure S81.** ^1^H NMR (500 MHz, CDCl_3_) of compound 3ao.





**Figure S82.** ^13^C NMR (126 MHz, CDCl_3_) of compound 3ao.





**Figure S83.** ^19^F NMR (283 MHz, CDCl_3_) of compound 3ao.





**Figure S84.** ^1^H NMR (500 MHz, CDCl_3_) of compound 3ap.





**Figure S85.** ^13^C NMR (126 MHz, CDCl_3_) of compound 3ap.





**Figure S86.** ^19^F NMR (283 MHz, CDCl_3_) of compound 3ap.





**Figure S87.** ^1^H NMR (300 MHz, CDCl_3_) of compound 3aq.





**Figure S88.** ^13^C NMR (101 MHz, CDCl_3_) of compound 3aq.





**Figure S89.** ^19^F NMR (283 MHz, CDCl_3_) of compound 3aq.





**Figure S90.** ^1^H NMR (500 MHz, CDCl_3_) of compound 3ar.





**Figure S91.** ^13^C NMR (126 MHz, CDCl_3_) of compound 3ar.





**Figure S92.** ^19^F NMR (283 MHz, CDCl_3_) of compound 3ar.





**Figure S93.** ^1^H NMR (300 MHz, CDCl_3_) of compound 3as.





**Figure S94.** ^13^C NMR (101 MHz, CDCl_3_) of compound 3as.





**Figure S95.** ^19^F NMR (283 MHz, CDCl_3_) of compound 3as.





**Figure S96.** ^1^H NMR (500 MHz, CDCl_3_) of compound 3at.





**Figure S97.** ^13^C NMR (126 MHz, CDCl_3_) of compound 3at.

**Figure S98.** ^19^F NMR (283 MHz, CDCl_3_) of compound 3at.

**Figure S99.** ^1^H NMR (400 MHz, CDCl_3_) of compound 3au.

**Figure S100.** ^13^C NMR (101 MHz, CDCl_3_) of compound 3au.

**Figure S101.** ^19^F NMR (283 MHz, CDCl_3_) of compound 3au.

**Figure S102.** ^1^H NMR (500 MHz, CDCl_3_) of compound 3av.

**Figure S103.** ^13^C NMR (126 MHz, CDCl_3_) of compound 3av.

**Figure S104.** ^19^F NMR (283 MHz, CDCl_3_) of compound 3av.

**Figure S105.** ^1^H NMR (500 MHz, CDCl_3_) of compound 3aw.

**Figure S106.** ^13^C NMR (126 MHz, CDCl_3_) of compound 3aw.

**Figure S107.** ^19^F NMR (283 MHz, CDCl_3_) of compound 3aw.

**Figure S108.** ^1^H NMR (400 MHz, CDCl_3_) of compound 3ax.

**Figure S109.** ^13^C NMR (101 MHz, CDCl_3_) of compound 3ax.

**Figure S110.** ^19^F NMR (283 MHz, CDCl_3_) of compound 3ax.

**Figure S111.** ^1^H NMR (300 MHz, CDCl_3_) of compound 5a.

**Figure S112.** ^13^C NMR (101 MHz, CDCl_3_) of compound 5a.

**Figure S113.** ^19^F NMR (283 MHz, CDCl_3_) of compound 5a.

**Figure S114.** ^1^H NMR (300 MHz, CDCl_3_) of compound 5b.

**Figure S115.** ^13^C NMR (101 MHz, CDCl_3_) of compound 5b.

**Figure S116.** ^19^F NMR (283 MHz, CDCl_3_) of compound 5b.

**Figure S117.** ^1^H NMR (300 MHz, CDCl_3_) of compound 5c.

**Figure S118.** ^13^C NMR (75 MHz, CDCl_3_) of compound 5c.

**Figure S119.** ^19^F NMR (283 MHz, CDCl_3_) of compound 5c.

**Figure S120.** ^1^H NMR (500 MHz, CDCl_3_) of compound 5d.

**Figure S121.** ^13^C NMR (126 MHz, CDCl_3_) of compound 5d.

**Figure S122.** ^19^F NMR (283 MHz, CDCl_3_) of compound 5d.

**Figure S123.** ^1^H NMR (300 MHz, CDCl_3_) of compound 5e.

**Figure S124.** ^13^C NMR (75 MHz, CDCl_3_) of compound 5e.

**Figure S125.** ^19^F NMR (283 MHz, CDCl_3_) of compound 5e.

**Figure S126.** ^1^H NMR (500 MHz, CDCl_3_) of compound 5f.

**Figure S127.** ^13^C NMR (126 MHz, CDCl_3_) of compound 5f.

**Figure S128.** ^19^F NMR (283 MHz, CDCl_3_) of compound 5f.

**Figure S129.** ^1^H NMR (300 MHz, CDCl_3_) of compound 5g.

**Figure S130.** ^13^C NMR (75 MHz, CDCl_3_) of compound 5g.

**Figure S131.** ^19^F NMR (283 MHz, CDCl_3_) of compound 5g.

**Figure S132.** ^1^H NMR (400 MHz, CDCl_3_) of compound 5h.

**Figure S133.** ^13^C NMR (101 MHz, CDCl_3_) of compound 5h.

**Figure S134.** ^19^F NMR (283 MHz, CDCl_3_) of compound 5h.

**Figure S135.** ^1^H NMR (400 MHz, CDCl_3_) of compound 5i.

**Figure S136.** ^13^C NMR (101 MHz, CDCl_3_) of compound 5i.

**Figure S137.** ^19^F NMR (283 MHz, CDCl_3_) of compound 5i.

**Figure S138.** ^1^H NMR (400 MHz, CDCl_3_) of compound 7a.

**Figure S139.** ^13^C NMR (101 MHz, CDCl_3_) of compound 7a.

**Figure S140.** ^19^F NMR (283 MHz, CDCl_3_) of compound 7a.

**Figure S141.** ^1^H NMR (400 MHz, CDCl_3_) of compound 7b.

**Figure S142.** ^13^C NMR (101 MHz, CDCl_3_) of compound 7b.

**Figure S143.** ^19^F NMR (283 MHz, CDCl_3_) of compound 7b.

**Figure S144.** ^1^H NMR (400 MHz, CDCl_3_) of compound 7c.

**Figure S145.** ^13^C NMR (101 MHz, CDCl_3_) of compound 7c.

**Figure S146.** ^19^F NMR (283 MHz, CDCl_3_) of compound 7c.

**Figure S147.** ^1^H NMR (400 MHz, CDCl_3_) of compound 7d.

**Figure S148.** ^13^C NMR (101 MHz, CDCl_3_) of compound 7d.

**Figure S149.** ^19^F NMR (283 MHz, CDCl_3_) of compound 7d.

**Figure S150.** ^1^H NMR (400 MHz, CDCl_3_) of compound 7e.

**Figure S151.** ^13^C NMR (101 MHz, CDCl_3_) of compound 7e.

**Figure S152.** ^19^F NMR (283 MHz, CDCl_3_) of compound 7e.

**Figure S153.** ^1^H NMR (400 MHz, CDCl_3_) of compound 7f.

**Figure S154.** ^13^C NMR (101 MHz, CDCl_3_) of compound 7f.

**Figure S155.** ^19^F NMR (283 MHz, CDCl_3_) of compound 7f.

**Figure S156.** ^1^H NMR (400 MHz, CDCl_3_) of compound 7g.

**Figure S157.** ^13^C NMR (101 MHz, CDCl_3_) of compound 7g.

**Figure S158.** ^19^F NMR (283 MHz, CDCl_3_) of compound 7g.

**Figure S159.** ^1^H NMR (400 MHz, CDCl_3_) of compound 8.

**Figure S160.** ^13^C NMR (101 MHz, CDCl_3_) of compound 8.

**Figure S161.** ^19^F NMR (283 MHz, CDCl_3_) of compound 8.

**Figure S162.** ^1^H NMR (400 MHz, CDCl_3_) of compound 9.

**Figure S163.** ^13^C{^19^F} NMR (126 MHz, CDCl_3_) of compound 9.

**Figure S164.** ^19^F NMR (283 MHz, CDCl_3_) of compound 9.

# 8. X-ray Crystal Structure

**Figure S165.** Crystal structure of compound **5d**.

**Table S1**: Crystal data and structure refinement for **5d**.

Identification code ln2

Empirical formula C_17_H_11_F_2_NS

Formula weight 299.33

Temperature 200(2) K

Wavelength 0.71073 Å

Crystal system monoclinic

Space group P2_1_/c

Z 4

Unit cell dimensions a = 8.1675(7) Å α = 90 deg.

b = 16.1888(13) Å β = 108.6897(15) deg.

c = 11.5119(9) Å γ = 90 deg.

Volume 1441.9(2) Å^3^

Density (calculated) 1.38 g/cm^3^

Absorption coefficient 0.24 mm^-1^

Crystal shape column

Crystal size 0.188 x 0.158 x 0.100 mm^3^

Crystal colour colourless

Theta range for data collection 2.3 to 29.3 deg.

Index ranges -11≤h≤11, -22≤k≤22, -15≤l≤15

Reflections collected 16333

Independent reflections 3613 (R(int) = 0.0386)

Observed reflections 2796 (I > 2σ(I))

Absorption correction Semi-empirical from equivalents

Max. and min. transmission 0.96 and 0.92

Refinement method Full-matrix least-squares on F^2^

Data/restraints/parameters 3613 / 2 / 196

Goodness-of-fit on F^2^ 1.04

Final R indices (I>2sigma(I)) R1 = 0.042, wR2 = 0.092

Largest diff. peak and hole 0.28 and -0.22 eÅ^-3^

**Table S2**: Atomic coordinates and equivalent isotropic displacement parameters (Å^2^) for **5d**. U_eq_ is defined as one third of the trace of the orthogonalized U_ij_ tensor.

Atom x y z U_eq_

S1 0.3954(1) 0.5465(1) 0.2862(1) 0.0323(1)

C1 0.5832(2) 0.6153(1) 0.2934(1) 0.0275(3)

C2 0.6627(2) 0.6388(1) 0.4235(2) 0.0299(3)

N3 0.7214(2) 0.6573(1) 0.5238(1) 0.0424(4)

C4 0.5207(2) 0.6924(1) 0.2175(2) 0.0345(4)

H4 0.4792 0.6867 0.1308 0.041

C5 0.5202(2) 0.7651(1) 0.2627(2) 0.0391(4)

C6 0.5183(4) 0.8385(1) 0.3075(2) 0.0714(8)

H6A 0.612(3) 0.8728(13) 0.315(2) 0.086

H6B 0.416(3) 0.8546(15) 0.323(2) 0.086

C11 0.7032(2) 0.5608(1) 0.2490(1) 0.0275(3)

C12 0.6825(2) 0.5539(1) 0.1250(2) 0.0418(4)

H12 0.5920 0.5833 0.0672 0.050

C13 0.7917(2) 0.5048(1) 0.0842(2) 0.0441(4)

H13 0.7770 0.5001 -0.0008 0.053

C14 0.9214(2) 0.4631(1) 0.1695(2) 0.0352(4)

C15 0.9427(2) 0.4657(1) 0.2917(2) 0.0382(4)

H15 1.0311 0.4344 0.3484 0.046

C16 0.8325(2) 0.5150(1) 0.3312(2) 0.0346(4)

H16 0.8457 0.5174 0.4162 0.041

F18 1.0338(1) 0.4178(1) 0.1307(1) 0.0508(3)

C21 0.2585(2) 0.6158(1) 0.3294(1) 0.0287(3)

C22 0.1300(2) 0.6589(1) 0.2421(1) 0.0323(4)

C23 0.0207(2) 0.7134(1) 0.2721(2) 0.0389(4)

H23 -0.0657 0.7423 0.2102 0.047

C24 0.0397(2) 0.7249(1) 0.3948(2) 0.0403(4)

H24 -0.0340 0.7625 0.4175 0.048

C25 0.1646(2) 0.6825(1) 0.4846(2) 0.0390(4)

H25 0.1753 0.6904 0.5685 0.047

C26 0.2742(2) 0.6284(1) 0.4524(2) 0.0339(4)

H26 0.3606 0.5997 0.5146 0.041

F27 0.1099(1) 0.6463(1) 0.1224(1) 0.0475(3)

**Table S3**: Hydrogen coordinates and isotropic displacement parameters (Å^2^) for **5d**.

Atom x y z U_eq_

H4 0.4792 0.6867 0.1308 0.041

H6A 0.612(3) 0.8728(13) 0.315(2) 0.086

H6B 0.416(3) 0.8546(15) 0.323(2) 0.086

H12 0.5920 0.5833 0.0672 0.050

H13 0.7770 0.5001 -0.0008 0.053

H15 1.0311 0.4344 0.3484 0.046

H16 0.8457 0.5174 0.4162 0.041

H23 -0.0657 0.7423 0.2102 0.047

H24 -0.0340 0.7625 0.4175 0.048

H25 0.1753 0.6904 0.5685 0.047

H26 0.3606 0.5997 0.5146 0.041

**Table S4**: Anisotropic displacement parameters (Å^2^) for **5d**. The anisotropic displacement factor exponent takes the form: -2 pi^2^ (h^2^ a^*2^ U_11_ + ... + 2 h k a^*^ b^*^ U_12_)

Atom U_11_ U_22_ U_33_ U_23_ U_13_ U_12_

S1 0.0338(2) 0.0262(2) 0.0367(2) -0.0041(2) 0.0109(2) -0.0037(2)

C1 0.0289(7) 0.0260(8) 0.0255(7) -0.0001(6) 0.0059(6) -0.0017(6)

C2 0.0281(8) 0.0292(8) 0.0315(8) -0.0018(7) 0.0082(6) 0.0009(6)

N3 0.0394(8) 0.0506(10) 0.0336(8) -0.0090(7) 0.0069(6) 0.0020(7)

C4 0.0396(9) 0.0324(9) 0.0306(8) 0.0057(7) 0.0101(7) 0.0024(7)

C5 0.0397(9) 0.0311(9) 0.0420(10) 0.0093(8) 0.0068(8) -0.0016(7)

C6 0.0876(19) 0.0335(12) 0.0714(16) 0.0018(11) -0.0048(14) 0.0040(12)

C11 0.0289(8) 0.0260(8) 0.0265(7) -0.0009(6) 0.0077(6) -0.0031(6)

C12 0.0443(10) 0.0521(12) 0.0257(8) 0.0017(8) 0.0067(7) 0.0116(8)

C13 0.0521(11) 0.0538(12) 0.0277(8) -0.0034(8) 0.0145(8) 0.0052(9)

C14 0.0356(8) 0.0315(9) 0.0423(9) -0.0098(7) 0.0178(7) -0.0038(7)

C15 0.0405(9) 0.0332(9) 0.0371(9) 0.0003(7) 0.0072(7) 0.0068(7)

C16 0.0421(9) 0.0341(9) 0.0253(8) 0.0014(7) 0.0078(7) 0.0049(7)

F18 0.0521(6) 0.0492(7) 0.0577(7) -0.0132(5) 0.0270(6) 0.0062(5)

C21 0.0276(7) 0.0269(8) 0.0308(8) -0.0006(6) 0.0084(6) -0.0049(6)

C22 0.0324(8) 0.0360(9) 0.0266(8) 0.0026(7) 0.0069(6) -0.0062(7)

C23 0.0317(8) 0.0368(10) 0.0453(10) 0.0097(8) 0.0083(7) 0.0010(7)

C24 0.0378(9) 0.0333(9) 0.0537(11) -0.0038(8) 0.0200(8) -0.0012(7)

C25 0.0405(9) 0.0436(10) 0.0344(9) -0.0058(8) 0.0142(7) -0.0056(8)

C26 0.0331(8) 0.0371(9) 0.0295(8) 0.0013(7) 0.0072(7) -0.0036(7)

F27 0.0477(6) 0.0627(7) 0.0275(5) 0.0038(5) 0.0056(4) 0.0003(5)

**Table S5**: Bond lengths (Å) and angles (deg) for **5d**.

S1-C21 1.7636(16)

S1-C1 1.8753(16)

C1-C2 1.478(2)

C1-C4 1.515(2)

C1-C11 1.525(2)

C2-N3 1.139(2)

C4-C5 1.288(2)

C4-H4 0.9500

C5-C6 1.298(3)

C6-H6A 0.927(19)

C6-H6B 0.943(19)

C11-C16 1.386(2)

C11-C12 1.387(2)

C12-C13 1.384(3)

C12-H12 0.9500

C13-C14 1.369(3)

C13-H13 0.9500

C14-F18 1.3578(19)

C14-C15 1.361(2)

C15-C16 1.385(2)

C15-H15 0.9500

C16-H16 0.9500

C21-C22 1.387(2)

C21-C26 1.396(2)

C22-F27 1.3503(18)

C22-C23 1.375(2)

C23-C24 1.383(3)

C23-H23 0.9500

C24-C25 1.381(3)

C24-H24 0.9500

C25-C26 1.385(2)

C25-H25 0.9500

C26-H26 0.9500

C21-S1-C1 101.13(7)

C2-C1-C4 109.28(13)

C2-C1-C11 111.58(12)

C4-C1-C11 114.64(13)

C2-C1-S1 106.60(11)

C4-C1-S1 110.12(11)

C11-C1-S1 104.23(10)

N3-C2-C1 178.89(17)

C5-C4-C1 124.30(15)

C5-C4-H4 117.9

C1-C4-H4 117.9

C4-C5-C6 179.5(2)

C5-C6-H6A 118.0(15)

C5-C6-H6B 116.9(15)

H6A-C6-H6B 125(2)

C16-C11-C12 118.35(15)

C16-C11-C1 120.77(14)

C12-C11-C1 120.86(14)

C13-C12-C11 121.10(16)

C13-C12-H12 119.4

C11-C12-H12 119.4

C14-C13-C12 118.27(16)

C14-C13-H13 120.9

C12-C13-H13 120.9

F18-C14-C15 118.69(16)

F18-C14-C13 118.65(15)

C15-C14-C13 122.66(16)

C14-C15-C16 118.41(16)

C14-C15-H15 120.8

C16-C15-H15 120.8

C15-C16-C11 121.12(15)

C15-C16-H16 119.4

C11-C16-H16 119.4

C22-C21-C26 117.60(15)

C22-C21-S1 121.11(12)

C26-C21-S1 121.28(12)

F27-C22-C23 118.53(15)

F27-C22-C21 118.63(15)

C23-C22-C21 122.83(15)

C22-C23-C24 118.28(16)

C22-C23-H23 120.9

C24-C23-H23 120.9

C25-C24-C23 120.81(17)

C25-C24-H24 119.6

C23-C24-H24 119.6

C24-C25-C26 119.97(16)

C24-C25-H25 120.0

C26-C25-H25 120.0

C25-C26-C21 120.49(15)

C25-C26-H26 119.8

C21-C26-H26 119

**5d**: colourless crystal (column), dimensions 0.188 x 0.158 x 0.100 mm^3^, crystal system monoclinic, space group P2_1_/c, Z=4, a=8.1675(7) Å, b=16.1888(13) Å, c=11.5119(9) Å, alpha=90 deg, beta=108.6897(15) deg, gamma=90 deg, V=1441.9(2) Å^3^, rho=1.379 g/cm^3^, T=200(2) K, Theta_max_= 29.289 deg, radiation MoKα, lambda=0.71073 Å, 0.5 deg omega-scans with CCD area detector, covering the asymmetric unit in reciprocal space with a mean redundancy of 4.09and a completeness of 91.6% to a resolution of 0.73 Å, 16333 reflections measured, 3613 unique (R(int)=0.0386), 2796 observed (I > 2σ(I)), intensities were corrected for Lorentz and polarization effects, an empirical scaling and absorption correction was applied using SADABS^[1]^ based on the Laue symmetry of the reciprocal space, mu=0.24mm^-1^, T_min_=0.92, T_max_=0.96, structure solved with SHELXT-2018/2 (Sheldrick 2015)^[2]^ and refined against F^2^ with a Full-matrix least-squares algorithm using the SHELXL-2019/2 (Sheldrick, 2019) software^[3]^, 196 parameters refined, hydrogen atoms were treated using appropriate riding models, except of the terimal allene hydrogen atoms H6x, which were refined restrained, goodness of fit 1.04 for observed reflections, final residual values R1(F)=0.042, wR(F^2^)=0.092 for observed reflections, residual electron density -0.22 to 0.28 eÅ^-3^. CCDC ...... contains the supplementary crystallographic data for this paper. The data can be obtained free of charge from The Cambridge Crystallographic Data Centre via www.ccdc.cam.ac.uk/structures.

Lit. 1: (SADABS-2016/2 - Bruker AXS area detector scaling and absorption correction)

Krause, L., Herbst-Irmer, R., Sheldrick G.M. & Stalke D., J. Appl. Cryst. 48 (2015) 3-10.

Lit. 2: (SHELXT - Integrated space-group and crystal structure determination)

Sheldrick G. M., Acta Cryst. A71 (2015) 3-8.

Lit. 3: (program SHELXL-2019/2 (Sheldrick, 2019) for structure refinement)

Sheldrick G. M., Acta Cryst. (2015). C71, 3-8

Lit. APEX, APEX2, SMART, SAINT, SAINT-Plus:

Bruker (2007). "Program name(s)". Bruker AXS Inc., Madison, Wisconsin, USA.
